# Synthesis of Novel Artemisinin, Ciprofloxacin, and Norfloxacin Hybrids with Potent Antiplasmodial Activity

**DOI:** 10.3390/antibiotics13020142

**Published:** 2024-02-01

**Authors:** Georgia Vamvoukaki, Antonia I. Antoniou, Michel Baltas, Elisabeth Mouray, Sebastien Charneau, Philippe Grellier, Constantinos M. Athanassopoulos

**Affiliations:** 1 Synthetic Organic Chemistry Laboratory, Department of Chemistry, University of Patras, GR-26504 Patras, Greece; georgiavamv.gv@gmail.com (G.V.); tonadoniou@upatras.gr (A.I.A.); 2 CNRS, LCC (Laboratoire de Chimie, de Coordination), Université de Toulouse, UPS, INPT, 205 Route de Narbonne, BP 44099, CEDEX 4, F-31077 Toulouse, France; 3 MCAM, UMR 7245, Muséum National d’Histoire Naturelle, CNRS, CP52, 63 rue Buffon, F-75005 Paris, France; mouray@mnhn.fr (E.M.); charneau@unb.br (S.C.); philippe.grellier@mnhn.fr (P.G.); 4 Laboratory of Biochemistry and Protein Chemistry, Department of Cell Biology, Institute of Biology, University of Brasilia, Brasilia 70910-900, Brazil

**Keywords:** fluoroquinolones, hybrid, conjugate, structure–activity relationship, antimalarial activity, Pf FcB1 CQ-resistant strain, Pf Dd2 CQ-resistant strain

## Abstract

The synthesis and antiplasmodial evaluation of new hybrids combining the pharmacophore structures of artemisinin, ciprofloxacin or norfloxacin, and 7-chloroquinoline are reported in this study. The first step for all of the syntheses is the obtainment of key piperazine esters intermediates bearing the drugs ciprofloxacin and norfloxacin. Using these platforms, 18 final compounds were synthesized through a multistep procedure with overall yields ranging between 8 and 20%. All compounds were screened for their antiplasmodial activity against the chloroquine-resistant *Plasmodium falciparum* FcB1 strain. Compounds **20**, **21**, **22**, and **28**, bearing an artesunate fragment with ciprofloxacin, exhibited IC_50_ values in the range of 3.5–5.4 nM and excellent selectivity indices. Among the compounds bearing the artesunate moiety on the norfloxacin, two of them, **23** and **24**, afforded IC_50_ values of 1.5 nM and 1.9 nM, respectively. They also showed excellent selectivity indices. The most potent compounds were also evaluated against the CQ-resistant Dd2 strain of *Plasmodium falciparum*, demonstrating that those compounds incorporating the artesunate fragment were the most potent. Finally, the combination of artesunate with either ciprofloxacin or norfloxacin moieties in a single molecular entity proved to substantially enhance the activity and selectivity when compared to the administration of the unconjugated counterparts artesunate/ciprofloxacin and artesunate/norfloxacin.

## 1. Introduction

Malaria is a parasitic disease that is caused by *Plasmodium*. It affects millions of people every year and can lead to death, mainly caused by *Plasmodium falciparum*. There is no efficacious malaria vaccine, and, currently, the WHO recommends as a treatment the use of artemisinin-based combination therapies (ACTs), which involve the combination of an artemisinin (ART) derivative, such as artemether or sodium artesunate (AS), with a partner drug, mostly a quinoline-based drug, with a different mechanism of action [1]. Unfortunately, the parasites have developed resistance to the known antimalarial drugs, and the current frontline antimalarial ART is now under threat. Several research groups have focused on a newer strategy for the discovery of the next generation of antimalarial drugs. More specifically, the combination of two antimalarial drugs in a single hybrid molecule could overcome these limitations, allowing each pharmacophore to act simultaneously on multiple targets [2,3]. Furthermore, these hybrids may improve efficiency, due to their increased cellular uptake in comparison with the cellular uptake of the corresponding parent drugs and act as prodrugs, where the hydrolysis of the ester moiety provides the individual pharmacological activity. Thus, various quinoline- [4,5] and ART-based [6] hybrids have been synthesized in recent years and tested for their activity against different strains of the *P. falciparum*.

In brief, an ART–acridine hybrid **1** [7] was found to be sevenfold more active than chloroquine (CQ) against the *P. falciparum* NF54 strain, whereas an ART–estrogen hybrid **2** [8] exhibited higher antiplasmodial activity than standard drugs against the 3D7 strain. Tsogoeva et al. [9] prepared five ART–quinazoline hybrids and evaluated them against the 3D7 strain. All of them provided very good EC_50_ (half maximal effective concentration), with hybrid **3** being even more active than the clinical drugs dihydroartemisinin (DHA) and CQ, with EC_50_ values of 1.4 ± 0.4 nM, 2.4 ± 0.4 nM, and 9.8 ± 2.8 nM, respectively. Furthermore, another work from the same group [10] provided a small library of ART–CQ and ART–isoCQ hybrids via a click chemistry approach to enhance the potency against CQ resistance and against multidrug-resistant *Plasmodium falciparum* strains. In fact, all these compounds were active with EC_50_ values ranging from 780 pM to 27.5 nM. The ART–CQ hybrid **4** demonstrated significant efficacy (EC_50_ = 1.7–4.5 nM) against all three *P. falciparum* parasite strains (3D7, Dd2, and K1) in comparison to AS (EC_50_ = 5.2–14.4 nM) and was therefore comparable to ACTs.

**Figure 1:**
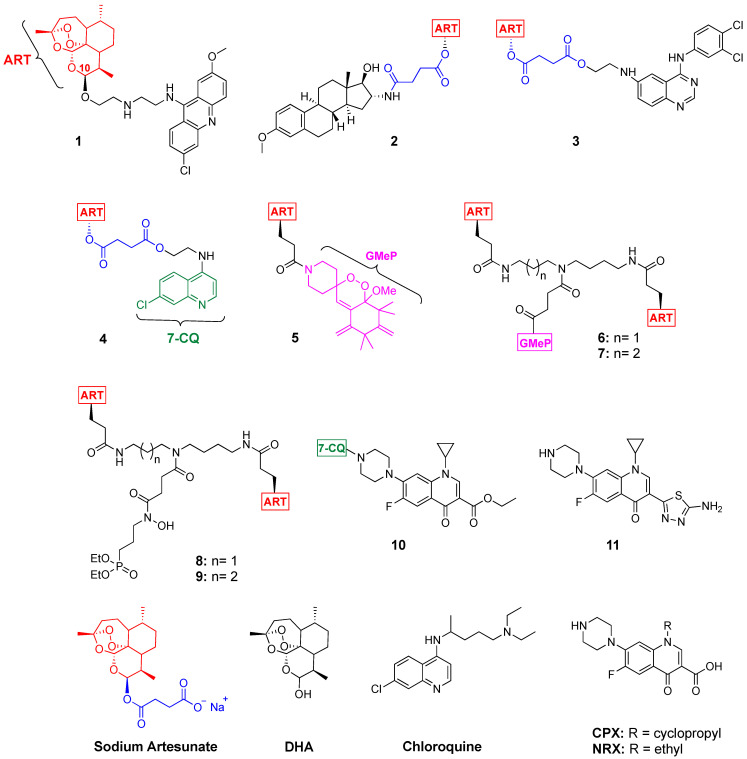
Structures of various hybrids (**1**–**11**) with antimalarial activity and of clinical drugs related to this work.

Recent studies from our group have demonstrated that the combination of ART with a phytohormone endoperoxide G factor analogue (GMeP) and/or polyamines, such as spermidine and homospermidine, led to the discovery of three candidates more potent than ART and CQ [11]. The ART–GMeP hybrid **5** and compounds **6** and **7**, with two units of ART and one of GMeP conjugated through a polyamine linker, exhibited antiplasmodial activities at nM concentrations (IC_50_ values between 2.6 and 10.6 nM) against the CQ-resistant *P. falciparum* strain FcB1, with hybrid **5** being 21 times more active than ART (IC_50_ = 55 ± 13.6 nM). Furthermore, an ART core was also combined with the antibiotic fosmidomycin (FSM), which is also known for its antimalarial activity as it targets the non-mevalonate isoprenoid synthesis pathway that is essential for the malaria parasite [12,13]. The ART–FSM conjugates **8** and **9** exhibited antiplasmodial activity against the FcB1 strain 41.5 and 23.1 times more potent than FSM, respectively (Figure 1).

Another family of compounds with diverse biological activities including antimalarial [14], antibacterial [15,16,17], antitubercular [18,19], antiviral [20,21], and anticancer [22,23,24] activities that has been extensively investigated by many research groups are the quinolones. Since 1958, seven quinolone analogues have been approved by the Food and Drug Administrator (FDA), including two drugs involved in the present work, ciprofloxacin (CPX) and norfloxacin (NRX) [25,26]. CPX is a second-generation fluoroquinolone that was introduced in 1987, and, since then, derivatives [27], analogues [28,29,30], and hybrids [31,32,33] have been reported. Mukhopadhyay et al. [31] were the first to synthesize ciprofloxacin-based hybrids that exhibited nanomolar antimalarial activity. Among them, the CPX–(7-CQ) hybrid **10** is the most potent (IC_50_ = 63.17 ± 1.2 nM and 25.52 ± 4.45 nM against the 3D7 and W2 *P. falciparum* strains, respectively) and is nontoxic to mammalian and bacterial systems. Recently, another group designed and prepared new CPX–1,3,4-thiadiazole hybrids, which they screened to investigate their antimicrobial activities [32]. Among them, hybrid **11** seemed promising with similar activity to the parent drug but lower drug resistance (Figure 1).

**Figure 2:**
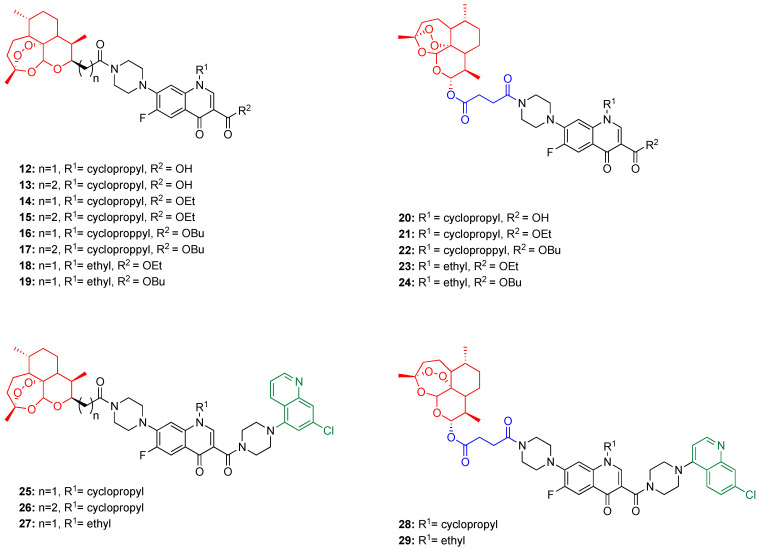
Structures of the synthesized ART-based (**12**–**19** and **25**–**27**) and AS-based (**20**–**24**, **28**, and **29**) hybrids.

In this work, we report the synthesis and the biological evaluation of 18 novel hybrids that combine the pharmacophore structures of ART, CPX or NRX, and 7-chloroquinoline (7-CQ). Structure–activity relationship studies (SARS) of this suitably designed library will allow us to study the following: (a) which one of the drugs CPX or NRX provides higher antiplasmodial activity; (b) how the esterification of the fluorquinolone moiety free carboxylic group, such as ethyl or butyl ester, affects the activity of hybrids; (c) the difference in the antiplasmodial activity between the 10-carba–ART–CPX and 10-carba–ART–NRX hybrids and the corresponding artesunate hybrids, and d) how the introduction of a third pharmacophore through an amide bond between the fluoroquinolone and piperazine-(7-CQ) modifies the activity of the compounds (Figure 2).

## 2. Results and Discussion

### 2.1. Synthesis of the Key Intermediates ***38***–***43***

**Scheme 1:**
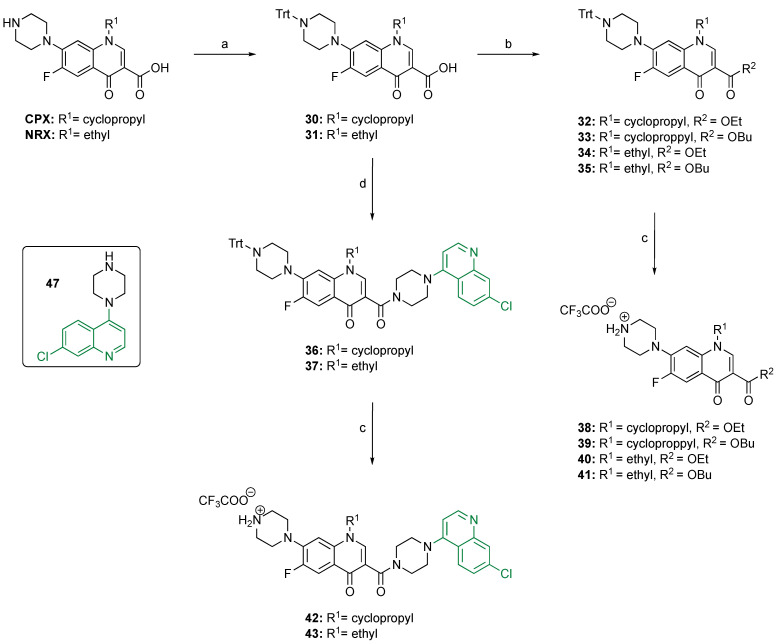
Synthesis of the CPX and NRX intermediate esters **38**–**41** and the intermediate conjugates with quinoline **42** and **43**. Reagents and conditions: (a) TMSCl, Et_3_N, DCM/ CH_3_CN, reflux 1h then TrtCl, rt, 3 h, 79–80%; (b) EtOH or BuOH, DMAP, EDCl, DCM/ DCE, rt, 48 h, 45–65%; (c) 10% TFA in DCM, anisole, rt, 1 h, 90%; and (d) 7-chloro-4-(piperazin-1-yl)quinoline (**47**), TOTU, DIPEA, DMF, rt, 12 h, 61%.

The first step in the synthesis of the key intermediates of drugs CPX and NRX is the protection of the secondary amine of the piperazine fragment after the temporary silylation of the free carboxylic group in a one-pot reaction. Subsequently, the corresponding *N*-trityl-protected analogues **30** and **31** (Scheme 1) were subjected either to a Steglich-type esterification with commercially available alcohols using 1-ethyl-3-(3-dimehtylaminopropyl)carbodiimide hydrochloride (EDC·HCl) in the presence of a catalytic amount of 4-dimehtylaminopyridine (DMAP), affording compounds **32**–**35**, or to an amide bond formation with 7-chloro-4-(piperazin-1-yl)quinoline (**47**) using O-[(Ethoxycarbonyl)cyanomethylenamino]-*N*,*N*,*N′*,*N′*-tetramethyluronium tetrafluoroborate (TOTU) as the coupling reagent in the presence of DIPEA, thereby providing the conjugates **36** and **37** in a 61% yield after purification through flash column chromatography (FCC). Compound **47** was obtained from the commercially available 4,7-dichloro-quinoline via the nucleophilic aromatic substitution of the latter with piperazine according to a published procedure [34]. Finally, the treatment of conjugates **32**–**37** with TFA/DCM/anisole for 1 h at room temperature led to the key intermediates **38**–**42** (Scheme 1).

### 2.2. Synthesis of the ART– and AS– CPX or NRX Hybrids ***14***–***19*** and ***21***–***24***

**Scheme 2:**
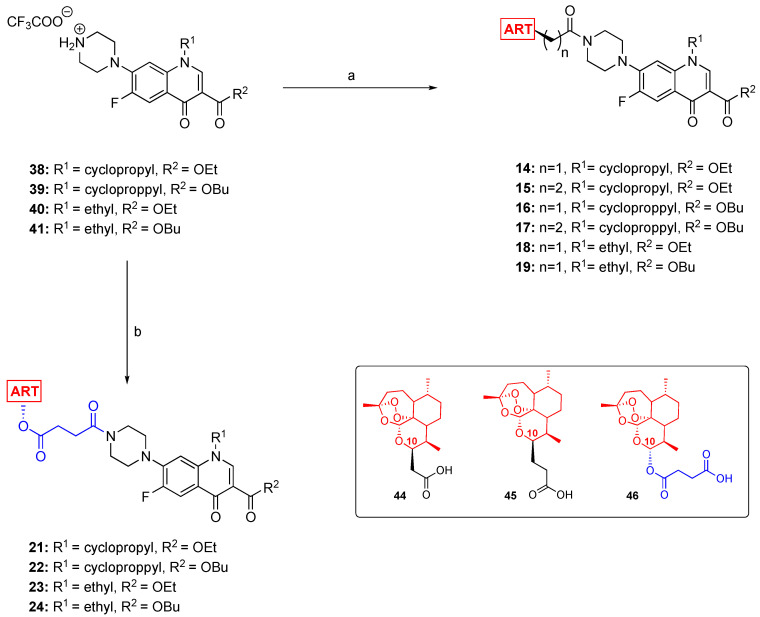
Synthesis of the ART–CPX hybrids (**14**–**17**, **21** and **22**) and the ART–NRX hybrids (**18**, **19**, **23** and **24**). Reagents and conditions: (a) **44** or **45**, BOP or TOTU, DIPEA, DCM or DMF, rt, 2–12 h, 31–76%; and (b) **46**, BOP or TOTU, DIPEA, DCM or DMF, rt, 2–12 h, 56–88%.

The TFA–piperazine salts of esters **38**–**41** were then conjugated through an amide bond either with the ART-derived carboxylic acids **44** and **45** (Scheme 2) or with the artesunate fragment **46** using BOP or TOTU as the coupling reagent in the presence of DIPEA. The desirable hybrid compounds **14**–**19** and **21**–**24** were obtained in good to excellent yields ranging from 56% to 88% after flash column purification. Regarding the synthesis of the ART derivatives **44**–**46**, they were prepared starting from ART according to published procedures [11,35].

### 2.3. Synthesis of the ART–Drug–(7-CQ) Hybrids ***25***–***29***

**Scheme 3:**
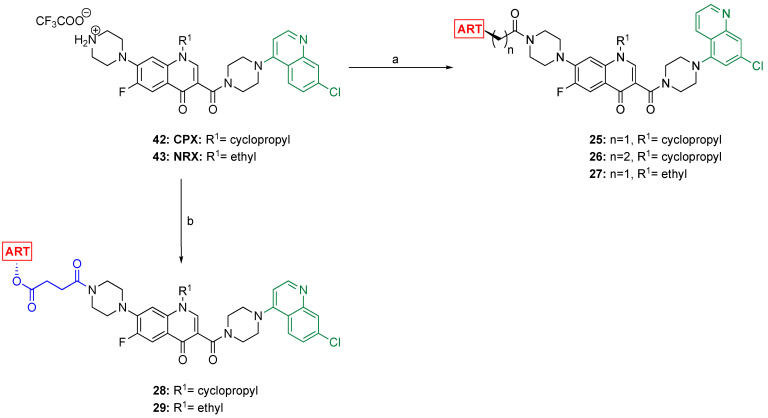
Synthesis of the ART–CPX–(7-CQ) hybrids (**25**, **26** and **28**) and the ART–NRX–(7-CQ) hybrids **27** and **29**. Reagents and conditions: (a) **44** or **45**, TOTU, DIPEA, DMF, rt, 12 h, 50–75%; and (b) **46**, TOTU, DIPEA, DMF, rt, 12 h, 61–64%.

Compounds **42** and **43**, bearing two pharmacophores (a fluoroquinolone and 7-CQ) already tethered through an amide bond, were further combined with one of the three artemisinin derivatives **44**, **45**, and **46**. The reactions were performed in DMF using the TOTU/DIPEA system, which resulted in the final hybrids **25**–**29** after flash column purification in 50–75% yields (Scheme 3).

### 2.4. Synthesis of the ART–CPX and AS–CPX Hybrids ***12***, ***13***, and ***20***

**Scheme 4:**
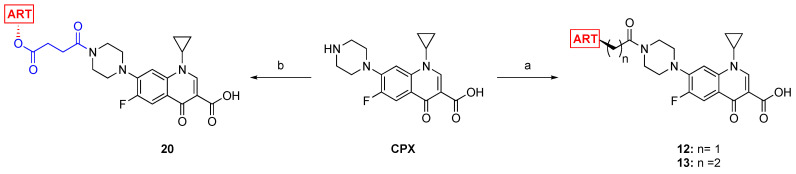
Synthesis of the ART–CPX hybrids **12**, **13**, and **20**. Reagents and conditions: (a) **44** for **12** or **45** for **13,** BOP, DIPEA, DCM, rt, 12 h, 51–55%; and (b) **46**, BOP, DIPEA, DCM, rt, 12 h, 40%.

Finally, for the sake of comparison, condensation occurred between the piperazine ring of the commercially available CPX with the C-10-carba-artemisinin fragments **44** and **45** and the artesunate (**46**), which led to the ART–CPX conjugates **12**, **13**, and **20** (Scheme 4). All reactions were conducted in DCM under peptide coupling conditions using BOP as the activating reagent and DIPEA as a base.

### 2.5. Biological Investigation

The synthesized compounds were screened for their antiplasmodial activity against the CQ-resistant *P. falciparum* FcB1 strain, using AS, CPX, and NRX as control drugs (Figure 1). Moreover, their cytotoxicity was measured upon the primary human fibroblast cell line AB943, which allowed the calculation of their selectivity index (SI). The IC_50_ and SI (the ratio between the IC_50_ of cytotoxicity and the IC_50_ of antiplasmodial activity) values for the hybrids bearing the CRX or the NRX fragment and the drug controls are reported in Table 1 and Table 2, respectively. In addition, for the most interesting compounds, the IC_50_ values were also evaluated against the CQ-resistant Dd2 strain of *P. falciparum*, and, consequently, their SIs were also evaluated and are reported in Table 3 (with the CPX fragment present) and Table 4 (with the NRX fragment present).

CPX hydrochloride (Table 1, entry 1) showed a weak antiplasmodial activity against the FcB1 strain with an IC_50_ value of 54.1 µM. Among the three artemisinin-derived carboxylic acids (Table 1, entries 20–22), artesunate presented the best activity (IC_50_ = 18.5 nM) and a SI > 7042. Among the compounds that did not incorporate the artemisinin fragment (Table 1, entries 14–19), those with the trityl protective group on the piperazine had almost the same IC_50_ values as CPX (Table 1, entries 14, 16), while, surprisingly, the compound **33** bearing a n-Bu ester group was ten times more active (Table 1, entry 15). In addition, when comparing the trifluoroacetate salts of the ethyl and n-Bu esters (compounds **38** and **39**, respectively), we found activity that was >3.9 better for the latter compound (Table 1, entries 17 and 18, respectively). Most importantly, the 7-chloroquinoline-piperazine fragment when incorporated to the CPX (Table 1, entry 19) afforded the best activity among this subgroup of compounds (IC_50_ = 360 nM). In fact, compound **42** was 37 times more potent than compound **39** (lacking the 7-chloroquinoline-piperazine moiety) and 148 times more active than compound **38**.

**Table 1:** Antiplasmodial activity of compounds bearing the CPX fragment against the CQ-resistant *P. falciparum* FcB1 strain, cytotoxicity upon human primary fibroblasts AB943, and selectivity index.

Entry	Compound	IC_50_ FcB1 (nM)	IC_50_ upon Fibroblasts AB943 (µM) ^a^	Selectivity Index (IC_50_ AB943/IC_50_ FcB1)
1	CPX	54,100 ^b^	n.d.	
2	**12**	14.8 +/− 1.9 ^c^	66.1 +/− 5.0	4453
3	**13**	2906 ^b^	n.d.	
4	**14**	121.3 ^b^	n.d.	
5	**15**	1466 ^b^	n.d.	
6	**16**	7.5 +/− 0.6 ^c^	14.9 +/− 3.8	2000
7	**17**	362.0 ^b^	n.d.	
8	**20**	4.0 +/− 0.4 ^c^	41.3 +/− 11.6	10,318
9	**21**	5.4 +/− 0.4 ^c^	52.8 +/− 1.0	9826
10	**22**	3.5 +/− 0.4 ^c^	38.9 +/− 10.	11,303
11	**25**	7.5 +/− 2.5 ^c^	3.9 +/− 2.2	521
12	**26**	13.7 +/− 2.2 ^c^	36.5 +/− 9.4	2668
13	**28**	4.9 +/− 0.4 ^c^	19.6 +/− 7.5	4046
14	**32**	41,900 ^b^	n.d.	
15	**33**	3900 ^b^	n.d.	
16	**36**	26,500 ^b^	n.d.	
17	**38**	53,600 ^b^	n.d.	
18	**39**	13,600 ^b^	n.d.	
19	**42**	359.9 ^b^	n.d.	
20	**44**	401.4 ^b^	n.d.	
21	**45**	264.4 ^b^	n.d.	
22	AS (**46**)	18.5 +/− 3.1 ^c^	>130.0	>7042

n.d.: not determined; ^a^: mean +/− standard deviations of the IC_50_ values determined from at least three independent experiments; ^b^: IC_50_ value determined from the compound tested at the concentrations of 50, 5, and 0.5 µg/mL and 50 and 5 ng/mL in triplicate; and ^c^: mean +/− standard deviations of the IC50 values determined from at least four independent experiments.

Next, we evaluated compounds bearing at least an artemisinin and a CPX moiety (Table 1, entries 2–13). All 4 compounds **20**, **21**, **22**, and **28** derived from the coupling of AS with CPX fragments presented very potent activity with IC_50_ values in the range of 3.5–5.4 nM (Table 1, entries 8, 9, 10, and 13). These values are 3.4–5.2 times lower than AS (**46**) itself (IC_50_ = 18.5 nM). In addition, three of these hybrids (**20**–**22**) presented SIs better than AS (10,286, 9103, and 11,308, respectively).

Concerning the artemisinin-derived carboxylic acids **44** and **45**, they presented IC_50_ values of 401 and 264 nM. When 7-CQ was present in addition to ART and CPX moieties (Table 1, entries 11 and 12), compounds **25** and **26** presented comparable IC_50_ values (7.5 nM and 13.7 nM, respectively). In the absence of 7-chloroquinoline, it is important to note that the n-Bu esters were more potent again than the corresponding ethyl esters. In fact, compound **16** was sixteen times more potent than compound **14** (Table 1, entries 6 and 4), and compound **17** was four times more potent than compound **15** (Table 1, entries 7 and 5). Concerning the acids **12** and **13** (Table 1, entries 2 and 3), we observed a large variation between the IC_50_ values, with compound **12** being around 200 times more potent than compound **13**. Finally, concerning the selectivity indices of this subgroup, we observed very good values for compounds **12**, **16**, and **28** (SI = 4453, 2000, and 4046, respectively), although these were lower in comparison with the best-performing artesunate–CPX derivatives.

For compounds bearing only the NRX fragment, the IC_50_ values were quite poor, ranging from 14 to 84 µM. Nevertheless, we again observed a slightly better activity for the n-Bu esters than for ethyl esters (Table 2, entries 8 vs. 9 and 11 vs. 12). For compounds bearing the artemisinin moiety on the NRX ester derivatives, we observed very potent activities (Table 1, entries 2–5). Here again, coupling with the artesunate moiety afforded tenfold better IC_50_ values than coupling with artemisinin-derived carboxylic acids **44** and **45**. Thus, compounds **23** and **24** (Table 2, entries 4 and 5) presented IC_50_ values of 1.5 nM and 1.9 nM, respectively, with the former having an excellent and much higher SI value (28,382 vs. 16,047). The 7-chloroquinoline moiety, when coupled with NRX alone or with the ART–NRX derivatives, did not contribute to better results, neither for antiplasmodial activity nor for the SIs (Table 2, entries 6, 7, 10, and 13).

**Table 2:** Antiplasmodial activity of compounds bearing the NRX fragment against the CQ-resistant *P. falciparum* FcB1 strain, cytotoxicity upon human primary fibroblasts AB943, and selectivity index.

Entry	Compound	IC_50_ FcB1 (nM)	IC_50_ upon Fibroblast AB943 (µM) ^a^	Selectivity Index (IC_50_ AB943/IC_50_ FcB1
1	NRX	81,410 ^b^	n.d.	
2	**18**	25.5 +/− 7.5 ^c^	54.3 +/− 12.4	2129
3	**19**	13.9 +/− 2.5 ^c^	18.0 +/− 2.3	1294
4	**23**	1.5 +/− 0.1 ^c^	43.7 +/− 14.7	28,382
5	**24**	1.9 +/− 0.3 ^c^	30.3 +/− 9.6	16,047
6	**27**	6.6 +/− 1.5 ^c^	n.d.	
7	**29**	5.0 +/− 0.8 ^c^	30.3 +/− 9.1	6027
8	**34**	84,787 ^b^	n.d.	
9	**35**	26,220 ^b^	n.d.	
10	**37**	184.1 +/− 22.6 ^c^	n.d.	
11	**40**	19,670 ^b^	n.d.	
12	**41**	13,890 ^b^	n.d.	
13	**43**	476.4 +/− 45.5 ^c^	12.7 +/− 4.2	27
14	**44**	401.4 ^b^	n.d.	
15	**45**	264.4 ^b^	n.d.	
16	AS (**46**)	18.5 +/− 3.1 ^c^	>130.0	>7042

n.d.: not determined; ^a^: mean +/− standard deviations of the IC50 values determined from at least three independent experiments; ^b^: IC_50_ value determined from compound tested at the concentrations of 50, 5, and 0.5 µg/mL and 50 and 5 ng/mL in triplicate; and ^c^: mean +/− standard deviations of the IC_50_ values determined from at least four independent experiments.

The most potent compounds in both the CPX and NRX series were also evaluated against the CQ-resistant Dd2 strain of *P. falciparum*; the results are presented in Table 3 and Table 4. AS (**46**) was taken as the reference compound. We can generally observe the same trends in the antiplasmodial activities, and the IC_50_ values are in the same range for the FCB1 and Dd2 strains. The same is also true for the SI values when calculated using the Dd2 IC_50_ values, although they are less high than those for the FcB1 strain. When examining all the evaluated compounds, we can notice that those bearing the artesunate fragment are the most potent.

**Table 3:** Comparison of the antiplasmodial activity of compounds bearing the CPX fragment against the CQ-resistant *P. falciparum* FcB1 and Dd2 strains.

Entry	CPX-Comp.	IC_50_ FcB1 (nM) ^a^	IC_50_ Dd2 (nM) ^a^	IC_50_ upon Fibroblast AB943 (µM) ^a^	Selectivity Index (IC_50_ AB943/IC_50_ FcB1)	Selectivity Index (IC_50_ AB943/IC_50_ Dd2)
1	**12**	14.8 +/− 1.9	32.5 +/− 1.9	66.1 +/− 5.0	4453	2034
2	**16**	7.5 +/− 0.6	24.0 +/− 4.2	14.9 +/− 3.8	2000	622
3	**20**	4.0 +/− 0.4	8.0 +/− 0.4	41.3 +/− 11.6	10,318	5146
4	**21**	5.4 +/− 0.4	16.0 +/− 1.4	52.8 +/− 1.0	9826	3302
5	**22**	3.5 +/− 0.4	13.0 +/− 0.8	38.9 +/− 10.0	11,303	3002
6	**25**	7.5 +/− 2.5	17.7 +/− 2.0	3.9 +/− 2.2	521	220
7	**26**	13.7 +/− 2.2	41.5 +/− 2.5	36.5 +/− 9.4	2668	880
8	**28**	4.9 +/− 0.4	12.4 +/− 0.8	19.6 +/− 7.5	4046	1582
9	AS (**46**)	18.5 +/− 3.1	41.9 +/− 6.7	>130.0	>7042	>3104

^a^: mean +/− standard deviations of the IC_50_ values of at least three independent experiments.

**Table 4:** Comparison of the antiplasmodial activity of compounds bearing the NRX fragment against the CQ-resistant *P. falciparum* FcB1 and Dd2 strains.

Entry	NRX-Comp.	IC_50_ FcB1 (nM) ^a^	IC_50_ Dd2 (nM) ^a^	IC_50_ upon Fibroblast AB943 (µM) ^a^	Selectivity Index (IC_50_ AB943/IC_50_ FcB1)	Selectivity Index (IC_50_ AB943/IC_50_ Dd2)
1	**18**	25.5 +/− 7.5	58.7 +/− 10.8	54.3 +/− 12,4	2129	925
2	**19**	13.9 +/− 2.5	38.4 +/− 7.0	18.0 +/− 2.3	1294	469
3	**23**	1.5 +/− 0.1	3.5 +/− 0.7	43.7 +/− 14.7	28,382	12,488
4	**24**	1.9 +/− 0.3	9.2 +/− 3.5	30.3 +/− 9.6	16,047	3311
5	**27**	6.6 +/− 1.5	28.5 +/− 13.1	n.d.		
6	**29**	5.0 +/− 0.8	18.2 +/− 9.1	30.3 +/− 9.1	6027	1658
7	**37**	184.1 +/− 22.6	307.3 +/− 171.4	n.d.		
8	**43**	476.4 +/− 45.5	482.6 +/− 8.4	12.7 +/− 4.2	27	26
9	AS (**46**)	18.5 +/− 3.1	41.9 +/− 6.7	>130.0	>7042	>3104

n.d.: not determined; and ^a^: mean +/− standard deviations of the IC_50_ values of at least three independent experiments.

In order to clarify the importance of the covalent conjugation of the pharmacophores, for three of them (**22**, **23**, and **24**) we also examined the antiplasmodial activity measured against the FcB1 strain when there is no covalent bond between the artesunate and the CPX or NRX fragment. In this respect, we determined the IC_50_ values of the CPX and NRX fragments alone (compounds **39**, **40**, and **41**) and mixed with AS (**46**) at an equimolar ratio and compared them to the IC_50_ values of the corresponding hybrid compounds (**22, 23**, and **24**) and AS (the results are shown in Table 5). The CPX and NRX fragments (compounds **39**, **40**, and **41**) have very poor antiplasmodial activity in the micromolar range. When mixed with artesunate at an equimolar ratio, the IC_50_ values (expressed as the equivalent AS concentration present in the mixture) were similar to the IC_50_ value of AS alone, indicating no deleterious action of the CPX or NRX fragment by itself on the AS antiplasmodial activity. Most gratifyingly, not only are the excellent IC_50_ values, which are even better than artesunate, maintained in the hybrid compounds **22**, **23**, and **24** but also the cytotoxicity of each of them measured against the human primary fibroblasts AB943 and, consequently, the selectivity indices are much better for the best hybrid compounds than for the parent AS (**46**).

## 3. Materials and Methods

### 3.1. General Methods

All solvents were dried and purified according to the standard procedures prior to use. When required, the reactions were carried out under a dry argon atmosphere in preflamed glassware. Anhydrous Na_2_SO_4_ was used for drying the solutions, and the solvents were then routinely removed at ca. 40 °C under reduced pressure using a rotary vacuum evaporator. All reagents employed in this present work were commercially available and used without further purification. Flash column chromatography (FCC) was performed on silica gel (70–230 and 230–400 mesh, Merck, Darmstadt, Germany) and analytical thin layer chromatography (TLC) on silica 60gel-F254 precoated aluminum foils (0.2 mm film, Merck, Germany). The spots on the TLC plates were visualized with UV light at 254 nm and ninhydrin solution or charring agents. ^1^H NMR spectra were obtained at 600.13 MHz, ^13^C NMR spectra at 150.90 MHz, and ^19^F NMR spectra at 564.63 MHz on a Bruker AVANCEIII HD spectrometer in CHCl_3_. Chemical shifts (*δ*) are indicated in parts per million (ppm) downfield from TMS and referenced to residual undeuterated solvents (7.26 for ^1^H NMR and 77.16 for ^13^C NMR). Copies of the ^1^H and ^13^C spectra of all the final compounds are reported in the Supplementary Material (Figures S1–S36). The referencing of the ^19^F NMR spectra was calculated with the instrument using the default methods. Coupling constants (*J*) are reported in hertz. Electrospray ionization (ESI) mass spectra were recorded at 30V on a Waters Micromass Platform LC spectrometer (Waters, Wilmslow, UK) using MeOH as the solvent. Melting points were determined with a Buchi SMP-20 apparatus (Buchi, Flawil, Switzerland) and are uncorrected.

### 3.2. Experimental Procedures

#### 3.2.1. Synthesis of the N-Trt-Protected CPX **30** and NRX **31**

To a stirred suspension of commercially available CPX or NRX (6.0 mmol) in CHCl_3_/MeCN (5:1, 9 mL), TMSCl (0.84 mL, 5.8 mmol) was added at room temperature, and the reaction mixture was heated to reflux for 1 h. It was subsequently cooled to 0 °C. The addition of Et_3_N (1.84 mL, 13.2 mmol) and of four equal portions of TrtCl (1.74 g, 6.2 mmol) every 15 min was undertaken, and the reaction mixture was stirred at 0 °C for another 3 h. Upon completion of the reaction, MeOH (1.2 mL, 30 mmol) was added, and the precipitate was filtered under vacuo with CHCl_3_. The filtrate was concentrated to approximately one-fifth of its original volume under a reduced pressure, diluted with CHCl_3_, and acidified with 5% aqueous citric acid. The organic layer was washed with water and brine, dried over anhydrous Na_2_SO_4_, and concentrated to dryness under vacuum. The residues thus obtained were subjected to FCC, affording the corresponding Trt-protected analogs **30** and **31**.

1-cyclopropyl-6-fluoro-4-oxo-7-(4-tritylpiperazin-1-yl)-1,4-dihydroquinoline-3-carboxylic acid (**30**): Pale yellow solid (2.72 g, 79%); R_f_ (PhMe/AcOΕt 4:6): 0.25; ^1^H NMR (600 MHz, CDCl_3_) *δ*: 15.02 (s, 1H), 8.74 (s, 1H), 7.94 (d, *J* = 13.2 Hz, 1H), 7.54 (br s, 5H), 7.34 (d, *J* = 7.2 Hz, 1H), 7.30 (t, *J* = 7.8 Hz, 7H), 7.20 (t, *J* = 7.2 Hz, 3H), 3.56–3.46 (m, 4H), 2.98–2.15 (m, 4H), 1.60 (s, 1H), 1.40 (q, *J* = 7.2 Hz, 2H), and 1.18 (q, *J* = 6.6 Hz, 2H); ^13^C NMR (150 MHz, CDCl_3_) *δ*: 177.2, 167.2, 154.6, 147.5, 139.2, 129.8, 128.1, 127.9, 126.5, 113.6, 112.6, 112.4, 108.2, 104.7, 50.6, 47.8, 36.4, and 8.4; and ESI-MS (30 eV) *m*/*z*: [M+H]^+^ Calcd for C_36_H_33_FN_3_O_3_^+^ 574.25, Found 574.37, [M+Νa]^+^ Calcd for C_36_H_32_FN_3_NaO_3_^+^ 596.23, Found 596.76.

1-ethyl-6-fluoro-4-oxo-7-(4-tritylpiperazin-1-yl)-1,4-dihydroquinoline-3-carboxylic acid (**31**): Pale yellow solid (2.69 g, 80%); R_f_ (PhMe/AcOΕt 3:7): 0.29; ^1^H NMR (600 MHz, CDCl_3_) *δ*: 15.10 (s, 1H), 8.66 (s, 1H), 8.01(d, *J* = 13.1 Hz, 1H), 7.52 (br s, 5H), 7.30 (t, *J* = 7.9 Hz, 7H), 7.19 (t, *J* = 7.2 Hz, 3H), 6.82 (d, *J* = 6.7 Hz, 1H), 4.32 (q, *J* = 7.2 Hz, 2H), 3.48 (q, *J* = 6.8 Hz, 4H), 1.59 (t, *J* = 7.2 Hz, 4H), and 1.57 (s, 3H); ^13^C NMR (150 MHz, CDCl_3_) *δ*: 177.2, 167.4, 147.2, 137.3, 129.5, 127.9, 126.5, 112.9, 108.6, 103.6, 50.7, 49.8, 47.8, and 14.6; and ESI-MS (30 eV) *m*/*z*: [M+H]^+^ Calcd for C_35_H_33_FN_3_O_3_^+^ 562.25, Found 562.37, [M+Νa]^+^ Calcd for C_35_H_32_FN_3_NaO_3_^+^ 584.23, Found 584.63, [M+Κ]^+^ Calcd for C_35_H_32_FKN_3_O_3_^+^ 600.21, Found 600.12.

#### 3.2.2. Synthesis of the Ethyl and Butyl Ester of CPX (**32** and **33**) and NRX (**34** and **35**)

To an ice-cold solution of **30** or **31** (1.0 mmol) in anhydrous DCE (4.6 mL), absolute EtOH or n-BuOH (3.0 mmol) and a catalytic amount of DMAP (12 mg, 0.1 mmol) were added. Then, a solution of EDCI (0.21 g, 1.4 mmol) in dry DCM (2.9 mL) was added dropwise, and the reaction mixture was stirred for 24–48 h at room temperature. Upon completion of the reaction, the mixture was diluted with DCM and washed sequentially with 5% aqueous citric acid, water, 1N aqueous NaHCO_3_, water, and brine; dried over anhydrous Na_2_SO_4_; and concentrated to dryness under vacuum. The corresponding esters were afforded after FCC purification.

Ethyl 1-cyclopropyl-6-fluoro-4-oxo-7-(4-tritylpiperazin-1-yl)-1,4-dihydroquinoline-3-carboxylate (**32**): Yellow solid (391 mg, 65%); R_f_ (PhMe/AcOΕt 1:1): 0.29; ^1^H NMR (600 MHz, CDCl_3_) *δ*: 8.52 (s,1H), 7.99 (d, *J* = 13.5 Hz, 1H), 7.53 (br s, 5H), 7.31–7.25 (m, 6H), 7.20–7.16 (m, 3H), 4.38 (q, *J* = 14.2 Hz, 2H), 3.43 (septet, *J* = 3.1 Hz, 4H), 1.58 (br s, 3H), 1.40 (t, *J* = 7.1 Hz, 3H), 1.36–1.32 (m, 2H), 1.26 (s, 2H), and 1.15–1.12 (m, 2H); ^13^C NMR (150 MHz, CDCl_3_) *δ*: 168.0, 166.2, 148.2, 135.9, 129.5, 129.2, 128.4, 128.1, 127.8, 126.4, 110.7, 106.2, 61.0, 50.7, 47.8, 34.5, 29.8, and 8.3; and ESI-MS (30 eV) *m*/*z*: [M+H]^+^ Calcd for C_38_H_37_FN_3_O_3_^+^ 602.28, Found 602.29, [M+Νa]^+^ Calcd for C_38_H_36_FN_3_NaO_3_^+^ 624.26, Found 624.28, [M+Κ]^+^ Calcd for C_38_H_36_FKN_3_NaO_3_^+^ 640.24, Found 640.15.

Butyl 1-cyclopropyl-6-fluoro-4-oxo-7-(4-tritylpiperazin-1-yl)-1,4-dihydroquinoline-3-carboxylate (**33**): Yellow solid (302 mg, 48%); R_f_ (PhMe/AcOΕt 1:1): 0.41; ^1^H NMR (600 MHz, CDCl_3_) *δ*: 8.48 (s, 1H), 7.96 (d, *J* = 13.4 Hz, 1H), 7.53 (br s, 5H), 7.29 (t, *J* = 7.6 Hz, 6H), 7.25–7.22 (m, 2H), 7.18 (t, *J* = 7.3 Hz, 3H), 4.31 (t, *J* = 6.8 Hz, 2H), 3.46–3.37 (s, 5H), 1.76 (q, *J* = 6.9 Hz, 3H), 1.52–1.45 (m, 3H), 1.34–1.30 (m, 2H), 1.27–1.25 (m, 1H), 1.15–1.10 (m, 2H), and 0.97 (t, *J* = 7.4 Hz, 3H); ^13^C NMR (150 MHz, CDCl_3_) *δ*: 173.2, 166.1, 154.2, 152.6, 148.1, 144.5, 138.1, 129.1, 128.3, 127.8, 126.4, 113.2, 110.5, 104.7, 64.8, 50.7, 47.8, 34.6, 31.0, 19.4, 13.9, and 8.3; and ESI-MS (30 eV) *m*/*z*: [M+H]^+^ Calcd for C_40_H_41_FN_3_O_3_^+^ 630.31, Found 630.38, [M+Νa]^+^ Calcd for C_40_H_40_FN_3_NaO_3_^+^ 652.29, Found 652.35, [M+Κ]^+^ Calcd for C_40_H_40_FKN_3_O_3_^+^ 668.27, Found 668.28. 

Ethyl 1-ethyl-6-fluoro-4-oxo-7-(4-tritylpiperazin-1-yl)-1,4-dihydroquinoline-3-carboxylate (**34**): Yellow solid (383 mg, 65%); R_f_ (PhMe/AcOΕt 1:1): 0.2; ^1^H NMR (600 MHz, CDCl_3_) *δ*: 8.40 (s, 1H), 8.03 (d, *J* = 13.4 Hz, 1H), 7.52 (br s, 5H), 7.32–7.23 (m, 6H), 7.21–7.13 (m, 3H), 6.73 (d, *J* = 6.5 Hz, 1H), 4.38 (q, *J* = 7.1 Hz, 2H), 4.20 (q, *J* = 6.5 Hz, 2H), 3.40 (br s, 4H), 2.35 (s, 1H), 2.17 (s, 1H), 1.66 (s, 2H), 1.54 (t, *J* = 6.8 Hz, 3H), and 1.40 (t, *J* = 6.8 Hz, 3H); ^13^C NMR (150 MHz, CDCl_3_) *δ*: 173.2, 166.2, 154.1, 148.1, 136.1, 129.2, 128.4, 127.8, 126.4, 125.4, 113.9, 113.7, 110.7, 103.7, 61.0, 50.8, 49.1, 47.8, 31.1, and 14.6; and ESI-MS (30 eV) *m*/*z*: [M+H]^+^ Calcd for C_37_H_37_FN_3_O_3_^+^ 590.28, Found 590.54, [M+Νa]^+^ Calcd for C_37_H_36_FN_3_NaO_3_^+^ 612.26, Found 612.44, [M+Κ]^+^ Calcd for C_37_H_36_FKN_3_O_3_^+^ 628.24, Found 628.49.

Butyl 1-ethyl-6-fluoro-4-oxo-7-(4-tritylpiperazin-1-yl)-1,4-dihydroquinoline-3-carboxylate (**35**): Yellow solid (277 mg, 45%); R_f_ (PhMe/AcOΕt 6:4): 0.26; ^1^H NMR (600 MHz, CDCl_3_) *δ*: 8.35 (s, 1H), 8.00 (d, *J* = 13.5 Hz, 1H), 7.52 (br s, 5H), 7.28 (t, *J* = 7.6 Hz, 7H), 7.18 (t, *J* = 7.0 Hz, 3H), 6.70 (d, *J* = 6.8 Hz, 1H), 4.31(q, *J* = 6.8 Hz, 2H), 4.18 (q, J = 7.2 Hz, 2H), 3.38 (br s, 4H), 2.34 (s, 1H), 2.16 (s, 1H), 2.04 (s, 1H), 1.81 (s, 1H), 1.79–1.73 (m, 2H), 1.52 (t, *J* = 7.3 Hz, 3H), 1.48 (q, J = 7.5 Hz, 2H), and 0.96 (t, *J* = 7.4 Hz, 3H); ^13^C NMR (150 MHz, CDCl_3_) *δ*: 173.2, 166.1, 154.0, 152.4, 148.0, 144.7, 136.2, 129.1 128.3, 127.8, 126.4, 113.7, 110.6, 103.6, 64.8, 50.7, 49.0, 47.8, 31.0, 19.4, 14.5, and 13.9; and ESI-MS (30 eV) *m*/*z*: [M+H]^+^ Calcd for C_39_H_41_FN_3_O_3_^+^ 618.31, Found 618.33, [M+Na]^+^ Calcd for C_39_H_40_FN_3_NaO_3_^+^ 640.29, Found 640.19.

#### 3.2.3. Synthesis of the Piperazine–Quinoline–CPX and Piperazine–Quinoline–NRX Conjugates **36** and **37**, Respectively

To an ice-cold solution of **30** or **31** (0.49 mmol) and 7-chloro-4-(piperazin-1-yl)-quinoline (**47**) (181 mg, 0.73 mmol) in DMF (1.9 mL), DIPEA (0.17 mL, 0.98 mmol) and TOTU (170 mg, 0.52 mmol) were added. After overnight stirring at room temperature, the reaction mixture was diluted with AcOEt; sequentially washed with water, 5% aqueous NaHCO_3_, water, and brine; dried over anhydrous Na_2_SO_4_; and concentrated to dryness under vacuum. The residues thus obtained were subjected to FCC purification affording the corresponding conjugates **36** and **37**.

3-(4-(7-chloroquinolin-4-yl)piperazine-1-carbonyl)-1-cyclopropyl-6-fluoro-7-(4-tritylpiperazin-1-yl)quinolin-4(1H)-one (**36**): Orange oil (240 mg, 61%); R_f_ (AcOΕt/MeOH 8:2): 0.25; ^1^H NMR (600 MHz, CDCl_3_) *δ*: 8.69 (d, *J* = 5.2 Hz, 1H), 8.13 (s, 1H), 8.08 (d, *J* = 2.0 Hz, 1H), 7.96 (d, *J* = 8.9 Hz, 1H), 7.93 (d, *J* = 13.3 Hz, 1H) 7.53 (br s, 5H), 7.43 (dd, *J* = 9.0, 2.2 Hz, 1H), 7.32–7.27 (m, 8H), 7.21–7.15 (m, 3H), 6.87 (d, *J* = 5.2 Hz, 1H), 4.08–4.03 (m, 2H), 3.73–3.67 (m, 2H), 3.48–3.40 (m, 4H), 3.38–3.32 (m, 5H), 1.33 (q, *J* = 6.9 Hz, 2H), and 1.17–1.13 (m, 2H); ^13^C NMR (150 MHz, CDCl_3_) *δ*: 172.5, 166.4, 154.3, 152.7, 145.4, 145.0, 144.9, 138.6, 129.5, 127.8, 127.1, 126.4, 125.5, 121.5, 116.9, 112.9, 112.8, 104.5, 66.8, 52.8, 52.2, 50.7, 47.8, 47.5, 42.5, 34.6, 29.8, 14.3, and 8.3; and ESI-MS (30 eV) *m*/*z*: [M+H]^+^ Calcd for C_49_H_45_ClFN_6_O_2_^+^ 803.33, Found 803.25, [M+Νa]^+^ Calcd for C_49_H_44_ClFN_6_NaO_2_^+^ 825.31, Found 825.16.

3-(4-(7-chloroquinolin-4-yl)piperazine-1-carbonyl)-1-ethyl-6-fluoro-7-(4-tritylpiperazin-1-yl)quinolin-4(1H)-one (**37**): Orange oil (236 mg, 61%); R_f_ (AcOΕt/MeOH 9:1): 0.30; ^1^H NMR (600 MHz, CDCl_3_) *δ*: 8.73–8.71 (m, 1H), 8.05–7.96 (m, 4H), 7.52 (br s, 5H), 7.46–7.42 (m, 1H), 7.32–7.27 (m, 7H), 7.22–7.14 (m, 3H), 6.90–6.86 (m, 1H), 6.79–6.74 (m, 1H), 4.26–4.17 (m, 2H), 4.06 (br s, 2H), 3.74–3.69 (m, 2H), 3.45–3.37 (m, 3H), 3.32 (br s, 4H), 1.80 (br s, 4H), and 1.60–1.48 (m, 4H); ^13^C NMR (150 MHz, CDCl_3_) *δ*: 172.4, 166.4, 156.9, 152.1, 150.2, 145.3, 136.6, 135.2, 129.5, 129.0, 127.8, 126.6, 126.4, 125.1, 123.9, 122.1, 117.3, 113.2, 109.4, 60.5, 52.9, 52.2, 50.8, 49.0, 47.8, 42.7, 29.8, 15.3, 14.6, and 14.3; and ESI-MS (30 eV) *m*/*z*: [M+H]^+^ Calcd for C_48_H_45_ClFN_6_O_2_^+^ 791.33, Found 791.41, [M+Na]^+^ Calcd for C_48_H_44_ClFN_6_NaO_2_^+^ 813.31, Found 813.43, [M+Κ]^+^ Calcd for C_49_H_45_ClFKN_6_O_2_^+^ 829.28, Found 829.47.

#### 3.2.4. Deprotection of Compounds **32**–**37**

To an ice-cold solution of the Trt-protected analogue or conjugate (0.05 mmol) in DCM (0.23 mL), anisole (10 μL, 0.09 mmol) and TFA (23 μL, 0.3 mmol) were added, and the reaction mixture was stirred for 15 min at 0 °C and then for 45 min at room temperature. Then, a mixture of Et_2_O and *n*-hexane was added dropwise until full precipitation of the corresponding tris-trifluoroacetate salt, which was collected upon filtration under vacuum and used in the next step without further purification.

#### 3.2.5. General Procedure for the Synthesis of the Hybrids **12**–**14**, **20**, and **21**

To a stirred solution of **44**, **45**, or **46** (0.25 mmol) in anhydrous DCM (6.1 mL), BOP (84 mg, 0.19 mmol) was added, the mixture was cooled to 0 °C and the addition of DIPEA (80 μL, 0.46 mmol) was undertaken. After 40 min, CPX or CPX analogue (0.25 mmol) was added, and the reaction mixture was left under overnight stirring at room temperature. Upon completion of the reaction, monitored with the TLC, the mixture was diluted with DCM, and the organic layer was washed sequentially with cold 5% aqueous citric acid, water, and brine. After being dried over anhydrous Na_2_SO_4_, the organic extracts were filtered and evaporated to dryness under reduced pressure. The obtained residues were subjected to FCC to provide the pure hybrids as yellow solids.

1-cyclopropyl-6-fluoro-4-oxo-7-(4-(2-((3R,6R,9R,10R,12R,12aR)-3,6,9-trimethyldecahydro-12H-3,12-epoxy[1,2]dioxepino[4,3-i]isochromen-10-yl)acetyl)piperazin-1-yl)-1,4-dihydroquinoline-3-carboxylic acid (**12**): Yellow solid (82 mg, 51%); mp: 123–124 °C; R_f_ (AcOΕt/MeOH 93:7): 0.31; ^1^H NMR (600 MHz, CDCl_3_) *δ*: 14.92 (s, 1H), 8.77 (s, 1H), 8.04 (d, *J* = 12.8 Hz, 1H), 7.36 (d, *J* = 6.8 Hz, 1H), 5.33 (s, 1H), 4.90–4.86 (m, 1H), 3.87–3.81 (m, 1H), 3.72–3.65 (m, 2H), 3.55–3.51 (m, 1H), 3.47–3.41 (m, 1H), 3.37–3.33 (m, 2H), 3.24–3.19 (m, 1H), 2.79–2.70 (m, 2H), 2.49 (dd, *J* = 14.6, 4.4 Hz, 1H), 2.35–2.28 (m, 2H), 1.96–1.91 (m, 3H), 1.81 (dd, *J* = 13.8, 4.0 Hz, 2H), 1.39 (d, *J* = 6.8 Hz, 3H), 1.37 (s, 3H), 1.22–1.19 (m, 6H), 0.96 (d, *J* = 5.9 Hz, 3H), and 0.91 (d, *J* = 7.5 Hz, 3H); ^13^C NMR (150 MHz, CDCl_3_) *δ*: 177.2, 170.1, 167.0, 149.9, 147.7, 139.2, 128.4, 112.7, 108.2, 103.0, 96.0, 90.0, 81.1, 71.3, 64.5, 52.1, 44.0, 43.1, 37.7, 36.6, 35.3, 34.5, 30.8, 30.4, 29.8, 26.1, 24.9, 22.6, 20.2, 19.3, 16.2, 13.8, and 8.4; ^19^F NMR (564 MHz, CDCl_3_) *δ*: −121.1; and ESI-MS (30 eV) *m*/*z*: [M+H]^+^ Calcd for C_34_H_43_FN_3_O_8_^+^ 640.30, Found 640.50, [M+Na]^+^ Calcd for C_34_H_42_FN_3_NaO_8_^+^ 662.28, Found 662.48, [M+Κ]^+^ Calcd for C_34_H_42_FKN_3_O_8_^+^ 678.26, Found 678.54.

1-cyclopropyl-6-fluoro-4-oxo-7-(4-(3-((3R,6R,9R,10R,12R,12aR)-3,6,9-trimethyldecahydro-12H-3,12-epoxy[1,2]dioxepino[4,3-i]isochromen-10-yl)propanoyl)piperazin-1-yl)-1,4-dihydroquinoline-3-carboxylic acid (**13**): Yellow solid (90 mg, 55%); mp: 136–137 °C; R_f_ (AcOΕt/MeOH 95:5): 0.25; ^1^H NMR (600 MHz, CDCl_3_) *δ*: 8.80 (s, 1H), 8.07 (d, *J* = 12.6 Hz, 1H), 7.38 (d, *J* = 7.0 Hz, 1H), 5.30 (s, 1H), 4.10–4.06 (m, 1H), 3.98–3.69 (m, 4H), 3.58–3.52 (m, 1H), 3.41–3.32 (m, 4H), 2.80–2.71 (m, 2H), 2.47–2.39 (m, 1H), 2.37–2.31 (m, 2H), 2.05–1.98 (m, 3H), 1.93–1.88 (m, 4H), 1.66 (dd, *J* = 12.1, 3.4 Hz, 1H), 1.57 (dt, *J* = 13.2, 4.7 Hz, 1H), 1.44–1.38 (m, 6H), 1.23–1.18 (m, 4), 0.96 (d, *J* = 6.1 Hz, 3H), and 0.91 (d, *J* = 7.6 Hz, 3H); ^13^C NMR (150 MHz, CDCl_3_) *δ*: 177.3, 171.9, 167.1, 152.9, 147.8, 139.2, 128.1, 120.5, 113.0, 108.5, 105.2, 103.6, 88.9, 81.3, 76.4, 52.7, 49.7, 45.4, 44.7, 41.5, 37.6, 36.7, 35.5, 34.6, 31.0, 30.3, 26.4, 25.0, 24.8, 24.7, 22.8, 20.4, 14.3, 13.5, and 8.4; ^19^F NMR (564 MHz, CDCl_3_) *δ*: −121.1; and ESI-MS (30 eV) *m*/*z*: [M+H]^+^ Calcd for C_35_H_45_FN_3_O_8_^+^ 654.32, Found 654.61, [M+Na]^+^ Calcd for C_35_H_44_FN_3_NaO_8_^+^ 676.30, Found 676.40, [M+Κ]^+^ Calcd for C_35_H_44_FKN_3_O_8_^+^ 692.27, Found 692.52.

Ethyl 1-cyclopropyl-6-fluoro-4-oxo-7-(4-(2-((3R,6R,9R,10R,12R,12aR)-3,6,9-trimethyldecahydro-12H-3,12-epoxy[1,2]dioxepino[4,3-i]isochromen-10-yl)acetyl)piperazin-1-yl)-1,4-dihydroquinoline-3-carboxylate (**14**): Yellow solid (119 mg, 71%); mp: 142–143 °C; R_f_ (PhMe/AcOEt 1:9): 0.33; ^1^H NMR (600 MHz, CDCl_3_) *δ*: 8.52 (s, 1H), 8.06 (d, *J* = 13.1 Hz, 1H), 7.27 (s, 1H), 5.33 (s, 1H), 4.91–4.85 (m, 1H), 4.38 (q, *J* = 7.1 Hz, 2H), 4.08–4.03 (m, 1H), 3.86–3.76 (m, 2H), 3.71–3.63 (m, 2H), 3.41 (septet, *J* = 3.2 Hz, 1H), 3.30–3.24 (m, 2H), 3.19–3.13 (m, 1H), 2.81–2.71 (m, 2H), 2.49 (dd, *J* = 14.6, 4.6 Hz, 1H), 2.34–2.28 (m, 1H), 2.03–2.00 (m, 1H), 1.96–1.91 (m, 1H), 1.81 (dd, *J* = 13.4, 3.7 Hz, 1H), 1.73–1.66 (m, 3H), 1.40 (t, *J* = 7.1 Hz, 3H), 1.38 (s, 3H), 1.32 (d, *J* = 6.5 Hz, 2H), 1.28–1.25 (m, 4H), 1.15–1.11 (m, 2H), 0.96 (d, *J* = 6.0 Hz, 3H), and 0.91 (d, *J* = 7.5 Hz, 3H); ^13^C NMR (150 MHz, CDCl_3_) *δ*: 173.3, 170.1, 166.0, 148.4, 144.3, 138.2, 128.1, 119.8, 110.7, 105.2, 103.1, 89.9, 81.1, 71.3, 61.1, 52.1, 44.0, 37.7, 36.7, 35.3, 34.7, 30.4, 26.2, 24.9, 21.2, 20.2, 14.6, 14.3, 12.7, and 8.3; ^19^F NMR (564 MHz, CDCl_3_) *δ*: −120.8; and ESI-MS (30 eV) *m*/*z*: [M+H]^+^ Calcd for C_36_H_47_FN_3_O_8_^+^ 668.33, Found 668.51, [M+Na]^+^ Calcd for C_36_H_46_FN_3_NaO_8_^+^ 690.32, Found 690.47, [M+Κ]^+^ Calcd for C_36_H_46_FKN_3_O_8_^+^ 706.29, Found 706.39.

1-cyclopropyl-6-fluoro-4-oxo-7-(4-(4-oxo-4-(((3R,6R,9R,10S,12R,12aR)-3,6,9-trimethyldecahydro-12H-3,12-epoxy[1,2]dioxepino[4,3-i]isochromen-10-yl)oxy)butanoyl)piperazin-1-yl)-1,4-dihydroquinoline-3-carboxylic acid (**20**): Yellow solid (70 mg, 40%); mp: 103.4–104.5 °C; R_f_ (AcOΕt/MeOH 97:3): 0.25; ^1^H NMR (600 MHz, CDCl_3_) *δ*: 8.76 (s, 1H), 8.02 (d, *J* = 12.8 Hz, 1H), 7.39 (d, *J* = 7.0 Hz, 1H), 5.79 (d, *J* = 9.8 Hz, 1H), 5.42 (s, 1H), 3.90–3.84 (m, 2H), 3.79–3.74 (m, 2H), 3.56 (septet, *J* = 7.2 Hz, 1H), 3.41–3.36 (m, 2H), 3.33–3.28 (m, 2H), 2.89–2.82 (m, 1H), 2.80–2.77 (m, 1H), 2.77–2.72 (m, 1H), 2.68–2.62 (m, 1H), 2.60–2.53 (m, 1H), 2.38–2.32 (m, 1H), 2.04–2.00 (m, 2H), 1.91–1.86 (m, 1H), 1.80–1.75 (m, 1H), 1.73–1.69 (m, 1H), 1.61 (dt, *J* = 13.8, 4.6 Hz, 1H), 1.50–1.44 (m, 1H), 1.41(s, 3H), 1.36 (dd, *J* = 13.6, 3.5 Hz, 1H), 1.33–1.24 (m, 3H), 1.22–1.20 (m, 2H), 1.04–0.99 (m, 1H), 0.96 (d, *J* = 6.1 Hz, 3H), and 0.87 (d, *J* = 7.1 Hz, 3H); ^13^C NMR (150 MHz, CDCl_3_) *δ*: 177.2, 171.9, 169.9, 167.0, 154.6, 152.9, 147.7, 139.2, 112.7, 108.4, 105.4, 104.6, 92.3, 91.7, 80.3, 60.5, 51.7, 45.4, 41.5, 37.4, 36.4, 35.5, 34.2, 32.0, 29.6, 27.7, 26.1, 24.7, 22.1, 20.3, 14.3, 12.2, and 8.4; ^19^F NMR (564 MHz, CDCl_3_) *δ*: −121.1; and ESI-MS (30 eV) *m*/*z*: [M+H]^+^ Calcd for C_36_H_45_FN_3_O_10_^+^ 698.31, Found 698.84, [M+Na]^+^ Calcd for C_36_H_44_FN_3_NaO_10_^+^ 720.29, Found 720.43, [M+Κ]^+^ Calcd for C_36_H_44_FKN_3_O_10_^+^ 736.26, Found 736.76.

Ethyl 1-cyclopropyl-6-fluoro-4-oxo-7-(4-(4-oxo-4-(((3R,6R,9R,10S,12R,12aR)-3,6,9-trimethyldecahydro-12H-3,12-epoxy[1,2]dioxepino[4,3-i]isochromen-10-yl)oxy)butanoyl)piperazin-1-yl)-1,4-dihydroquinoline-3-carboxylate (**21**): Yellow solid (151 mg, 83%); mp: 115.4–116.2 °C; R_f_ (PhMe/AcOΕt 1:9): 0.20; ^1^H NMR (600 MHz, CDCl_3_) *δ*: 8.53 (s, 1H), 8.06 (d, *J* = 13.0 Hz, 1H), 7.28 (d, *J* = 7.0 Hz, 1H), 5.79 (d, *J* = 9.8 Hz, 1H), 5.43 (s, 1H), 4.38 (q, *J* = 7.1 Hz, 2H), 3.87–3.82 (m, 2H), 3.75–3.71 (m, 2H), 3.44 (septet, *J* = 3.0 Hz, 1H), 3.32–3.26 (m, 2H), 3.24–3.19 (m, 2H), 2.88–2.82 (m, 1H), 2.80–2.71 (m, 3H), 2.69–2.62 (m, 1H), 2.59–2.54 (m, 1H), 2.39–2.33 (m, 2H), 2.05–2.00 (m, 2H), 1.91–1.86 (m, 1H), 1.80–1.74 (m, 1H), 1.73–1.69 (m, 1H), 1.61 (dt, *J* = 13.8, 4.6 Hz, 1H), 1.50–1.45 (m, 1H), 1.42–1.38 (m, 6H), 1.32 (d, *J* = 6.7 Hz, 2H), 1.15–1.11 (m, 2H), 1.04–0.99 (m, 1H), 0.96 (d, *J* = 6.1 Hz, 3H), and 0.87 (d, *J* = 7.1 Hz, 3H); ^13^C NMR (150 MHz, CDCl_3_) *δ*: 173.2, 171.9, 169.9, 166.0, 152.7, 148.4, 144.2, 138.2, 128.4, 113.6, 110.7, 105.3, 104.6, 92.3, 91.7, 80.3, 61.1, 51.7, 50.5, 49.8, 45.4, 41.7, 37.4, 36.4, 34.7, 34.2, 32.0, 31.9, 29.6, 27.7, 26.1, 24.7, 22.1, 20.3, 14.6, 12.2, and 8.3; ^19^F NMR (564 MHz, CDCl_3_) *δ*: −120.8; and ESI-MS (30 eV) *m*/*z*: [M+H]^+^ Calcd for C_38_H_49_FN_3_O_10_^+^ 726.34, Found 726.47, [M+Na]^+^ Calcd for C_38_H_48_FN_3_NaO_10_^+^ 748.32, Found 748.58, [M+Κ]^+^ Calcd for C_38_H_48_FKN_3_O_10_^+^ 764.30, Found 764.45.

#### 3.2.6. General Procedure for the Synthesis of the Hybrids **15**–**19** and **22**–**29**

To an ice-cold solution of **44**, **45**, or **46** (0.15 mmol) and the drug analogue or the drug conjugate (0.15 mmol) in DMF (0.45 mL), DIPEA (0.1 mL, 0.6 mmol) and TOTU (74 mg, 0.225 mmol) were added. The reaction mixture was stirred for 10 min at 0 °C and then at room temperature. Upon completion of the reaction, the mixture was diluted with DCM, and the organic layer was washed sequentially with water, 5% aqueous NaHCO_3_, water, and brine; dried over anhydrous Na_2_SO_4_; filtered; and evaporated to dryness under vacuum. The crude residues were subjected to FCC to give the pure products as yellow solids.

Ethyl 1-cyclopropyl-6-fluoro-4-oxo-7-(4-(3-((3R,6R,9R,10R,12R,12aR)-3,6,9-trimethyldecahydro-12H-3,12-epoxy[1,2]dioxepino[4,3-i]isochromen-10-yl)propanoyl)piperazin-1-yl)-1,4-dihydroquinoline-3-carboxylate (**15**): Reaction time: 2 h; yellow solid (61 mg, 60%); mp: 110.4–111.5 °C; R_f_ (AcOΕt/MeOH 9:1): 0.24; ^1^H NMR (600 MHz, CDCl_3_) *δ*: 8.52 (s, 1H), 8.05 (d, *J* = 13.0 Hz, 1H), 7.25 (s, 1H), 5.30 (s, 1H), 4.38 (q, *J* = 7.1 Hz, 2H), 4.11–4.06 (m, 1H), 3.91–3.85 (m, 1H), 3.84–3.79 (m, 1H), 3.77–3.71 (m, 2H), 3.43–3.39 (m, 1H), 3.31–3.19 (m, 4H), 2.80–2.70 (m, 2H), 2.45–2.38 (m, 1H), 2.38–2.29 (m, 1H), 2.04–1.98 (m, 2H), 1.91–1.80 (m, 3H), 1.65 (dd, *J* = 13.2, 3.1 Hz, 1H), 1.57 (dt, *J* = 13.8, 5.0 Hz, 1H), 1.50–1.42 (m, 2H), 1.41–1.38 (m, 6H), 1.32 (d, *J* = 6.7 Hz, 2H), 1.28–1.24 (m, 2H), 1.21 (t, *J* = 7.1 Hz, 1H), 1.15–1.12 (m, 2H), 0.95 (d, *J* = 6.2 Hz, 3H), and 0.91 (d, *J* = 7.5 Hz, 3H); ^13^C NMR (150 MHz, CDCl_3_) *δ*: 173.2, 171.7, 166.0, 154.3, 152.7, 148.4, 144.3, 138.1, 129.2, 123.7, 113.6, 110.8, 105.1, 103.6, 88.8, 81.3, 76.4, 61.1, 52.6, 50.5, 49.8, 45.5, 44.7, 41.5, 37.6, 36.7, 34.6, 31.1, 30.3, 26.4, 25.0, 24.8, 24.7, 20.4, 14.6, 13.5, and 8.3; ^19^F NMR (564 MHz, CDCl_3_) *δ*: −123.3; and ESI-MS (30 eV) *m*/*z*: [M+H]^+^ Calcd for C_37_H_49_FN_3_O_8_^+^ 682.35, Found 682.13, [M+Na]^+^ Calcd for C_37_H_48_FN_3_NaO_8_^+^ 704.33, Found 704.24.

Butyl 1-cyclopropyl-6-fluoro-4-oxo-7-(4-(2-((3R,6R,9R,10R,12R,12aR)-3,6,9-trimethyldecahydro-12H-3,12-epoxy[1,2]dioxepino[4,3-i]isochromen-10-yl)acetyl)piperazin-1-yl)-1,4-dihydroquinoline-3-carboxylate (**16**): Reaction time: 2 h; yellow solid (50 mg, 48%); mp: 105–106 °C; R_f_ (PhMe/AcOΕt 2:8): 0.20; ^1^H NMR (600 MHz, CDCl_3_) *δ*: 8.50 (s, 1H), 8.05 (d, *J* = 13.1 Hz, 1H), 7.25 (s, 1H), 5.33 (s, 1H), 4.9–4.86 (m, 1H), 4.31 (t, *J* = 6.8 Hz, 2H), 4.09–4.03 (m, 1H), 3.84–3.77 (m, 1H), 3.71–3.62 (m, 2H), 3.40 (septet, *J* = 3.4 Hz, 1H), 3.38–3.33 (m, 1H), 3.30–3.24 (m, 2H), 3.18–3.12 (m, 1H), 2.80–2.69 (m, 2H), 2.49 (dd, *J* = 14.6, 4.6 Hz, 1H), 2.31 (td, *J* = 14.4, 3.9 Hz, 1H), 2.02–1.98 (m, 1H), 1.95–1.90 (m, 1H), 1.84–1.64 (m, 6H), 1.48 (sextet, *J* = 15.0, 7.4 Hz, 2H), 1.37 (s, 3H), 1.33–1.22 (m, 6H), 1.15–1.10 (m, 2H), 0.98–0.94 (m, 6H), and 0.91 (d, *J* = 7.6 Hz, 3H); ^13^C NMR (150 MHz, CDCl_3_) *δ*: 173.2, 170.1, 166.0, 154.3, 152.7, 148.3, 144.3, 138.1, 123.7, 113.7, 113.6, 110.8, 105.2, 103.0, 89.9, 81.0, 71.3, 64.9, 60.5, 52.1, 50.8, 49.7, 46.1, 44.0, 41.6, 37.7, 36.7, 35.3, 34.6, 31.0, 30.4, 26.1, 24.9, 20.2, 19.4, 14.3, 14.0, 12.7, and 8.3; ^19^F NMR (564 MHz, CDCl_3_) *δ*: −122.8; and ESI-MS (30 eV) *m*/*z*: [M+H]^+^ Calcd for C_38_H_51_FN_3_O_8_^+^ 696.37, Found 696.56, [M+Na]^+^ Calcd for C_38_H_50_FN_3_NaO_8_^+^ 718.35, Found 718.54, [M+K]^+^ Calcd for C_38_H_50_FKN_3_O_8_^+^ 734.32, Found 734.35.

Butyl 1-cyclopropyl-6-fluoro-4-oxo-7-(4-(3-((3R,6R,9R,10R,12R,12aR)-3,6,9-trimethyldecahydro-12H-3,12-epoxy[1,2]dioxepino[4,3-i]isochromen-10-yl)propanoyl)piperazin-1-yl)-1,4-dihydroquinoline-3-carboxylate (**17**): Reaction time: 2 h; yellow solid (64 mg, 60%); mp: 118.5–119.5 °C; R_f_ (PhMe/AcOΕt 3:7): 0.21; ^1^H NMR (600 MHz, CDCl_3_) *δ*: 8.52 (s, 1H), 8.07 (d, *J* = 13.1 Hz, 1H), 7.48 (d, *J* = 7.5 Hz, 1H), 5.30 (s, 1H), 4.33 (t, *J* = 6.7 Hz, 2H), 4.12–4.06 (m, 1H), 3.93–3.69 (m, 4H), 3.42 (br s, 1H), 3.33–3.20 (m, 4H), 2.80–2.72 (m, 2H), 2.45–2.31 (m, 2H), 2.05–1.99 (m, 2H), 1.92–1.82 (m, 3H), 1.77 (q, *J* = 7.2 Hz, 2H), 1.62–1.57 (m, 4H), 1.66 (dd, *J* = 13.6, 3.1 Hz, 1H), 1.48 (sextet, *J* = 7.4 Hz, 2H), 1.45–1.41 (m, 1H), 1.40 (s, 3H), 1.33 (d, *J* = 6.4 Hz, 2H), 1.28–1.24 (m, 1H), 1.13 (br s, 2H), 0.99–0.94 (m, 6H), and 0.91 (d, *J* = 7.5 Hz, 3H); ^13^C NMR (150 MHz, CDCl_3_) *δ*: 175.8, 171.7, 166.1, 148.3, 145.2, 138.2, 128.1, 119.7, 113.8, 105.2, 103.6, 88.8, 81.3, 76.4, 65.0, 52.7, 49.9, 45.5, 44.7, 41.6, 37.6, 36.7, 34.6, 31.1, 31.0, 30.3, 26.4, 25.0, 24.8, 24.7, 20.4, 19.4, 14.0, 13.5, and 8.3; ^19^F NMR (564 MHz, CDCl_3_) *δ*: −123.0; and ESI-MS (30 eV) *m*/*z*: [M+H]^+^ Calcd for C_39_H_53_FN_3_O_8_^+^ 710.38, Found 710.36, [M+Na]^+^ Calcd for C_39_H_52_FN_3_NaO_8_^+^ 732.36, Found 732.45, [M+K]^+^ Calcd for C_39_H_52_FKN_3_O_8_^+^ 748.34, Found 748.25.

Ethyl 1-ethyl-6-fluoro-4-oxo-7-(4-(2-((3R,6R,9R,10R,12R,12aR)-3,6,9-trimethyldecahydro-12H-3,12-epoxy[1,2]dioxepino[4,3-i]isochromen-10-yl)acetyl)piperazin-1-yl)-1,4-dihydroquinoline-3-carboxylate (**18**): Reaction time: 12 h; yellow solid (59 mg, 60%); mp: 135.1–136 °C; R_f_ (AcOΕt/MeOH 95:5): 0.34; ^1^H NMR (600 MHz, CDCl_3_) *δ*: 8.41 (s, 1H), 8.09 (d, *J* = 13.1 Hz, 1H), 6.74 (d, *J* = 6.7 Hz, 1H), 5.33 (s, 1H), 4.89–4.84 (m, 1H), 4.38 (q, *J* = 7.1 Hz, 2H), 4.19 (q, *J* = 7.1 Hz, 2H), 4.08–4.02 (m, 1H), 3.86 (t, *J* = 5.2 Hz, 1H), 3.66 (t, *J* = 5.4 Hz, 2H), 3.37–3.31 (m, 1H), 3.29–3.22 (m, 2H), 3.16–3.08 (m, 1H), 2.81–2.69 (m, 2H), 2.48 (dd, *J* = 14.7, 4.5 Hz, 1H), 2.32–2.28 (m, 1H), 2.00 (dt, *J* = 13.9, 4.0 Hz, 1H), 1.95–1.90 (m, 1H), 1.83–1.78 (m, 1H), 1.72–1.65 (m, 2H), 1.53 (t, *J* = 7.2 Hz, 3H), 1.40 (t, *J* = 7.1 Hz, 3H), 1.37 (s, 3H), 1.29–1.24 (m, 4H), 1.06 (s, 1H), 0.96 (d, *J* = 5.9 Hz, 3H), and 0.91 (d, *J* = 7.5 Hz, 3H); ^13^C NMR (150 MHz, CDCl_3_) *δ*: 173.2, 170.1, 166.0, 152.5, 148.3, 144.7, 136.3, 134.1, 123.4, 114.1, 110.7, 104.3, 103.1, 89.9, 81.0, 71.4, 63.1, 61.1, 52.1, 49.8, 47.0, 46.1, 44.0, 41.6, 39.6, 37.7, 36.6, 35.2, 34.5, 30.4, 26.1, 24.9, 20.2, 14.6, and 12.7; ^19^F NMR (564 MHz, CDCl_3_) *δ*: −122.7; and ESI-MS (30 eV) *m*/*z*: [M+H]^+^ Calcd for C_35_H_47_FN_3_O_8_^+^ 656.33, Found 656.31, [M+Na]^+^ Calcd for C_35_H_46_FN_3_NaO_8_^+^ 678.32, Found 678.23, [M+K]^+^ Calcd for C_35_H_46_FKN_3_O_8_^+^ 694.29, Found 694.29.

Butyl 1-ethyl-6-fluoro-4-oxo-7-(4-(2-((3R,6R,9R,10R,12R,12aR)-3,6,9-trimethyldecahydro-12H-3,12-epoxy[1,2]dioxepino[4,3-i]isochromen-10-yl)acetyl)piperazin-1-yl)-1,4-dihydroquinoline-3-carboxylate (**19**): Reaction time: 2 h; yellow solid (32 mg, 31%); mp: 151–152 °C; R_f_ (AcOΕt/MeOH 97:3): 0.36; ^1^H NMR (600 MHz, CDCl_3_) *δ*: 8.41 (s, 1H), 8.12 (d, *J* = 13.0 Hz, 1H), 6.73 (d, *J* = 6.5 Hz, 1H), 5.33 (s, 1H), 4.91–4.83 (m, 1H), 4.33 (t, *J* = 6.8 Hz, 2H), 4.22–4.19 (m, 2H), 3.86–3.75 (m, 2H), 3.69–3.63 (m, 2H), 3.37–3.10 (m, 4H), 3.17–3.08 (m, 1H), 2.81–2.71 (m, 2H), 2.49 (dd, *J* =14.8, 4.6 Hz, 1H), 2.36–2.29 (m, 2H), 2.03–1.98 (m, 1H), 1.96–1.91 (m, 1H), 1.77 (q, *J* = 7.2 Hz, 3H), 1.70–1.65 (m, 1H), 1.53 (t, *J* = 7.2 Hz, 3H), 1.49 (q, *J* = 7.6 Hz, 2H), 1.38 (s, 3H), 1.28 (d, *J* = 4.6 Hz, 2H), 1.04 (dd, *J* = 15.9, 6.2 Hz, 2H), 0.98–0.95 (m, 6H), and 0.91 (d, *J* = 7.5 Hz, 3H); ^13^C NMR (150 MHz, CDCl_3_) *δ*: 177.8, 174.8, 170.2, 160.5, 155.6, 148.3, 146.5, 128.7, 124.4, 113.9, 109.7, 103.1, 98.9, 89.9, 81.1, 71.4, 52.1, 50.7, 49.9, 46.1, 44.0, 41.6, 40.4, 37.7, 36.7, 35.2, 34.5, 30.9, 30.4, 26.2, 24.9, 20.2, 19.4, 14.6, 13.9, and 12.7; ^19^F NMR (564 MHz, CDCl_3_) *δ*: −120.3; and ESI-MS (30 eV) *m/z:* [M+H]^+^ Calcd for C_37_H_51_FN_3_O_8_^+^ 684.37, Found 684.36, [M+Na]^+^ Calcd for C_37_H_50_FN_3_NaO_8_^+^ 706.35, Found 706.40, [M+K]^+^ Calcd for C_37_H_50_FKN_3_O_8_^+^ 722.32, Found 722.38.

Butyl 1-cyclopropyl-6-fluoro-4-oxo-7-(4-(4-oxo-4-(((3R,6R,9R,10S,12R,12aR)-3,6,9-trimethyldecahydro-12H-3,12-epoxy[1,2]dioxepino[4,3-i]isochromen-10-yl)oxy)butanoyl)piperazin-1-yl)-1,4-dihydroquinoline-3-carboxylate (**22**): Reaction time: 2 h; yellow solid (63 mg, 56%); mp: 128.6–129.7 °C; R_f_ (PhMe/AcOΕt 2:8): 0.20; ^1^H NMR (600 MHz, CDCl_3_) *δ*: 8.53 (s, 1H), 8.03 (d, *J* = 12.9 Hz, 1H), 7.31 (d, *J* = 7.1 Hz, 1H), 5.79 (d, *J* = 9.8 Hz, 1H), 5.43 (s, 1H), 4.33 (t, *J* = 6.7 Hz, 2H), 3.87–3.82 (m, 2H), 3.76–3.72 (m, 2H), 3.36–329 (m, 2H), 3.27–3.21 (m, 2H), 2.88–2.82 (m, 1H), 2.80–2.64 (m, 4H), 2.59–2.54 (m, 1H), 2.39–2.33 (m, 1H), 2.04–2.00 (m, 1H), 1.91–1.86 (m, 1H), 1.79–1.74 (m, 3H), 1.73–1.69 (m, 1H), 1.62 (dt, *J* = 13.7, 4.5 Hz, 1H), 1.47 (q, *J* = 7.6 Hz, 3H), 1.42 (s, 3H), 1.36–1.33 (m, 2H), 1.30–1.26 (m, 4H), 0.97 (s, 2H), 0.98–0.94 (m, 6H), and 0.87 (d, *J* = 7.1 Hz, 3H); ^13^C NMR (150 MHz, CDCl_3_) *δ*: 173.2, 171.9, 169.8, 166.1, 153.0, 148.3, 144.2, 138.2, 129.2, 123.1, 113.6, 105.2, 104.6, 92.3, 91.7, 80.3, 74.7 65.0, 51.7, 45.4, 41.7, 37.4, 36.4, 34.6, 34.2, 32.0, 31.0, 29.6, 27.7, 26.1, 24.7, 22.2, 20.3, 19.4, 14.0, 12.2, and 8.3; ^19^F NMR (564 MHz, CDCl_3_) *δ*: −123.0; and ESI-MS (30 eV) *m/z:* [M+H]^+^ Calcd for C_40_H_53_FN_3_O_10_^+^ 754.37, Found 754.44, [M+Na]^+^ Calcd for C_40_H_52_FN_3_NaO_10_^+^ 776.35, Found 776.42, [M+K]^+^ Calcd for C_40_H_52_FKN_3_O_10_^+^ 792.33, Found 792.48.

Ethyl 1-ethyl-6-fluoro-4-oxo-7-(4-(4-oxo-4-(((3R,6R,9R,10S,12R,12aR)-3,6,9-trimethyldecahydro-12H-3,12-epoxy[1,2]dioxepino[4,3-i]isochromen-10-yl)oxy)butanoyl)piperazin-1-yl)-1,4-dihydroquinoline-3-carboxylate (**23**): Reaction time: 12 h; yellow solid (94 mg, 88%); mp: 140–141.1 °C; R_f_ (AcOΕt/MeOH 9:1): 0.31; ^1^H NMR (600 MHz, CDCl_3_) *δ*: 8.43 (s, 1H), 8.11(d, *J* = 13.0 Hz, 1H), 6.74 (d, *J* = 6.6 Hz, 1H), 5.79 (d, *J* = 9.8 Hz, 1H), 5.43 (s, 1H,), 4.39 (q, *J* = 7.1 Hz, 2H), 4.20 (q, *J* = 7.1 Hz, 2H), 3.84 (t, *J* = 4.8 Hz, 2H), 3.73 (t, *J* = 4.8 Hz, 2H), 3.30–3.24 (m, 2H), 3.23–3.17 (m, 2H), 2.88–2.82 (m, 1H), 2.80–2.72 (m, 2H), 2.67–2.62 (m, 1H), 2.60–2.54 (m, 1H), 2.35 (td, *J* = 14.2, 3.8 Hz, 1H), 2.05–1.99 (m, 1H), 1.91–1.86 (m, 1H), 1.80–1.76 (m, 2H), 1.73–1.69 (m, 1H), 1.62 (dt, *J* = 13.7, 4.2 Hz, 1H), 1.54 (t, *J* = 7.3 Hz, 3H), 1.51–1.45 (m, 1H), 1.43–1.39 (s, 6H), 1.37 (dd, *J* =13.6, 3.2 Hz, 1H), 1.29–1.24 (m, 1H), 1.05–0.99 (m, 1H), 0.96 (d, *J* = 6.1 Hz, 3H), and 0.87 (d, *J* = 7.1 Hz, 3H); ^13^C NMR (150 MHz, CDCl_3_) *δ*: 173.2, 171.9, 169.8, 166.1, 154.2, 152.5, 148.3, 144.5, 136.2, 114.1, 110.8, 104.6, 104.3, 92.3, 91.7, 80.3, 61.1, 51.7, 50.5, 49.9, 49.1, 45.4, 41.7, 37.4, 36.4, 34.2, 32.0, 29.9, 27.7, 26.1, 24.7, 22.1, 20.3, 14.6, and 12.2; ^19^F NMR (564 MHz, CDCl_3_) *δ*: −123.1; and ESI-MS (30 eV) *m/z:* [M+H]^+^ Calcd for C_37_H_49_FN_3_O_10_^+^ 714.34, Found 714.25, [M+Na]^+^ Calcd for C_37_H_48_FN_3_NaO_10_^+^ 736.79, Found 736.24, [M+K]^+^ Calcd for C_37_H_48_FKN_3_O_10_^+^ 752.30, Found 752.17.

Butyl 1-ethyl-6-fluoro-4-oxo-7-(4-(4-oxo-4-(((3R,6R,9R,10S,12R,12aR)-3,6,9-trimethyldecahydro-12H-3,12-epoxy[1,2]dioxepino[4,3-i]isochromen-10-yl)oxy)butanoyl)piperazin-1-yl)-1,4-dihydroquinoline-3-carboxylate (**24**): Reaction time: 12 h; yellow solid (67 mg, 60%); mp: 155–156 °C; R_f_ (AcOΕt/MeOH 99:1): 023; ^1^H NMR (600 MHz, CDCl_3_) *δ*: 8.49 (s, 1H), 8.12 (d, *J* = 13.0 Hz, 1H), 6.80 (d, *J* = 6.2 Hz, 1H), 5.79 (d, *J* = 9.8 Hz, 1H), 5.43 (s, 1H), 4.34 (t, *J* = 6.8 Hz, 2H), 4.26 (d, *J* = 6.9 Hz, 2H), 3.84 (t, *J* = 5.6 Hz, 2H), 3.77–3.70 (m, 2H), 3.33–3.29 (m, 2H), 3.25–3.20 (m, 2H), 2.88–2.82 (m, 1H), 2.80–2.72 (m, 2H), 2.68–2.62 (m, 1H), 2.60–2.54 (m, 1H), 2.39–2.33 (m, 1H), 2.04–2.00 (m, 1H), 1.91–1.86 (m, 1H), 1.80–1.75 (m, 4H), 1.73–1.69 (m, 2H), 1.62 (dt, *J* = 13.7, 4.4 Hz, 1H), 1.55 (t, *J* = 6.8 Hz, 3H), 1.51–1.47 (m, 2H), 1.42 (s, 3H), 1.37 (dd, *J* = 13.6, 3.5 Hz, 1H), 1.29–1.27 (m, 1H), 1.05–1.01 (m, 1H), 0.99–0.95 (m, 6H), and 0.87 (d, *J* = 7.1 Hz, 3H); ^13^C NMR (150 MHz, CDCl_3_) *δ*: 171.9, 169.9, 166.3, 152.6, 148.3, 142.1, 114.1, 104.6, 104.4, 92.3, 91.7, 80.3, 65.2, 51.7, 50.5, 49.9, 45.4, 41.7, 38.8, 37.4, 36.4, 34.2, 32.0, 31.1, 31.0, 29.6, 27.7, 26.1, 24.7, 22.2, 20.3, 19.4, 14.6, 14.0, and 12.2; ^19^F NMR (564 MHz, CDCl_3_) *δ*: −121.1; and ESI-MS (30 eV) *m/z*: [M+H]^+^ Calcd for C_39_H_53_FN_3_O_10_^+^ 742.37, Found 742.42, [M+Na]^+^ Calcd for C_39_H_52_FN_3_NaO_10_^+^ 764.35, Found 764.56, [M+K]^+^ Calcd for C_39_H_52_FKN_3_O_10_^+^ 780.33, Found 780.36.

3-(4-(7-chloroquinolin-4-yl)piperazine-1-carbonyl)-1-cyclopropyl-6-fluoro-7-(4-(2-((3R,6R,9R,10R,12R,12aR)-3,6,9-trimethyldecahydro-12H-3,12-epoxy[1,2]dioxepino[4,3-i]isochromen-10-yl)acetyl)piperazin-1-yl)quinolin-4(1H)-one (**25**): Reaction time: 2 h; yellow solid (65 mg, 50%); mp: 160.2–161.3 °C; R_f_ (PhMe/AcOΕt 1:9): 020; ^1^H NMR (600 MHz, CDCl_3_) *δ*: 8.65 (d, *J* = 5.6 Hz, 1H), 8.25 (s, 1H), 8.15 (s, 1H), 8.01 (d, *J* = 12.8 Hz, 1H), 7.97 (d, *J* = 9.0 Hz, 1H), 7.48 (d, *J* = 8.9, 2.4 Hz, 1H), 7.31 (d, H, *J* = 7.1 Hz, 1H), 6.91 (d, *J* = 5.6 Hz, 1H), 5.34 (s, 1H), 4.91–4.86 (m, 1H), 4.10–4.04 (m, 3H), 3.86–3.80 (m, 1H), 3.73–3.65 (m, 3H), 3.56–3.48 (m, 3H), 3.45–3.37 (m, 2H), 3.32–3.27 (m, 2H), 3.2–3.15 (m, 1H), 2.8–2.71 (m, 2H), 2.5 (dd, *J* = 14.6, 4.6 Hz, 1H), 2.36–2.29 (m, 1H), 2.06–1.99 (m, 2H), 1.96–1.91 (m, 1H), 1.84–1.79 (m, 1H), 1.74–1.66 (m, 3H), 1.38 (s, 3H), 1.32 (d, *J* = 6.8 Hz, 2H), 1.30–1.24 (m, 5H), 1.17–1.14 (m, 2H), 0.97 (d, *J* = 5.9 Hz, 3H), and 0.91 (d, *J* = 7.4 Hz, 3H); ^13^C NMR (150 MHz, CDCl_3_) *δ*: 172.5, 170.1, 166.3, 153.0, 146.0, 145.6, 138.5, 126.0, 122.1, 121.4, 109.2, 107.7, 105.1, 103.0, 89.8, 81.1, 71.3, 52.1, 50.8, 49.8, 47.5, 46.1, 44.0, 42.5, 41.6, 37.7, 36.7, 35.3, 34.6, 30.4, 26.2, 25.0, 20.2, 12.7, and 8.3; ^19^F NMR (564 MHz, CDCl_3_) *δ*: −123.6; and ESI-MS (30 eV) *m/z*: [M+H]^+^ Calcd for C_47_H_55_ClFN_6_O_7_^+^ 869.38, Found 869.18, [M+Na]^+^ Calcd for C_47_H_54_ClFN_6_NaO_7_^+^ 891.36, Found 891.21.

3-(4-(7-chloroquinolin-4-yl)piperazine-1-carbonyl)-1-cyclopropyl-6-fluoro-7-(4-(3-((3R,6R,9R,10R,12R,12aR)-3,6,9-trimethyldecahydro-12H-3,12-epoxy[1,2]dioxepino[4,3-i]isochromen-10-yl)propanoyl)piperazin-1-yl)quinolin-4(1H)-one (**26**): Reaction time: 2 h; yellow solid (99 mg, 75%); mp: 115–116 °C; R_f_ (AcOΕt/MeOH 9:1): 0.21; ^1^H NMR (600 MHz, CDCl_3_) *δ*: 8.66 (d, *J* = 5.3 Hz, 1H), 8.22 (br s, 1H), 8.15 (s, 1H), 8.01 (d, *J* = 13.0 Hz, 1H), 7.97 (d, *J* = 9.0 Hz, 1H), 7.48 (dd, *J* = 9.0, 1.9 Hz, 1H), 7.30 (d, *J* = 7.0 Hz, 1H), 6.90 (d, *J* = 5.6 Hz, 1H), 5.3 (s, 1H), 4.10–4.05 (m, 2H), 3.93–3.69 (m, 6H), 3.54–3.46 (m, 3H), 3.43 (septet, *J* = 3.2 Hz, 1H), 3.32–3.22 (m, 4H), 2.80–2.70 (m, 2H), 2.45–2.39 (m, 1H), 2.33 (td, *J* = 14.3, 3.8 Hz, 1H), 2.04–1.99 (m, 2H), 1.92–1.81 (m, 3H), 1.69–1.65 (m, 1H), 1.58 (dt, *J* = 13.8, 5.0 Hz, 1H), 1.48–1.41 (m, 2H), 1.40 (s, 3H), 1.35–1.24 (m, 7H), 1.17–1.14 (m, 2H), 0.95 (d, *J* = 6.2 Hz, 3H), and 0.91 (d, *J* = 7.6 Hz, 3H); ^13^C NMR (150 MHz, CDCl_3_) *δ*: 172.5, 171.7, 166.3, 152.6, 145.6, 144.6, 138.5, 124.5, 122.2, 118.5, 113.6, 113.2, 105.0, 103.6, 88.9, 81.3, 76.4, 60.5, 52.7, 49.8, 47.5, 44.7, 42.5, 41.5, 37.6, 36.7, 34.6, 31.7, 31.1, 30.3, 26.4, 25.0, 24.8, 24.6, 22.8, 21.2, 20.4, 14.3, 13.5, and 8.3; ^19^F NMR (564 MHz, CDCl_3_) *δ*: −123.8; and ESI-MS (30 eV) *m/z*: [M+H]^+^ Calcd for C_48_H_57_ClFN_6_O_7_^+^ 883.57, Found 883.41, [M+Na]^+^ Calcd for C_48_H_56_ClFN_6_NaO_7_^+^ 905.38, Found 905.28, [M+K]^+^ Calcd for C_48_H_56_ClFKN_6_O_7_^+^ 921.35, Found 921.21.

3-(4-(7-chloroquinolin-4-yl)piperazine-1-carbonyl)-1-ethyl-6-fluoro-7-(4-(2-((3R,6R,9R,10R,12R,12aR)-3,6,9-trimethyldecahydro-12H-3,12-epoxy[1,2]dioxepino[4,3-i]isochromen-10-yl)acetyl)piperazin-1-yl)quinolin-4(1H)-one (**27**): Reaction time: 12 h; yellow solid (64 mg, 50%); mp: 173.6–174.5 °C; R_f_ (AcOΕt/MeOH 85:15): 0.32; ^1^H NMR (600 MHz, CDCl_3_) *δ*: 8.65 (d, *J* = 5.4 Hz, 1H), 8.19 (s, 1H), 8.03 (s, 1H), 8.01 (s, 1H), 7.97 (d, *J* = 9.1 Hz, 1H), 7.45 (dd, *J* = 9.0 Hz, 1H), 6.89 (d, *J* = 5.5 Hz, 1H), 6.77 (d, *J* = 6.7 Hz, 1H), 5.33 (s, 1H), 4.89–4.85 (m, 1H), 4.22 (q, *J* = 7.1 Hz, 2H), 4.05–4.03 (m, 3H), 3.84–3.79 (m, 1H), 3.74–3.70 (m, 2H), 3.69–3.64 (m, 2H), 3.53–3.47 (m, 4H), 3.38–3.34 (m, 1H), 3.31–3.25 (m, 2H), 3.17–3.12 (m, 1H), 2.79–2.70 (m, 2H), 2.49 (dd, *J* = 14.6, 4.4 Hz, 1H), 2.35–2.28 (m, 2H), 2.00 (dt, *J* = 14.3, 3.9 Hz, 1H), 1.95–1.90 (m, 1H), 1.81 (dd, *J* = 13.5, 3.5 Hz, 1H), 1.72–1.65 (m, 2H), 1.55 (t, *J* = 7.1 Hz, 3H), 1.37 (s, 3H), 1.30–1.24 (m, 4H), 0.96 (d, *J* = 5.9 Hz, 3H), and 0.90 (d, *J* = 7.5 Hz, 3H); ^13^C NMR (150 MHz, CDCl_3_) *δ*: 172.4, 170.1, 166.3, 154.1, 145.6, 144.8, 136.5, 127.1, 125.5, 122.9, 121.0, 117.1, 113.3, 108.4, 104.2, 103.1, 89.8, 81.1, 71.4, 52.8, 52.1, 50.7, 49.8, 49.0, 47.5, 46.1, 44.0, 42.6, 41.6, 37.7, 36.6, 35.2, 34.5, 30.4, 26.1, 24.9, 20.2, 14.5, and 12.7; ^19^F NMR (564 MHz, CDCl_3_) *δ*: −123.5; and ESI-MS (30 eV) *m/z*: [M+H]^+^ Calcd for C_46_H_55_ClFN_6_O_7_^+^ 857.38, Found 857.53, [M+Na]^+^ Calcd for C_46_H_54_ClFN_6_NaO_7_^+^ 879.36, Found 879.43, [M+K]^+^ Calcd for C_46_H_54_ClFKN_6_O_7_^+^ 895.34, Found 895.35.

(3R,6R,9R,10S,12R,12aR)-3,6,9-trimethyldecahydro-12H-3,12-epoxy[1,2]dioxepino[4,3-i]isochromen-10-yl 4-(4-(3-(4-(7-chloroquinolin-4-yl)piperazine-1-carbonyl)-1-cyclopropyl-6-fluoro-4-oxo-1,4-dihydroquinolin-7-yl)piperazin-1-yl)-4-oxobutanoate (**28**): Reaction time: 2 h; yellow solid (85 mg, 61%); mp: 157–158.2 °C; R_f_ (AcOΕt/MeOH 9:1): 0.21; ^1^H NMR (600 MHz, CDCl_3_) *δ*: 8.62 (d, *J* = 4.3 Hz, 1H), 8.21 (s, 1H), 8.14 (s, 1H), 7.99–7.94 (m, 2H), 7.46 (dd, *J* = 8.9, 1.4 Hz, 1H), 7.30 (d, *J* = 6.9 Hz, 1H), 6.89 (d, *J* = 5.4 Hz, 1H), 5.79 (d, *J* = 9.8 Hz, 1H), 5.42 (s, 1H), 4.09–4.02 (m, 2H), 3.87–3.82 (m, 2H), 3.75–3.70 (m, 4H), 3.57–3.52 (m, 3H), 3.47–3.44 (m, 1H), 3.32–3.29 (m, 2H), 3.25–3.21 (m, 2H), 2.82 (t, *J* = 6.6 Hz, 1H), 2.79–2.73 (m, 2H), 2.68–2.62 (m, 1H), 2.59–2.53 (m, 1H), 2.38–2.34 (m, 1H), 2.02 (dt, *J* = 14.6, 2.9 Hz, 1H), 1.91–1.86 (m, 1H), 1.80–1.76 (m, 1H), 1.72–1.68 (m, 1H), 1.61 (dt, *J* = 13.7, 4.4 Hz, 1H), 1.41 (s, 3H), 1.34–1.30 (m, 3H), 1.28–1.25 (m, 2H), 1.20–1.13 (m, 4H), 1.02–0.99 (m, 1H), 0.95 (d, *J* = 6.1 Hz, 3H), and 0.87 (d, *J* = 7.1 Hz, 3H); ^13^C NMR (150 MHz, CDCl_3_) *δ*: 172.4, 171.9, 169.8, 166.3, 158.5, 154.2, 152.6, 145.6, 144.5, 138.5, 137.1, 129.1, 128.3, 127.2, 125.7, 122.1, 120.6, 116.9, 112.9, 108.2, 105.2, 104.6, 92.3, 91.6, 80.2, 52.7, 52.0, 51.7, 50.4, 49.8, 47.4, 45.3, 42.5, 41.6, 37.4, 36.3, 34.6, 34.2, 31.9, 29.8, 29.5, 27.7, 26.1, 24.7, 22.1, 20.3, 12.2, and 8.3; ^19^F NMR (564 MHz, CDCl_3_) *δ*: −123.9; and ESI-MS (30 eV) *m/z*: [M+H]^+^ Calcd for C_49_H_57_ClFN_6_O_9_^+^ 927.39, Found 927.22, [M+Na]^+^ Calcd for C_49_H_56_ClFN_6_O_9_^+^ 949.37, Found 949.26.

(3R,6R,9R,10S,12R,12aR)-3,6,9-trimethyldecahydro-12H-3,12-epoxy[1,2]dioxepino[4,3-i]isochromen-10-yl 4-(4-(3-(4-(7-chloroquinolin-4-yl)piperazine-1-carbonyl)-1-ethyl-6-fluoro-4-oxo-1,4-dihydroquinolin-7-yl)piperazin-1-yl)-4-oxobutanoate (**29**): Reaction time: 12 h; yellow solid (88 mg, 64%); mp: 168.7–169.6 °C; R_f_ (AcOΕt/MeOH 87:13): 0.26; ^1^H NMR (600 MHz, CDCl_3_) *δ*: 8.71 (d, *J* = 4.9 Hz, 1H), 8.06–8.02 (m, 3H), 7.96 (d, *J* = 9.0 Hz, 1H), 7.45 (dd, *J* = 9.0, 1.9 Hz, 1H), 6.87 (d, *J* = 5.0 Hz, 1H), 6.75 (d, *J* = 6.6 Hz, 1H), 5.77 (d, *J* = 9.8 Hz, 1H), 5.41 (s, 1H), 4.19 (q, *J* = 7.1 Hz, 2H), 4.08–4.00 (m, 2H), 3.85–3.82 (m, 2H), 3.73–3.69 (m, 4H), 3.32–3.25 (m, 6H), 3.22–3.18 (m, 2H), 2.86–2.80 (m, 1H), 2.78–2.71 (m, 2H), 2.67–2.61 (m, 1H), 2.58–2.53 (m, 1H), 2.34 (td, *J* = 14.1, 3.7 Hz, 2H), 2.03–1.98 (m, 1H), 1.90–1.85 (m, 1H), 1.78–1.73 (m, 1H), 1.71–1.67 (m, 1H), 1.60 (dt, *J* = 13.7, 4.4 Hz, 1H), 1.54 (t, *J* = 7.2 Hz, 3H), 1.40 (s, 3H), 1.35 (dd, *J* = 13.7, 3.2 Hz, 1H), 1.27–1.22 (m, 2H), 1.03–0.97 (m, 1H), 0.94 (d, *J* = 6.1 Hz, 3H), and 0.86 (d, *J* = 7.1 Hz, 3H); ^13^C NMR (150 MHz, CDCl_3_) *δ*: 172.3, 171.9, 169.8, 166.2, 156.9, 154.0, 152.4, 151.9, 150.1, 145.4, 136.5, 135.2, 128.9, 126.5, 125.1, 123.0, 122.0, 117.4, 113.5, 109.4, 104.6, 104.1, 92.2, 91.6, 80.2, 60.5, 52.8, 52.2, 51.7, 50.5, 48.9, 45.3, 42.6, 41.6, 37.4, 36.3, 34.2, 31.9, 29.5, 27.7, 26.0, 24.7, 22.1, 20.3, 14.5, 14.3, and 12.2; ^19^F NMR (564 MHz, CDCl_3_) *δ*: −123.7; and ESI-MS (30 eV) *m/z*: [M+H]^+^ Calcd for C_48_H_57_ClFN_6_O_9_^+^ 915.39, Found 915.43, [M+Na]^+^ Calcd for C_49_H_56_ClFN_6_NaO_9_^+^ 937.37, Found 937.39, [M+K]^+^ Calcd for C_49_H_56_ClFKN_6_O_9_^+^ 953.34, Found 953.79.

### 3.3. Biological Evaluation

#### 3.3.1. Cytototoxicity upon Human *Primary fibroblast* Cell Line AB934

A cellular cytotoxicity evaluation was performed upon human primary fibroblasts AB943, as previously described [36]. The assays were realized in 96-well plates in DMEM + Glutamax without phenol red medium containing 25 mM HEPES, a pH of 7.3, and 10% of fetal calf serum under a 5% CO_2_ atmosphere, at 37 °C. After trypsin treatment, the AB943 cells were seeded at 2000 cells per well in 100 μL. After 24 h of incubation, drugs diluted in the culture medium were added (100 μL per well). The drug stock solutions were prepared in dimethyl sulfoxide (DMSO). The final DMSO concentration in the cultures remained below 1%. Control cultures were constituted of cultures treated with DMSO instead of the drug. The cytotoxicity assay was based on the conversion of a redo-sensitive dye (resazurin) to a fluorescent product via viable cells. After 72 h of incubation, the resazurin solution was added in each well at a final concentration of 45 μM. The fluorescence was measured at 530 nm excitation and 590 nm emission wavelengths after 4 h of incubation. The percentage of the inhibition of cell growth was calculated by comparing the fluorescence of cells maintained in the presence of the drug to that in the absence of the drug. The concentration causing 50% growth inhibition (IC_50_) was obtained from the drug concentration–response curve, and the results were expressed as the mean values ± standard deviations determined from several experiments.

#### 3.3.2. In Vitro Growth Inhibition of *P. falciparum*

The chloroquine-resistant FcB1/Colombia and Dd2/Indochina strains of *Plasmodium falciparum* were obtained from the Protist Collection of the National Museum of Natural History, Paris, France (n° MNHN-CEU-2016-224-PfFCB1 and MNHN-CEU-2016-222-PfDD2). The parasites were maintained in vitro on human erythrocytes in RPMI 1640 medium supplemented by 8% (*v*/*v*) heat-inactivated human serum, at 37 °C, under an atmosphere of 5% CO_2_. The human red blood cells and serum were provided by the Établissement Français du Sang under the C-CPSL-UNT approval no. 13/EFS/126. The in vitro drug susceptibility assays were measured by [^3^H]-hypoxanthine incorporation, as previously described [37]. The drug stock solutions were prepared in DMSO. Briefly, the assays were performed in 96-well plates. The compounds were diluted in the culture medium (100 μL per well). The parasite cultures (100 μL, 2% parasitaemia, and 1% final hematocrit) were then added to each well and incubated for 24 h at 37 °C prior to the addition of 0.5 μCi of [^3^H]- hypoxanthine (GE Healthcare, Paris, France, 1 to 5 Ci·mmol/mL) per well. After a further incubation of 24 h, the plates were frozen and thawed. The cell lysates were then collected onto fiberglass filters and counted in a liquid scintillation spectrometer. The growth inhibition for each drug concentration was determined through a comparison of the radioactivity incorporated in the treated culture with that in the control culture maintained on the same plate. The drugs were first evaluated for a range of fixed concentrations of 50, 5, 0.5, 0.05, and 0.005 μg/mL. The most active molecules were then tested with a twofold dilution to cover a concentration range from 500 to 0.2 ng/mL. The IC_50_ value was obtained from the drug concentration–response curve, and the results were expressed in μM or nM as the mean values ± standard deviations determined from several independent experiments. Artesunate (**46**) was used as an antimalarial drug control.

## 4. Conclusions

In this paper, we conducted the synthesis and antiplasmodial evaluation of novel hybrid molecules that combine the pharmacophore structures of ART, CPX or NRX, and 7-chloroquinoline. The key piperazine esters intermediates bearing the drugs CPX and NRX constitute the scaffolds where the ART derivatives and 7-chloroquinoline core were sequentially introduced in two different positions. Once the multistep synthesis was engaged, we synthesized the 18 final compounds with overall yields ranging between 8 and 20%, starting from commercially available CPX and NRX. All compounds were biologically evaluated for their antiplasmodial activity against the CQ-resistant *P. falciparum* FcB1 strain. The most potent compounds, **20**, **21**, **22**, and **28**, which combine an artesunate fragment with CPX, exhibited IC_50_ values in a range of 3.5–5.4 nM and excellent SIs against human primary fibroblasts AB943 (SIs between 4000 and 11,000). For compounds combining the artesunate moiety with the NRX skeleton, two of them, namely compounds **23** and **24**, showed IC_50_ values of 1.5 nM and 1.9 nM, along with excellent SIs of 28,000 and 16,000, respectively. Furthermore, the same compounds, when evaluated against the CQ-resistant Dd2 strain of *P. falciparum*, exhibited activities varying between 3.5 and 17.7 nM. Apparently, the combination of artesunate with CPX or NRX in a single molecular entity proved to substantially enhance activity and selectivity, when compared to the combinations of unconjugated counterparts artesunate/CPX and artesunate/NRX.

Considering that one powerful tool to study the mechanism of action of the most potent compounds **22** and **23** could be fluorescence-based techniques, we envisage the implementation of suitable coumarin fragments as imaging probes in the future. This kind of approach has been used successfully by others for studying inhibitors against HAD proteins in *Mycobacteriem tuberculosis* [38] and most recently against CQ-resistant and multidrug-resistant *P. falciparum* strains [39].

## Figures and Tables

**Table 5:** Comparison of the antiplasmodial activities of the hybrid compounds (**22**, **23**, and **14**) and their CPX or NRX fragments (**39**, **40**, and **41**) alone or in an equimolar combination with artesunate (AS) against the CQ-resistant *P. falciparum* FcB1 strain.

Compound	IC_50_ FcB1
**39**	7.0 +/− 0.4 μM
**40**	45.7 +/− 7.6 μM
**41**	8.6 +/− 1.0 μM
AS	4.5 +/− 0.4 nM
**39** + AS	4.4 +/− 0.4 nM ^a^
**22**	3.5 +/− 0.4 nM
**40** + AS	5.1 +/− 0.6 nM ^a^
**23**	1.5 +/− 0.1 nM
**41** + AS	5.1 +/−0.6 nM ^a^
**24**	1.9 +/− 0.3 nM

^a^: The IC_50_ values of the drug combinations are expressed as equivalent to the AS present in the mixture.

## Data Availability

Data are contained within the article and Supplementary Materials.

## Supplementary Materials

The following supporting information can be downloaded at https://www.mdpi.com/article/10.3390/antibiotics13020142/s1: Copies of NMR spectra (Figures S1–S36).
