# Supplementary material for: Synthesis of Novel Artemisinin, Ciprofloxacin, and Norfloxacin Hybrids with Potent Antiplasmodial Activity
Source: Antibiotics (Basel). 2024 Feb 1;13(2):142. doi: 10.3390/antibiotics13020142 (PMC10886162; doi:10.3390/antibiotics13020142)
Supplement: Supplementary file 1 [file antibiotics-13-00142-s001.zip › antibiotics-2791113-supplementary.pdf]

SUPPORTING INFORMATION

Synthesis of Novel Artemisinin- Ciprofloxacin and Norfloxacin Hybrids with Potent Antiplasmodial Activity

Georgia Vamvoukaki[1], Antonia I. Antoniou[1], Michel Baltas[2]*, Elisabeth Mouray[3], Sebastien Charneau[3, 4], Plilippe Grellier[3] and Constantinos M. Athanassopoulos[1]*

[1] Synthetic Organic Chemistry Laboratory, Department of Chemistry,University of Patras, GR-26504 Patras, Greece; georgiavamv.gv@gmail.com (G.V.); tonadoniou@upatras.gr (A.I.A.); kath@chemistry.upatras.gr (C.M.A.)

[2] CNRS, LCC (Laboratoire de Chimie, de Coordination), Université de Tolouse, UPS, INPT, 205 Route de Narbonne, BP 44099, CEDEX 4 F-31077 Toulouse, France; michel.baltas@lcc-toulouse.fr (M.B.)

[3] MCAM, UMR 7245, Muséum National d'Histoire Naturelle, CNRS, CP52, 63 rue Buffon, 75005 Paris, France; mouray@mnhn.fr (E.M); grellier@mnhn.fr (P.G)

[4] Laboratory of Biochemistry and Protein Chemistry, Department of Cell Biology, Institute of Biology, University of Brasilia, DF, Brasil; charneau@unb.br (S.C.)

* Correspondence: kath@chemistry.upatras.gr; Tel.: +30-2610-997909 (C.M.A) and michel.baltas@lcc-toulouse.fr (M.B.)

Table of Contents

***Figure S1. ^{1}H-NMR spectrum of compound 12.* 4**

***Figure S2. ^{13}C-NMR spectrum of compound 12.* 5**

***Figure S3. ^{1}H-NMR spectrum of compound 13.* 6**

***Figure S4. ^{13}C-NMR spectrum of compound 13.* 7**

***Figure S5. ^{1}H-NMR spectrum of compound 14.* 8**

***Figure S6. ^{13}C-NMR spectrum of compound 14.* 9**

***Figure S7. ^{1}H-NMR spectrum of compound 15.* 10**

***Figure S8. ^{13}C-NMR spectrum of compound 15.* 11**

***Figure S9. ^{1}H-NMR spectrum of compound 16.* 12**

***Figure S10. ^{13}C -NMR spectrum of compound 16.* 13**

***Figure S11. ^{1}H-NMR spectrum of compound 17.* 14**

***Figure S12. ^{13}C-NMR spectrum of compound 17.* 15**

***Figure S13. ^{1}H-NMR spectrum of compound 18.* 16**

***Figure S14. ^{13}C-NMR spectrum of compound 18.* 17**

***Figure S15. ^{1}H-NMR spectrum of compound 19.* 18**

***Figure S16. ^{13}C-NMR spectrum of compound 19.* 19**

***Figure S17. ^{1}H-NMR spectrum of compound 20.* 20**

***Figure S18. ^{13}C-NMR spectrum of compound 20.* 21**

***Figure S19. ^{1}H-NMR spectrum of compound 21.* 22**

***Figure S20. ^{13}C-NMR spectrum of compound 21.* 23**

***Figure S21. ^{1}H-NMR spectrum of compound 22.* 24**

***Figure S22. ^{13}C-NMR spectrum of compound 22.* 25**

***Figure S23. ^{1}H-NMR spectrum of compound 23.* 26**

***Figure S24. ^{13}C-NMR spectrum of compound 23.* 27**

***Figure S25. ^{1}H-NMR spectrum of compound 24.* 28**

***Figure S26. ^{13}C-NMR spectrum of compound 24.* 29**

***Figure S27. ^{1}H-NMR spectrum of compound 25.* 30**

***Figure S28. ^{13}C-NMR spectrum of compound 25.* 31**

***Figure S29. ^{1}H-NMR spectrum of compound 26.* 32**

***Figure S30. ^{13}C-NMR spectrum of compound 26.* 33**

***Figure S31. ^{1}H-NMR spectrum of compound 27.* 34**

***Figure S32. ^{13}C-NMR spectrum of compound 27.* 35**

Figure S33. ^{1}***H-NMR spectrum of compound 28.*** **...36**
Figure S34. ^{13}***C-NMR spectrum of compound 28.*** **...37**
Figure S35. ^{1}***H-NMR spectrum of compound 29.*** **...38**
Figure S36. ^{13}***C-NMR spectrum of compound 29.*** **...39**

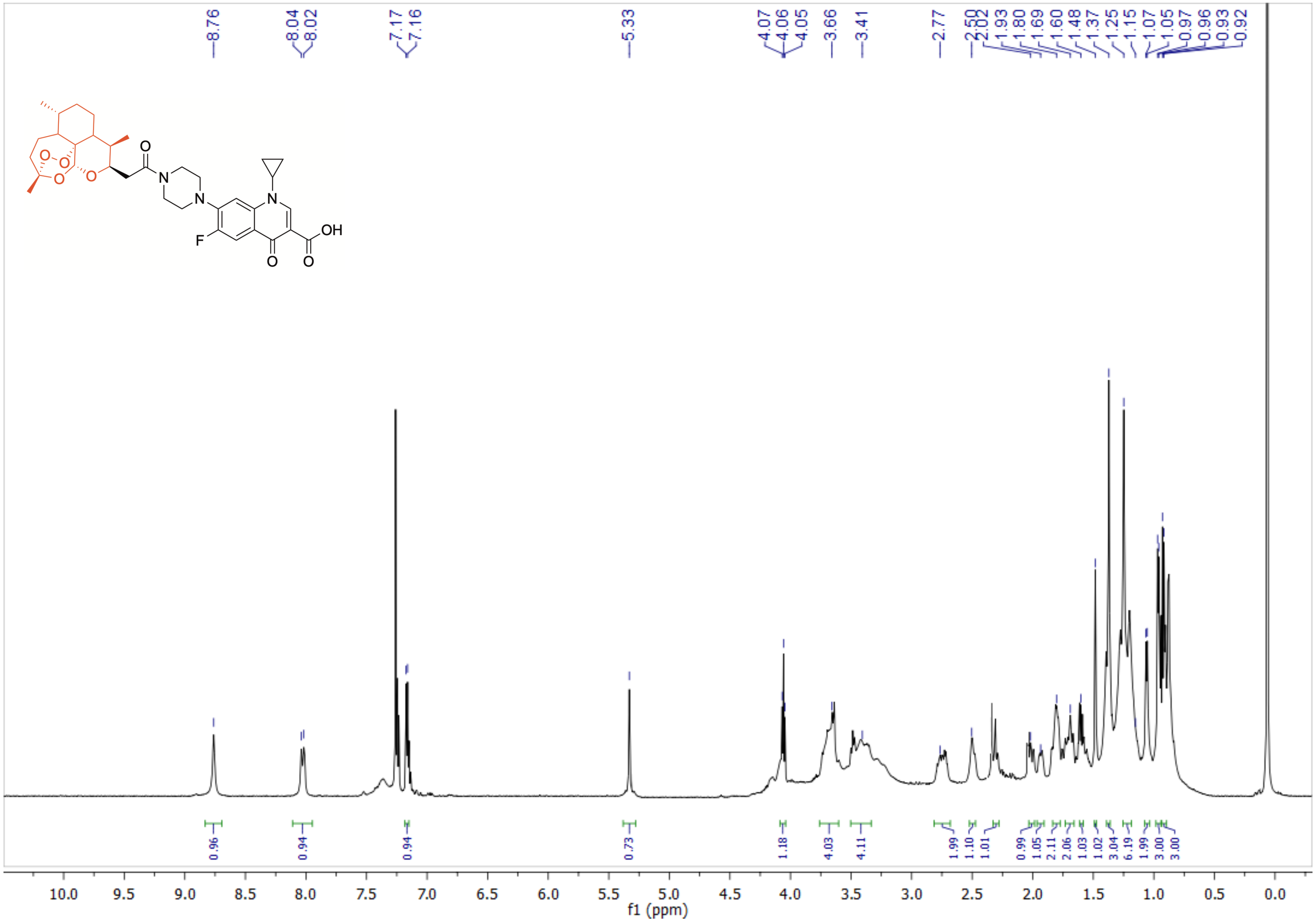

Figure S1. ^{1}H-NMR spectrum of compound 12.

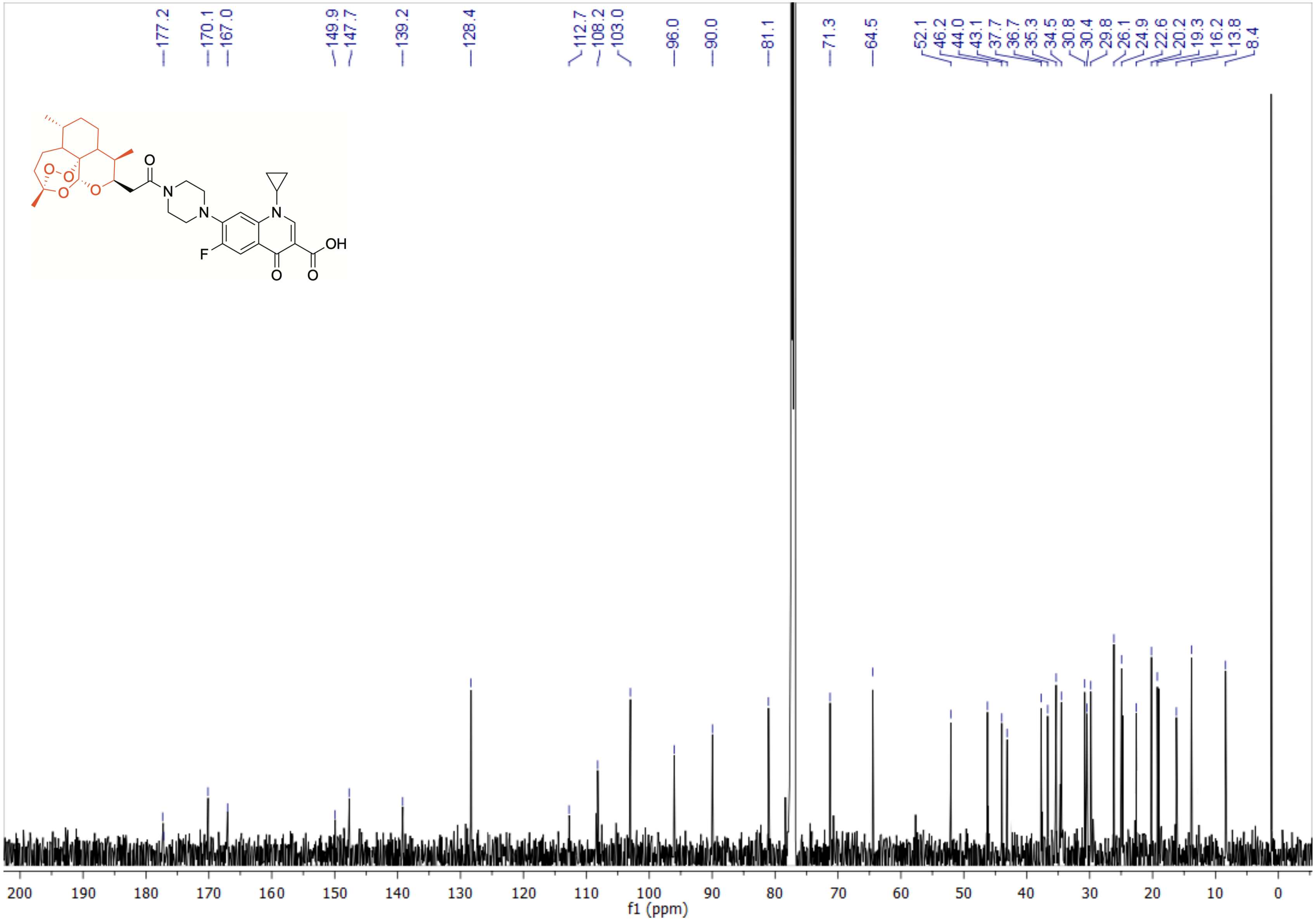

Figure S2. [13]**C-NMR spectrum of compound 12.**

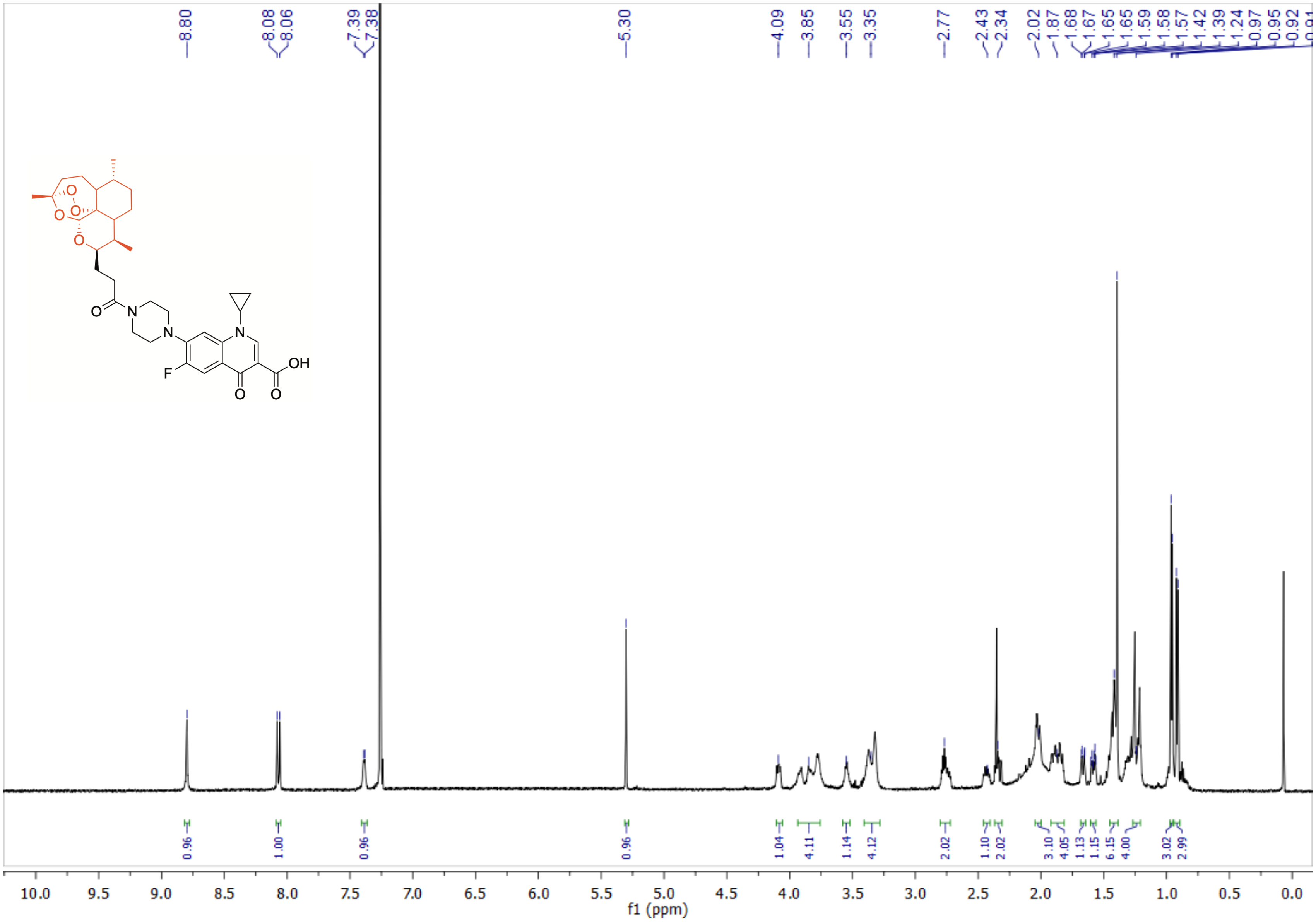

Figure S3. [1]**H-NMR spectrum of compound 13.**

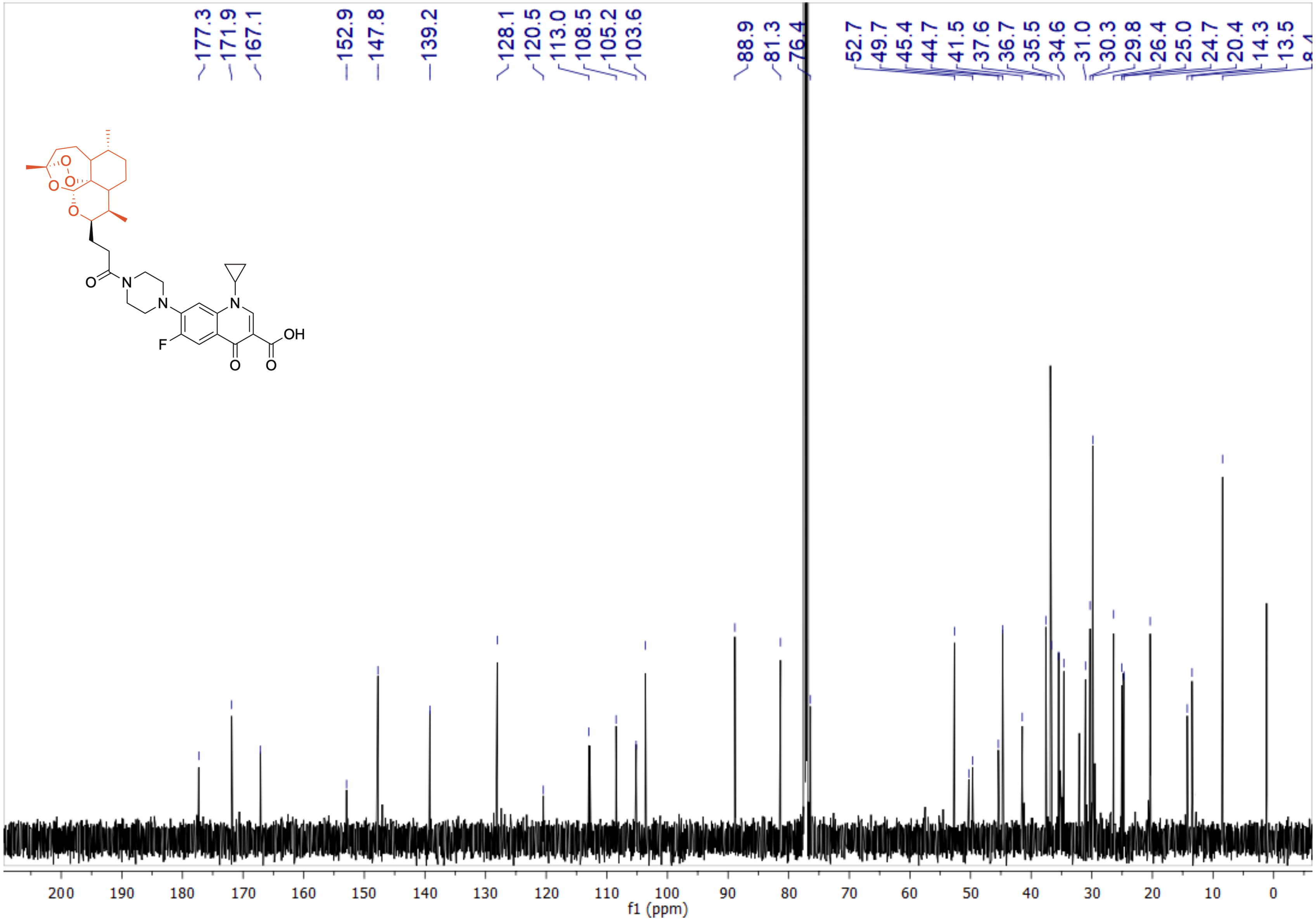

Figure S4. [13]**C-NMR spectrum of compound 13.**

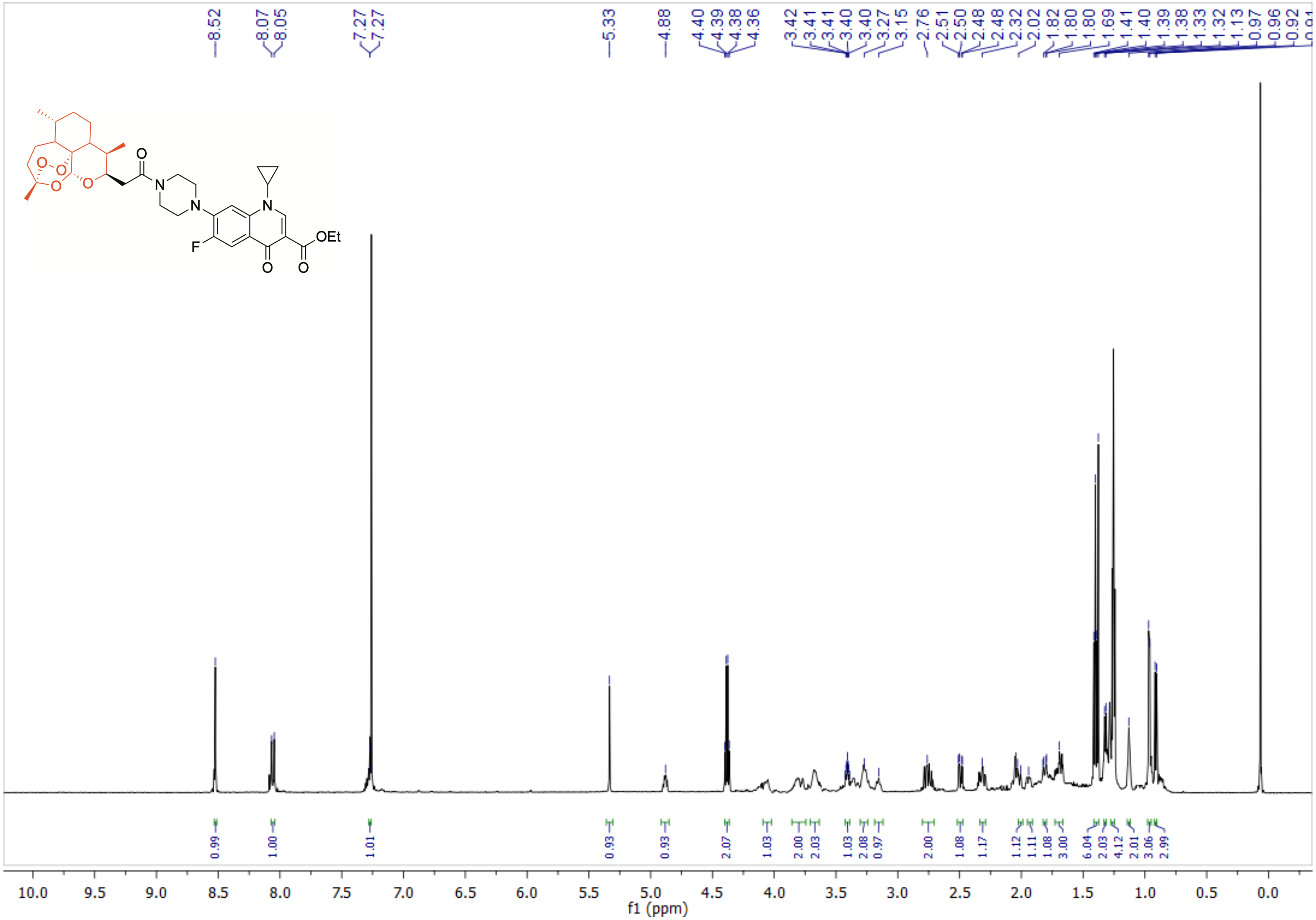

Figure S5. [1]**H-NMR spectrum of compound 14.**

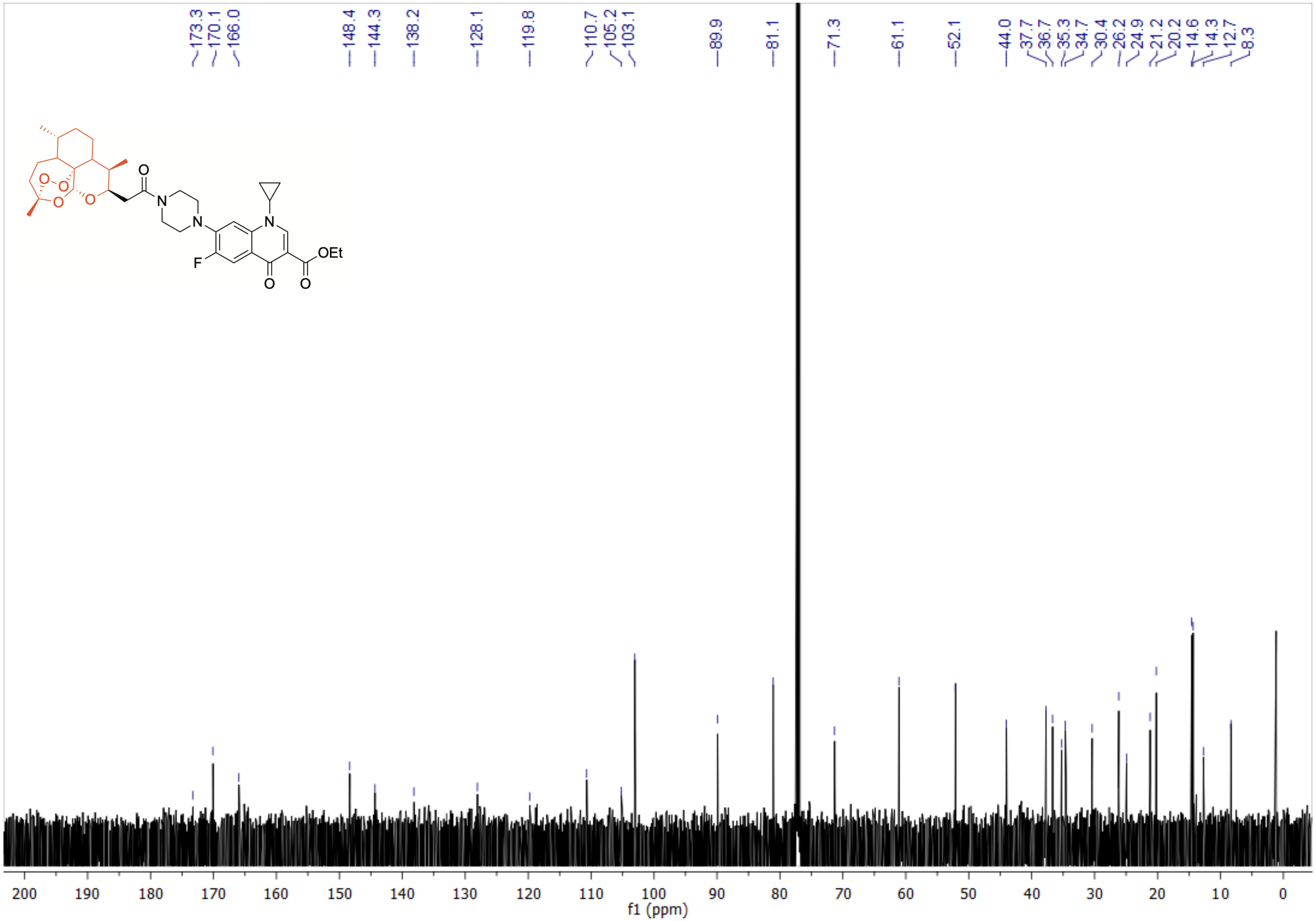

Figure S6. ^{13}C-NMR spectrum of compound 14.

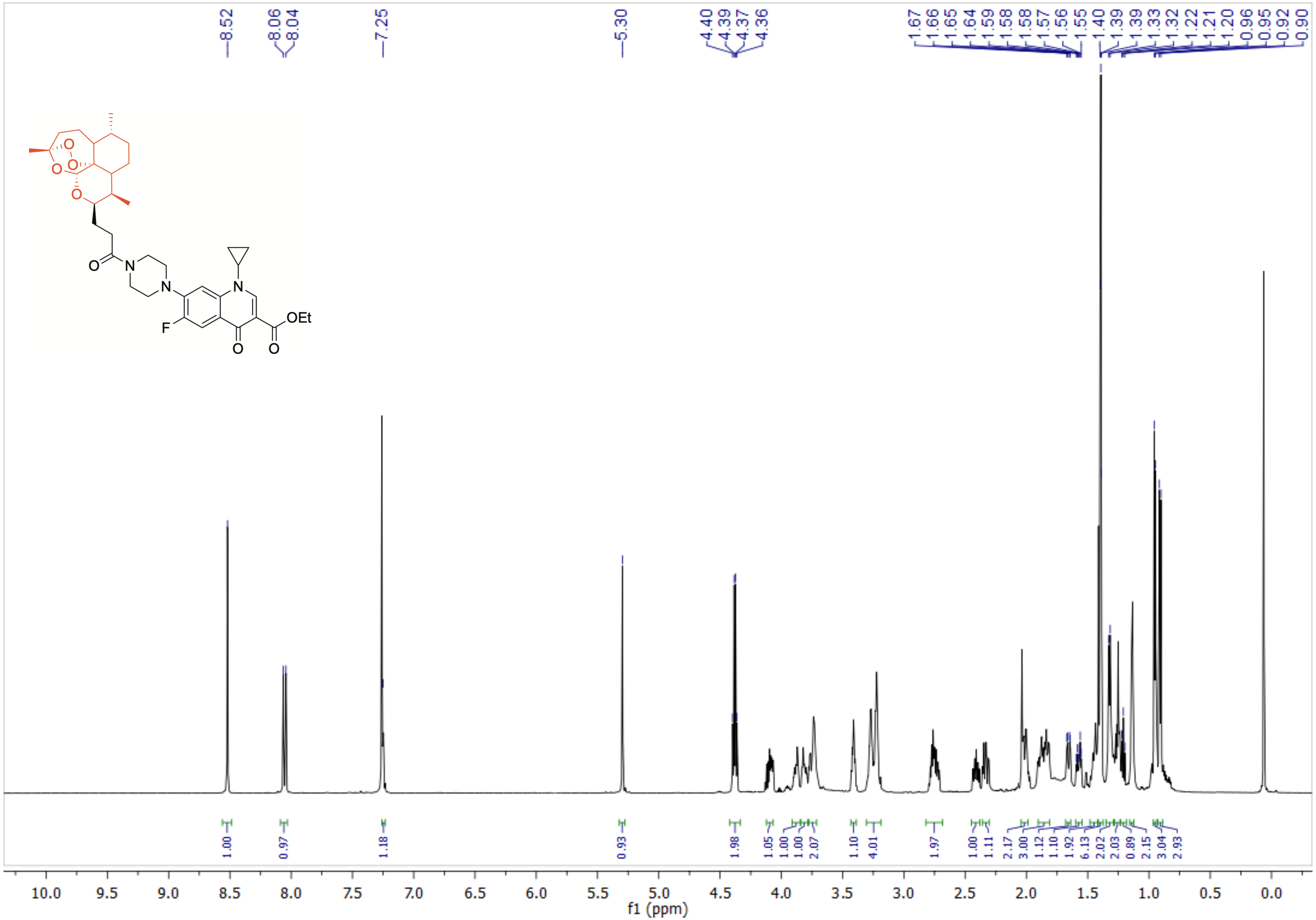

Figure S7. ^{1}H-NMR spectrum of compound 15.

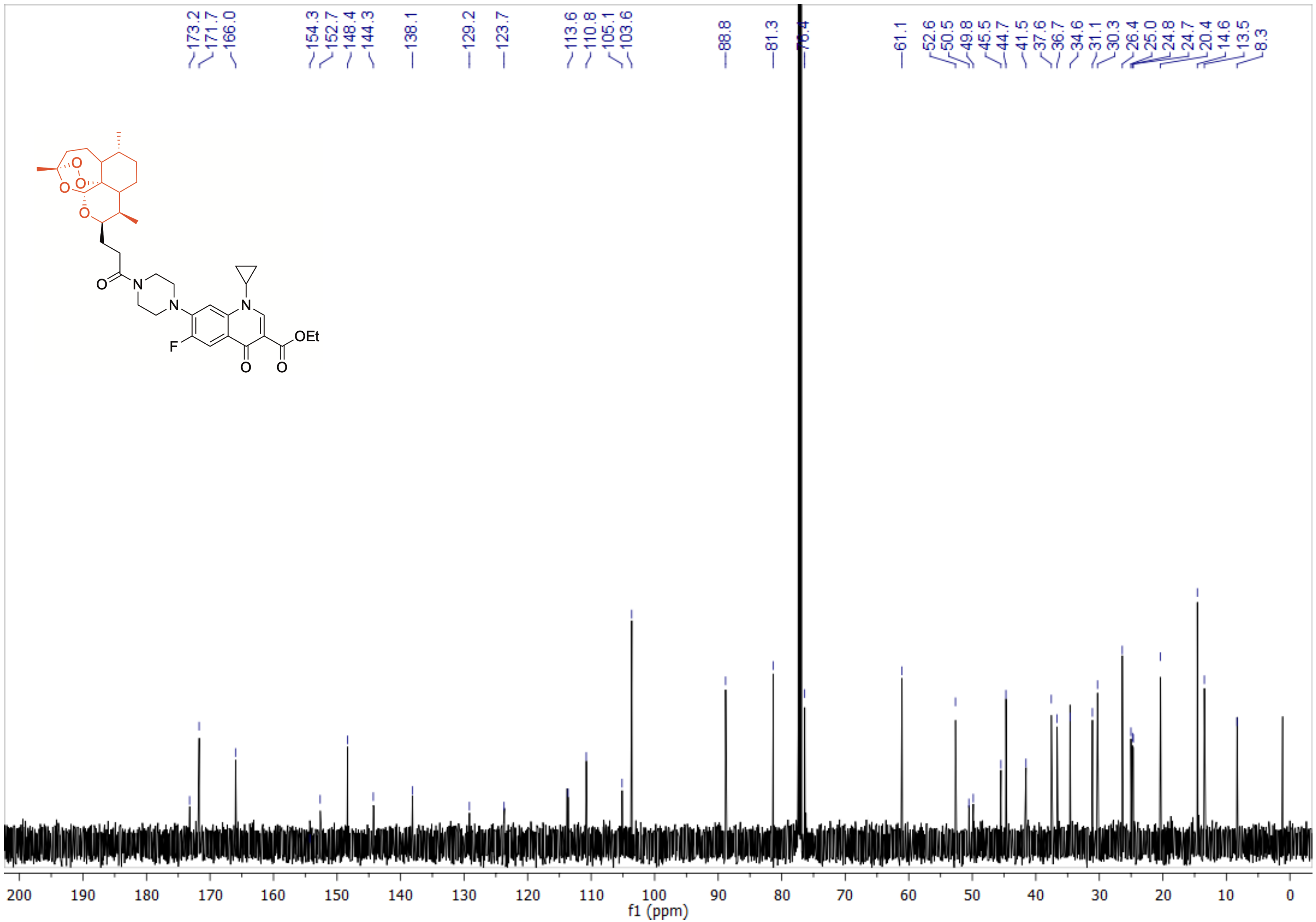

Figure S8. ^{13}C-NMR spectrum of compound 15.

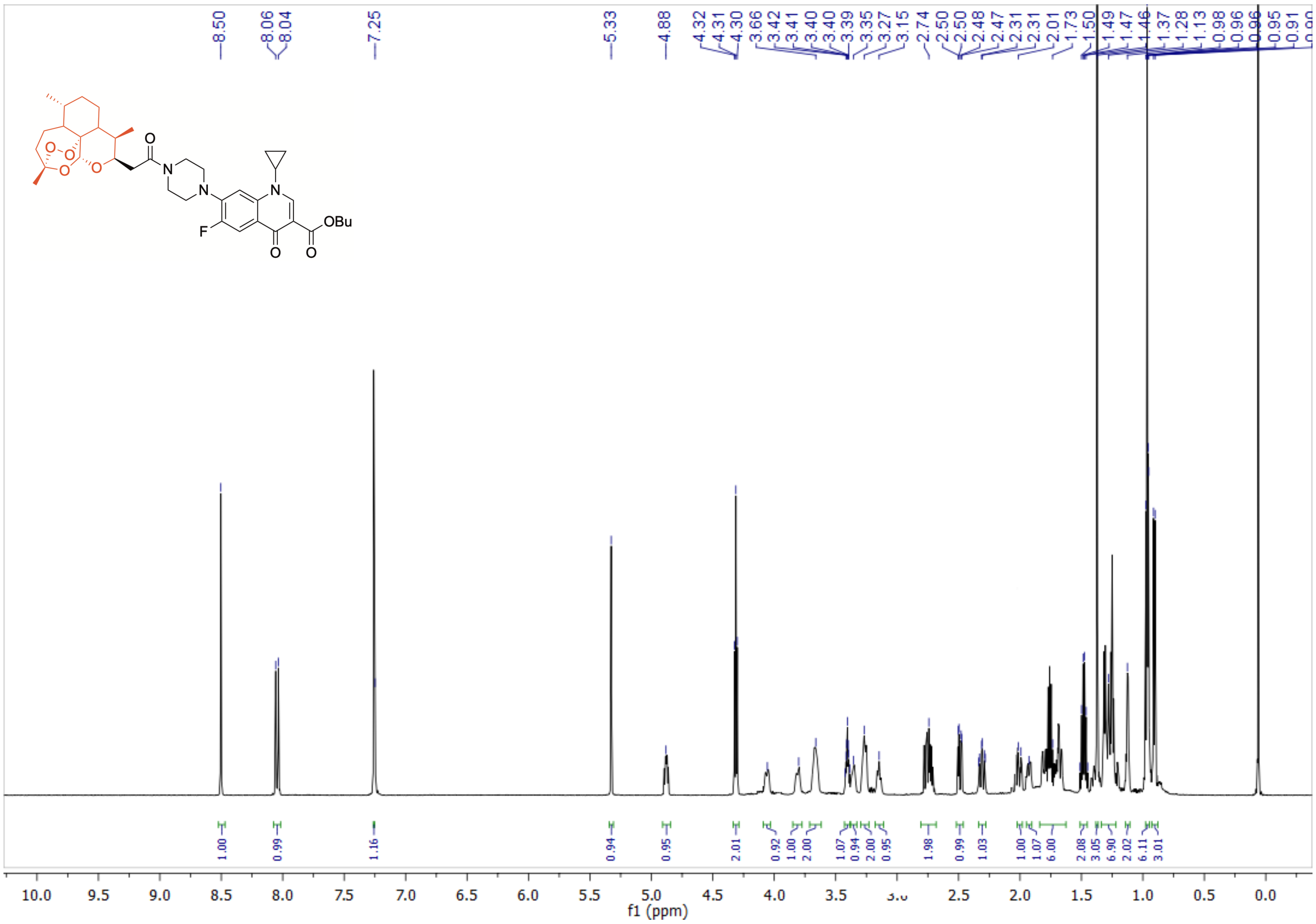

Figure S9. ¹H-NMR spectrum of compound 16.

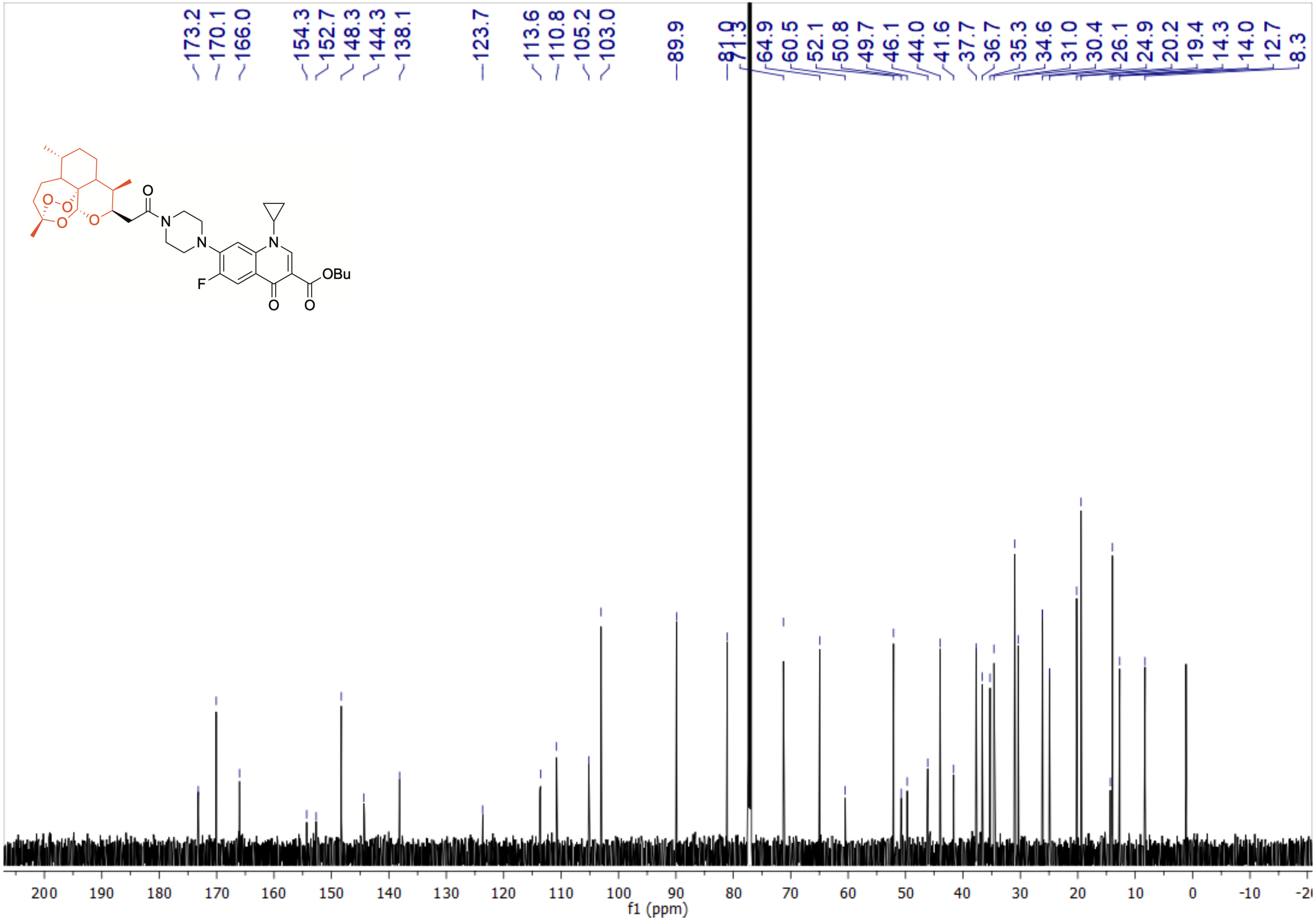

Figure S10. ^{13}**C -NMR spectrum of compound 16.**

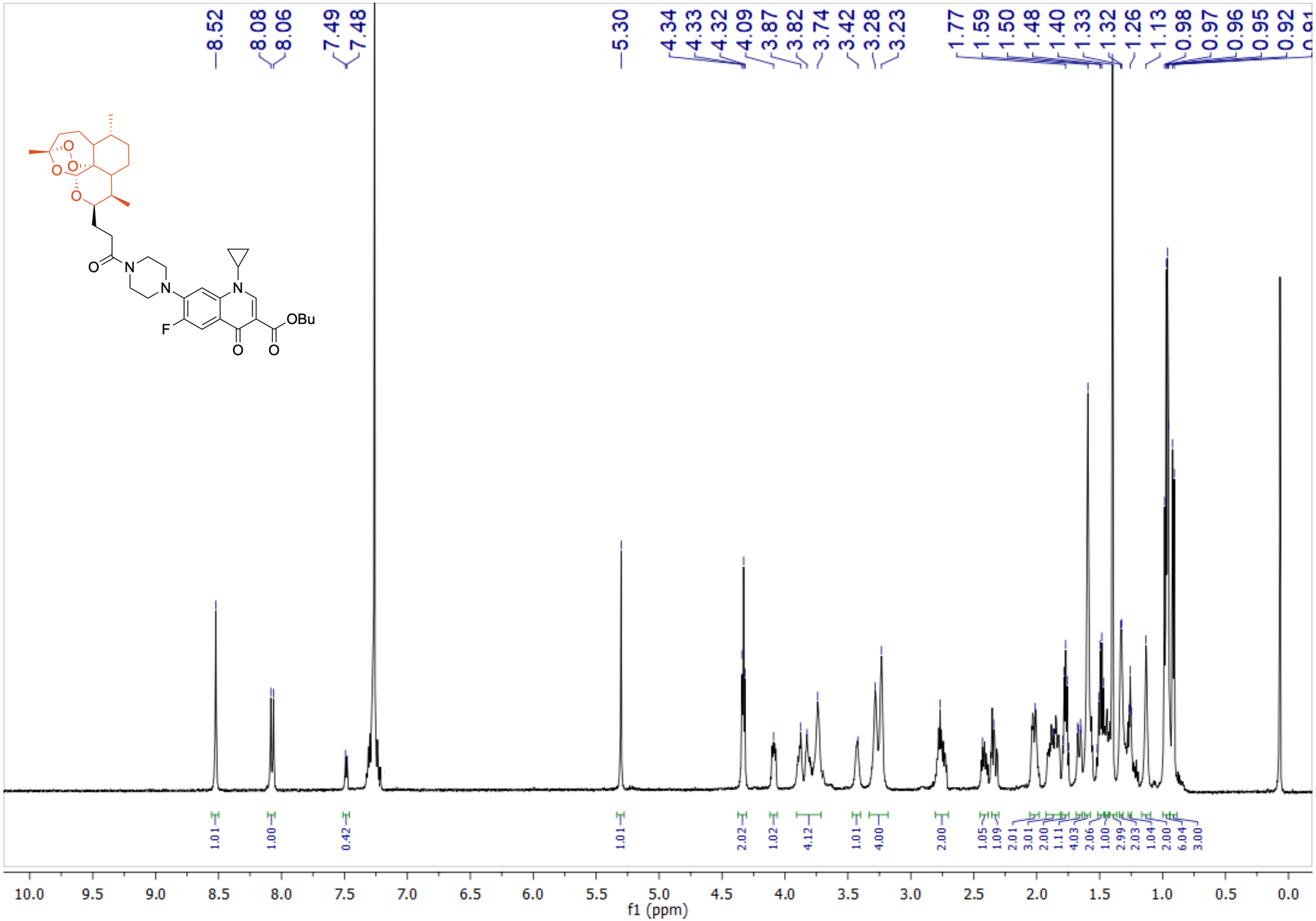

Figure S11. ^{1}H-NMR spectrum of compound 17.

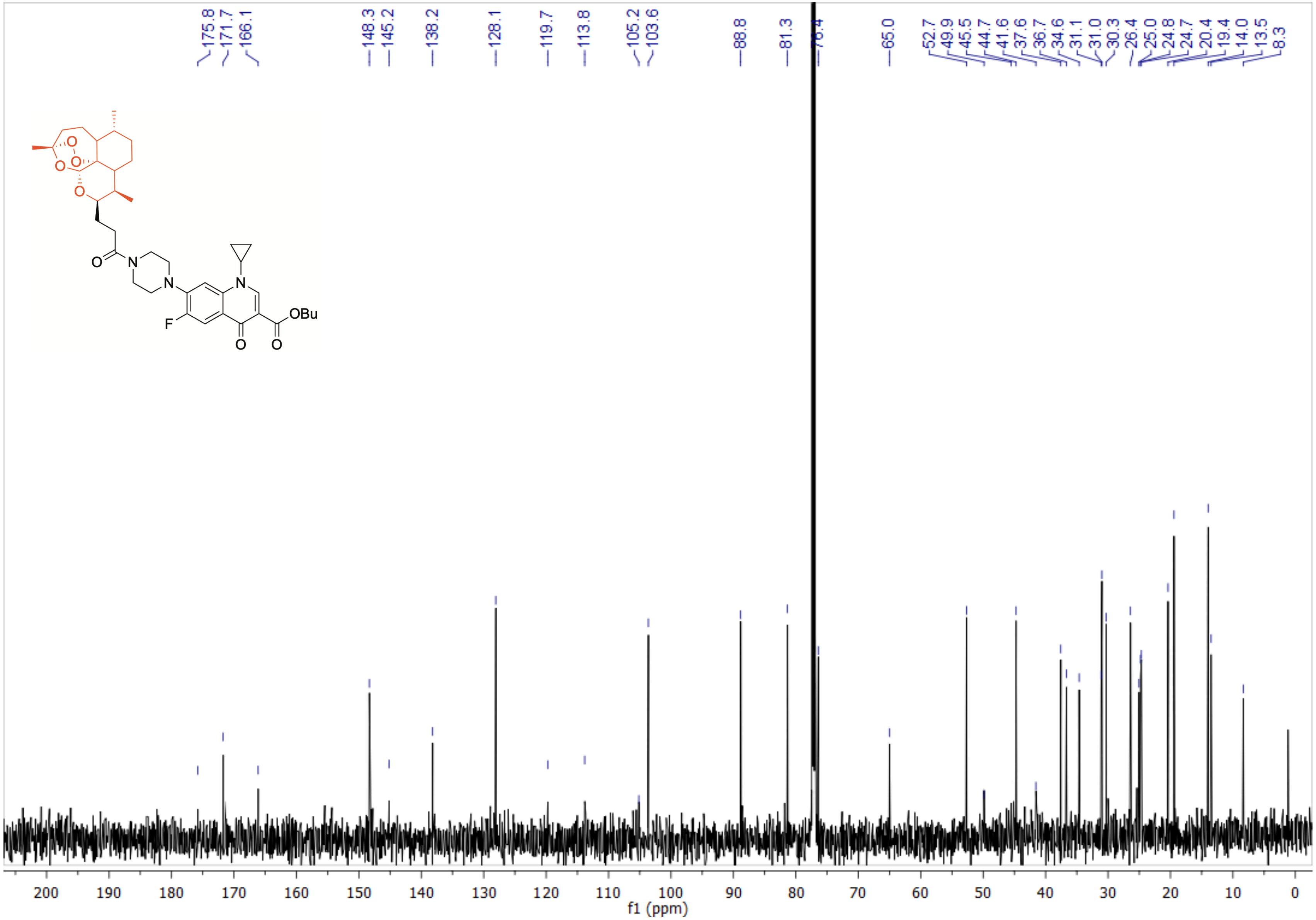

Figure S12. ^{13}**C-NMR spectrum of compound 17.**

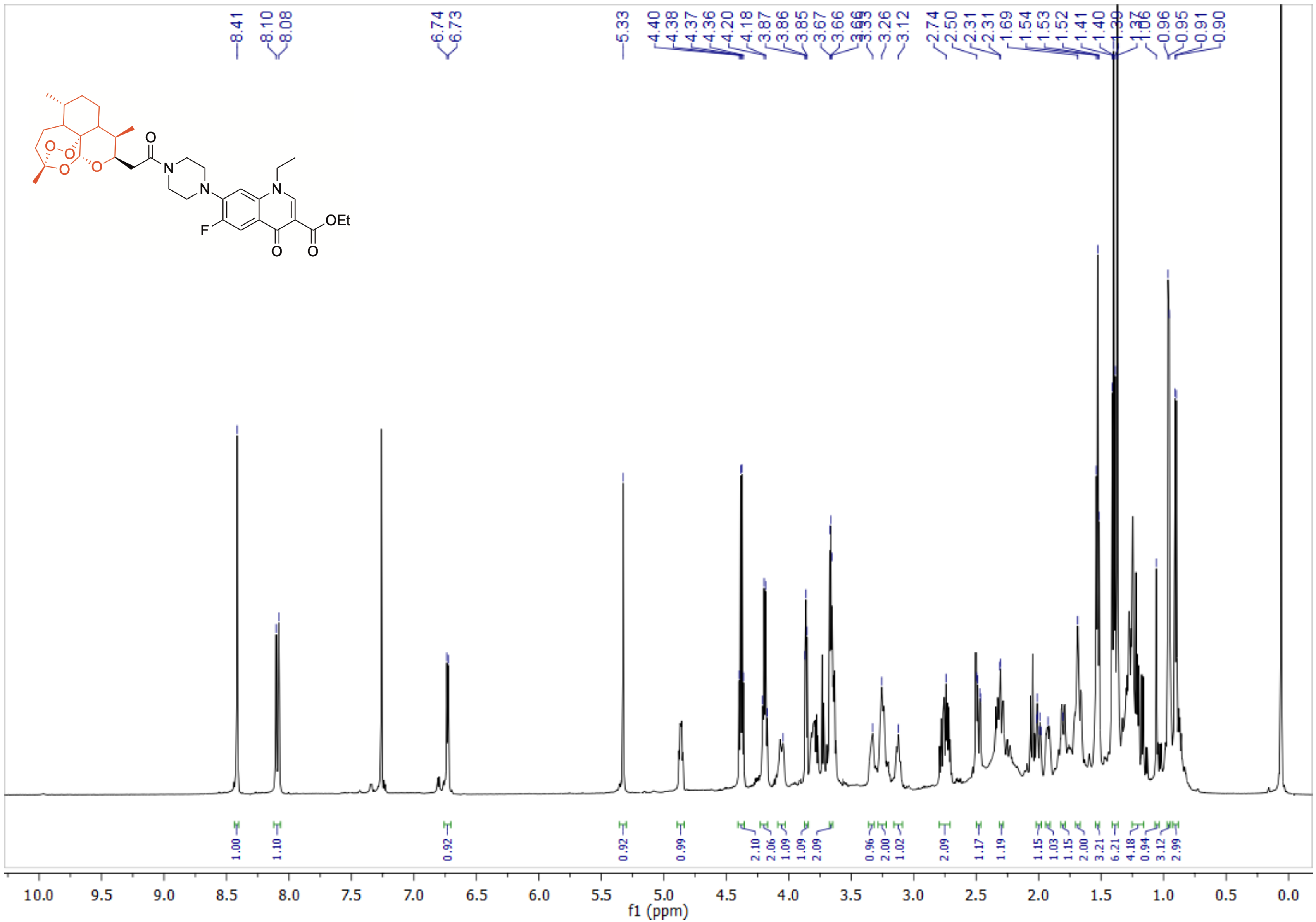

Figure S13. ^{1}H-NMR spectrum of compound 18.

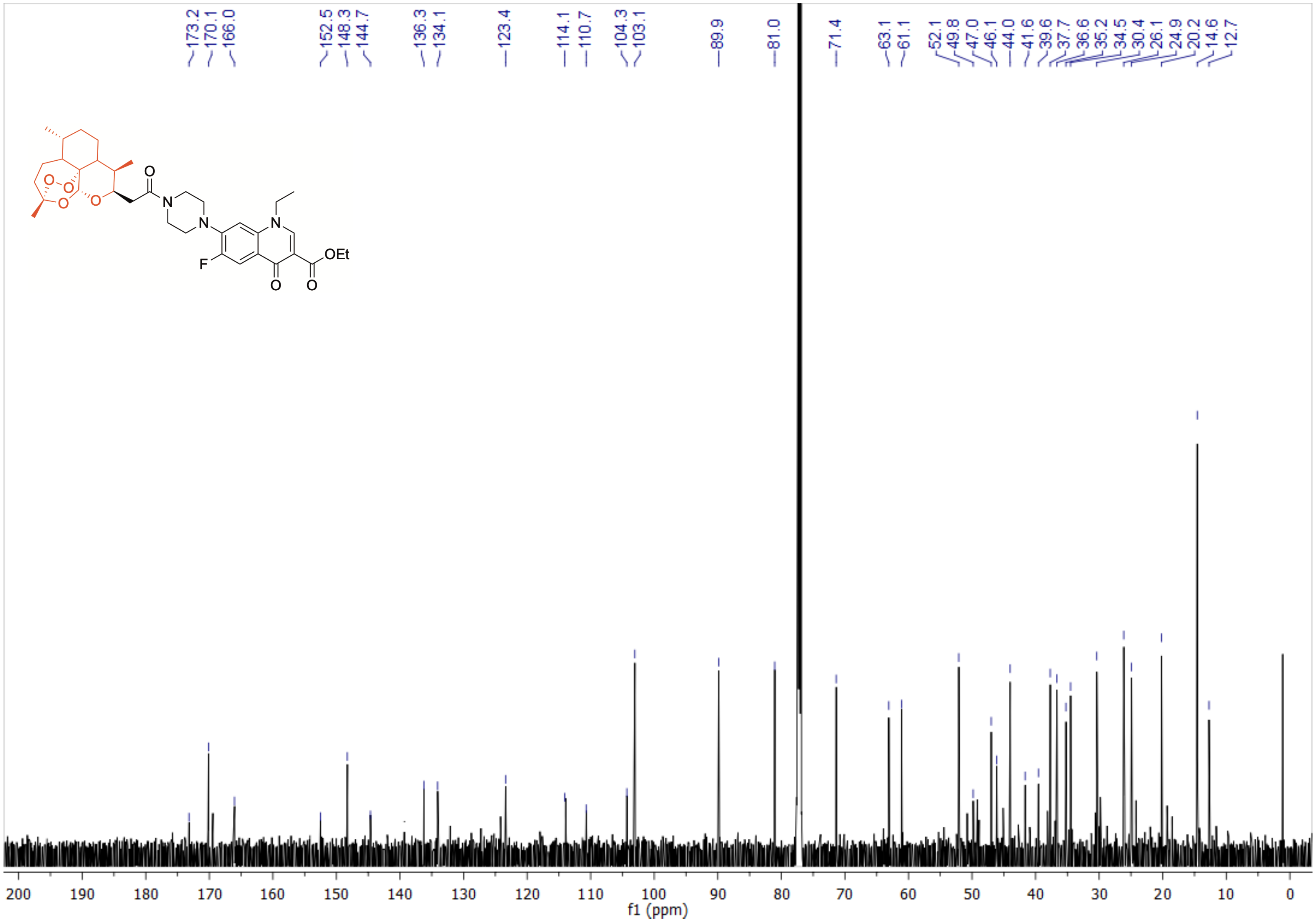

Figure S14. **^{13}C-NMR spectrum of compound 18.**

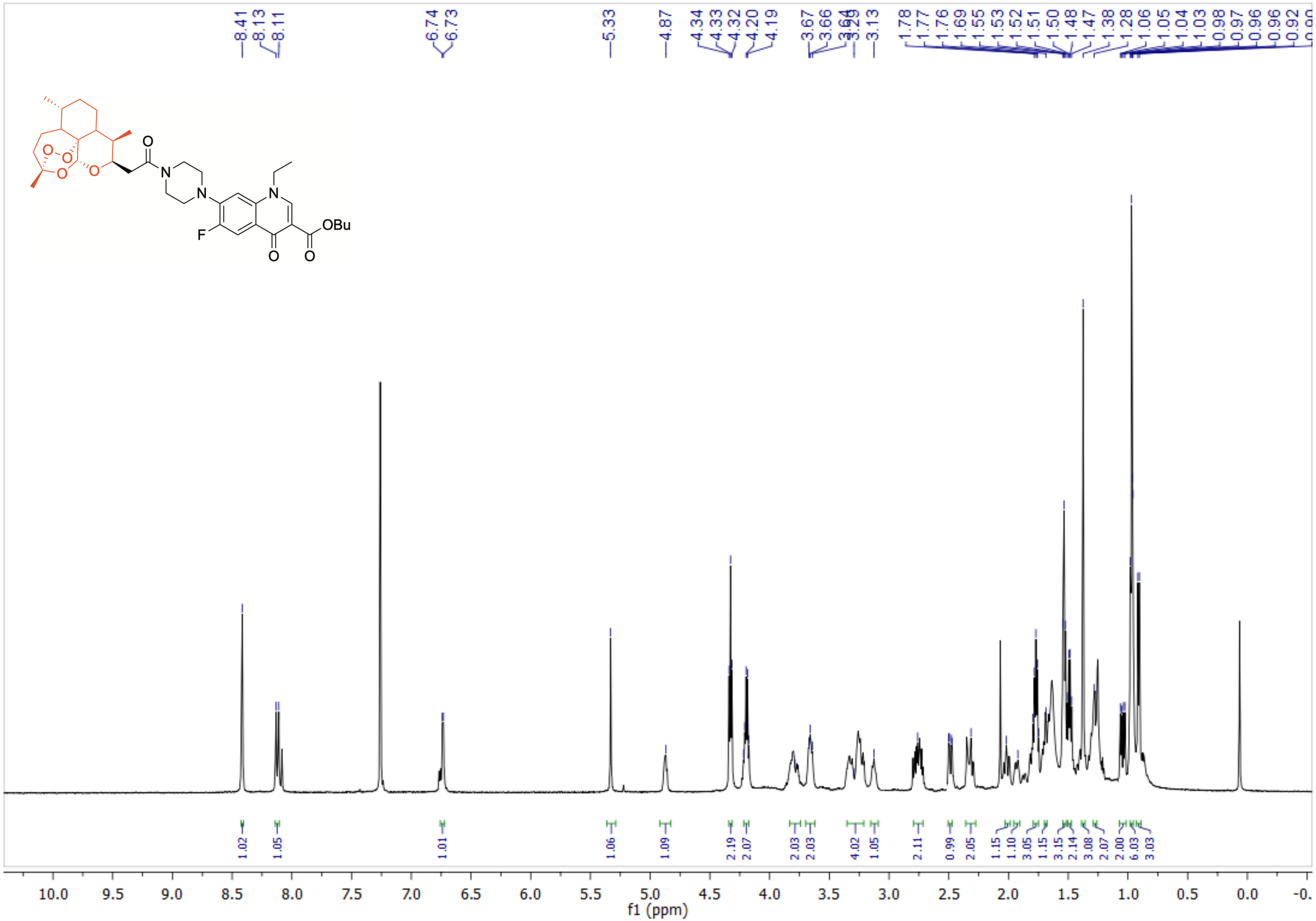

Figure S15. ¹H-NMR spectrum of compound 19.

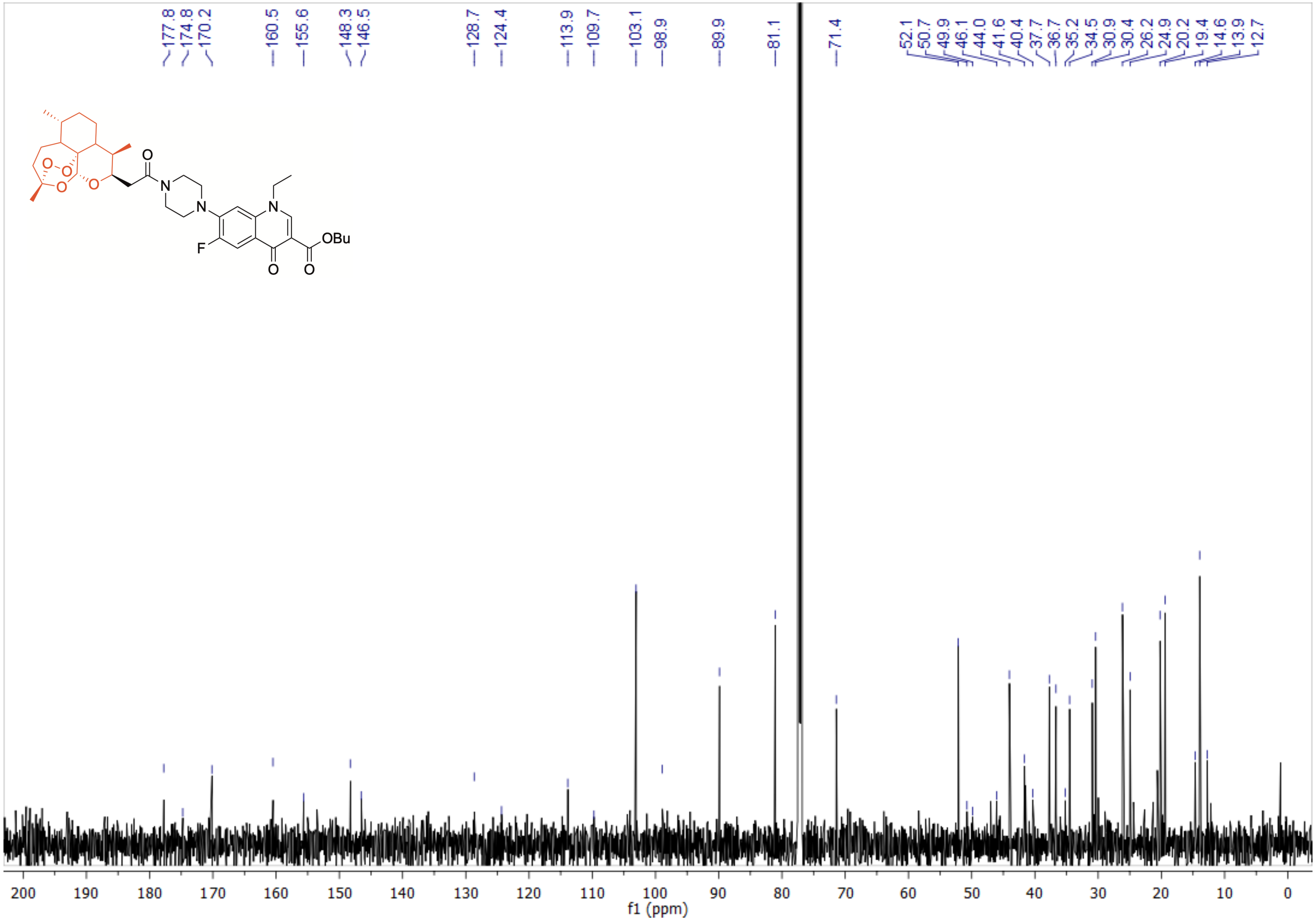

Figure S16. ^{13}C-NMR spectrum of compound 19.

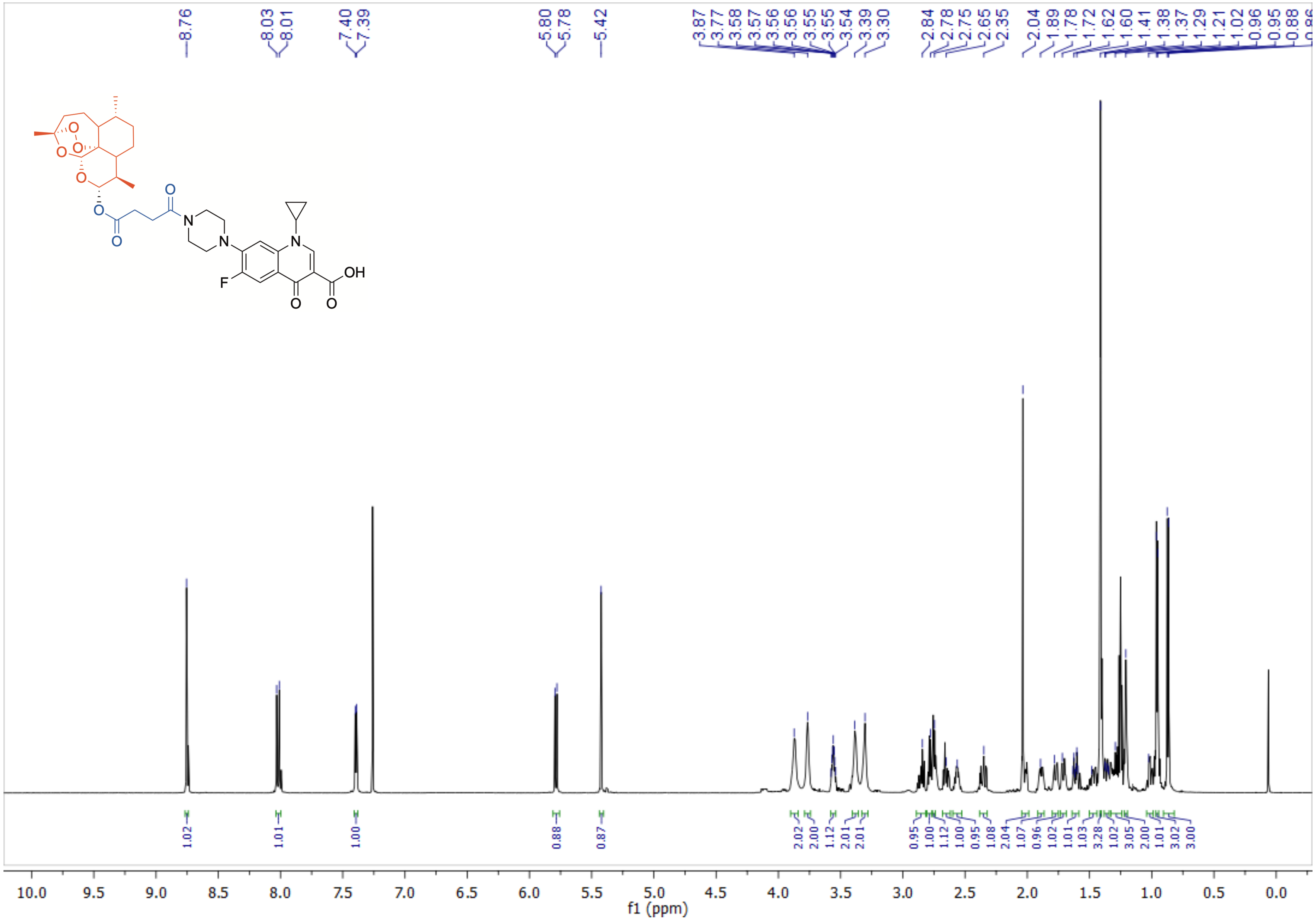

Figure S17. ^{1}H-NMR spectrum of compound 20.

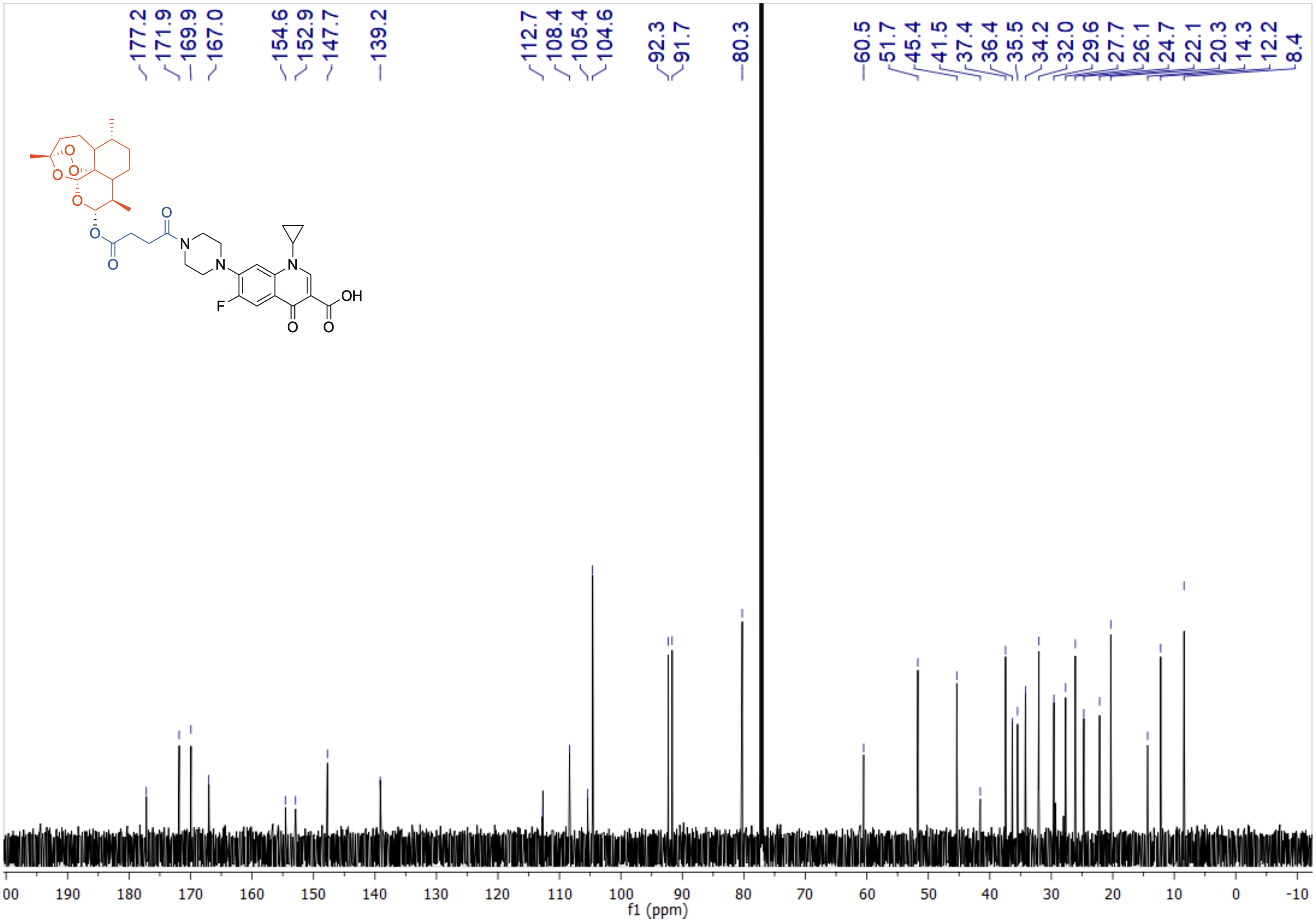

Figure S18. ^{13}**C-NMR spectrum of compound 20.**

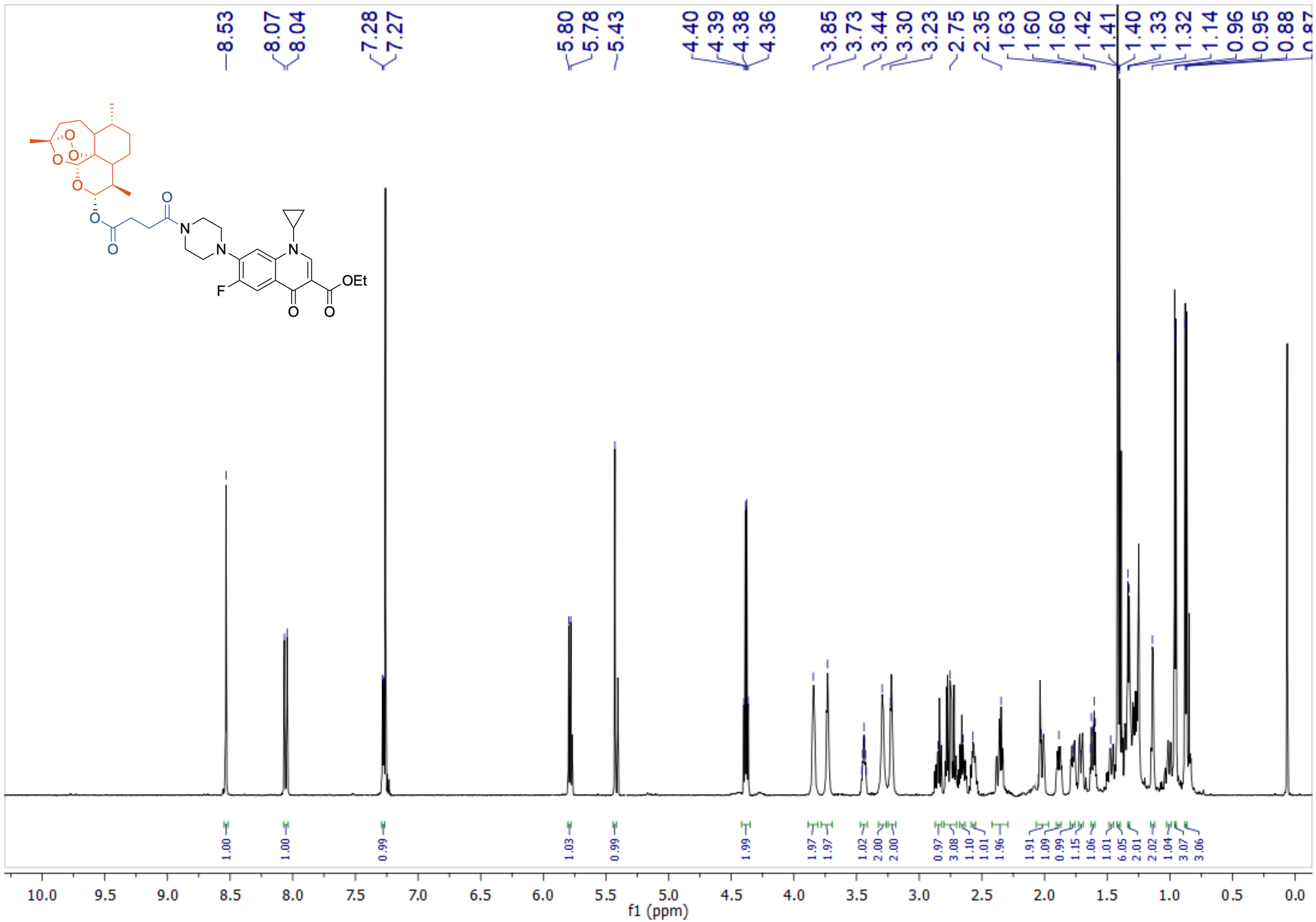

Figure S19. ¹H-NMR spectrum of compound 21.

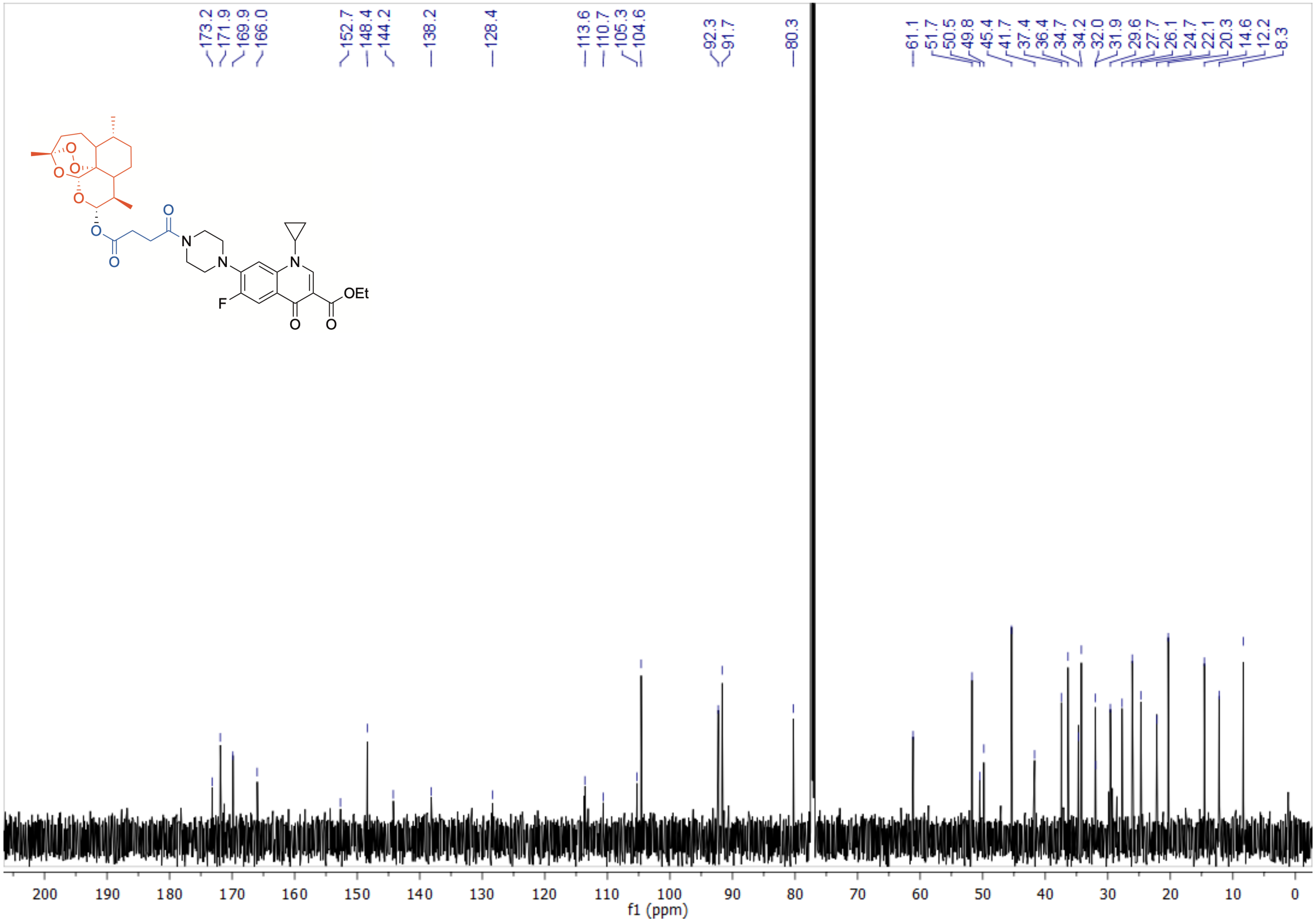

Figure S20. **^{13}C-NMR spectrum of compound 21.**

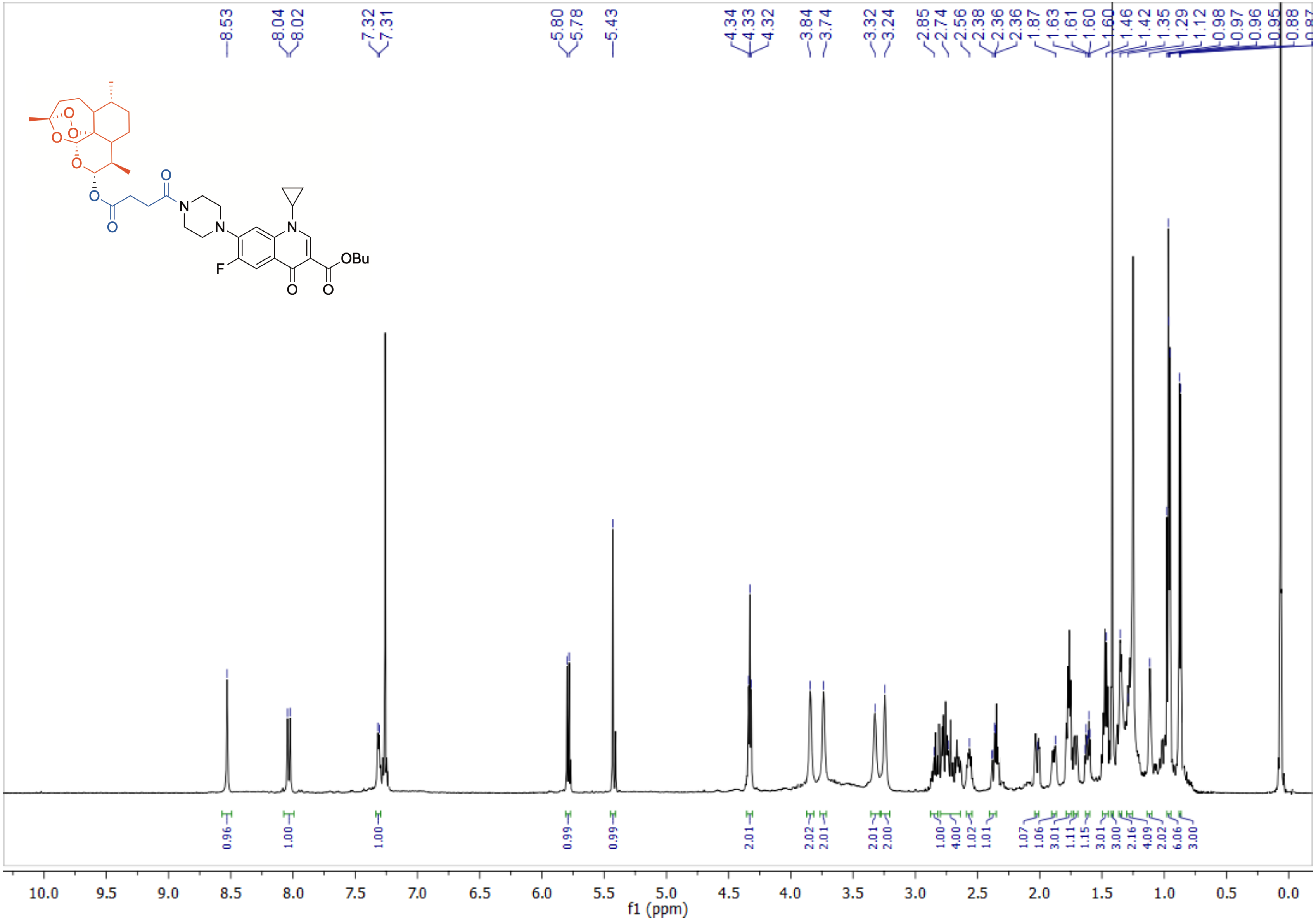

Figure S21. ^{1}H-NMR spectrum of compound 22.

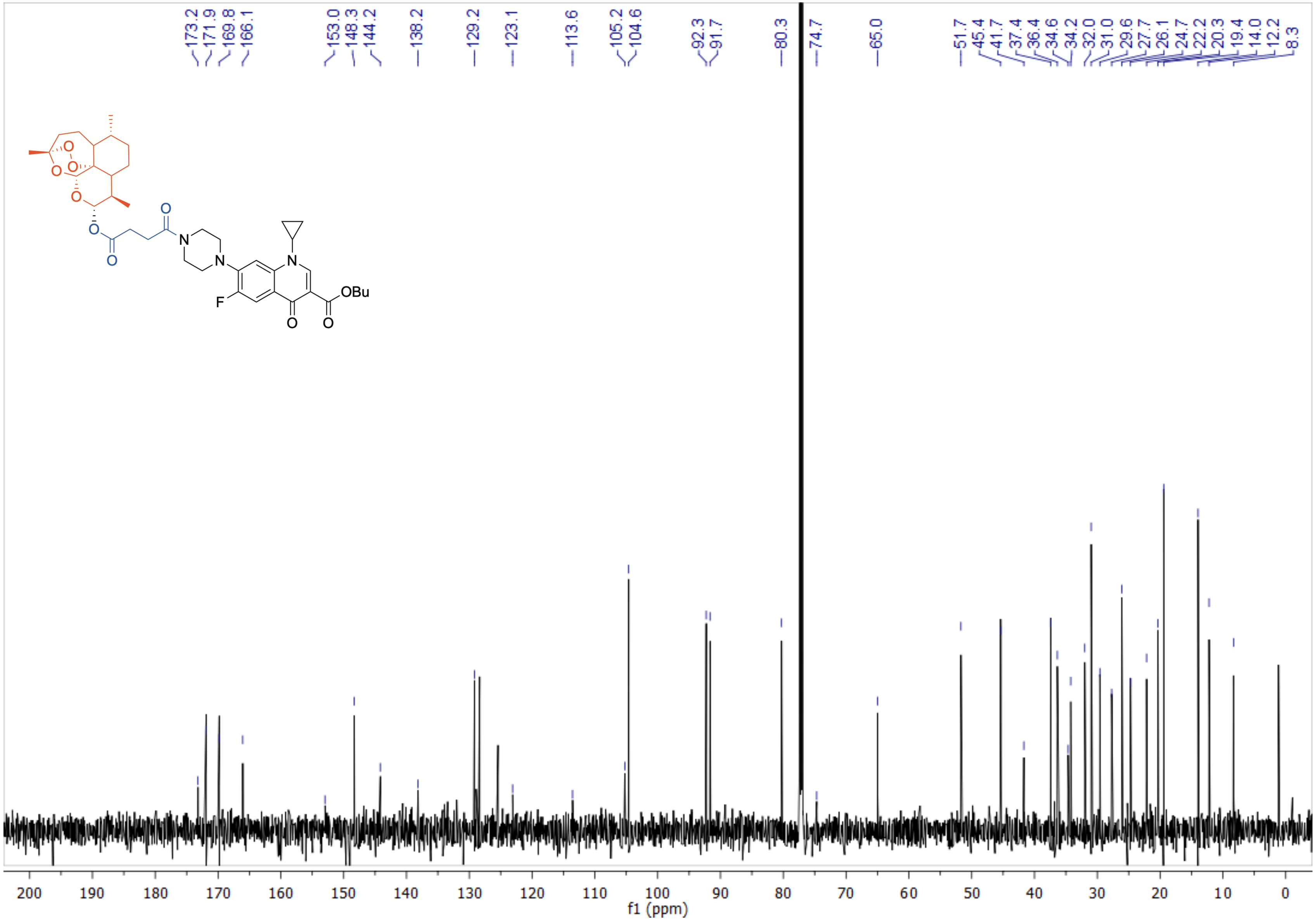

Figure S22. ^{13}**C-NMR spectrum of compound 22.**

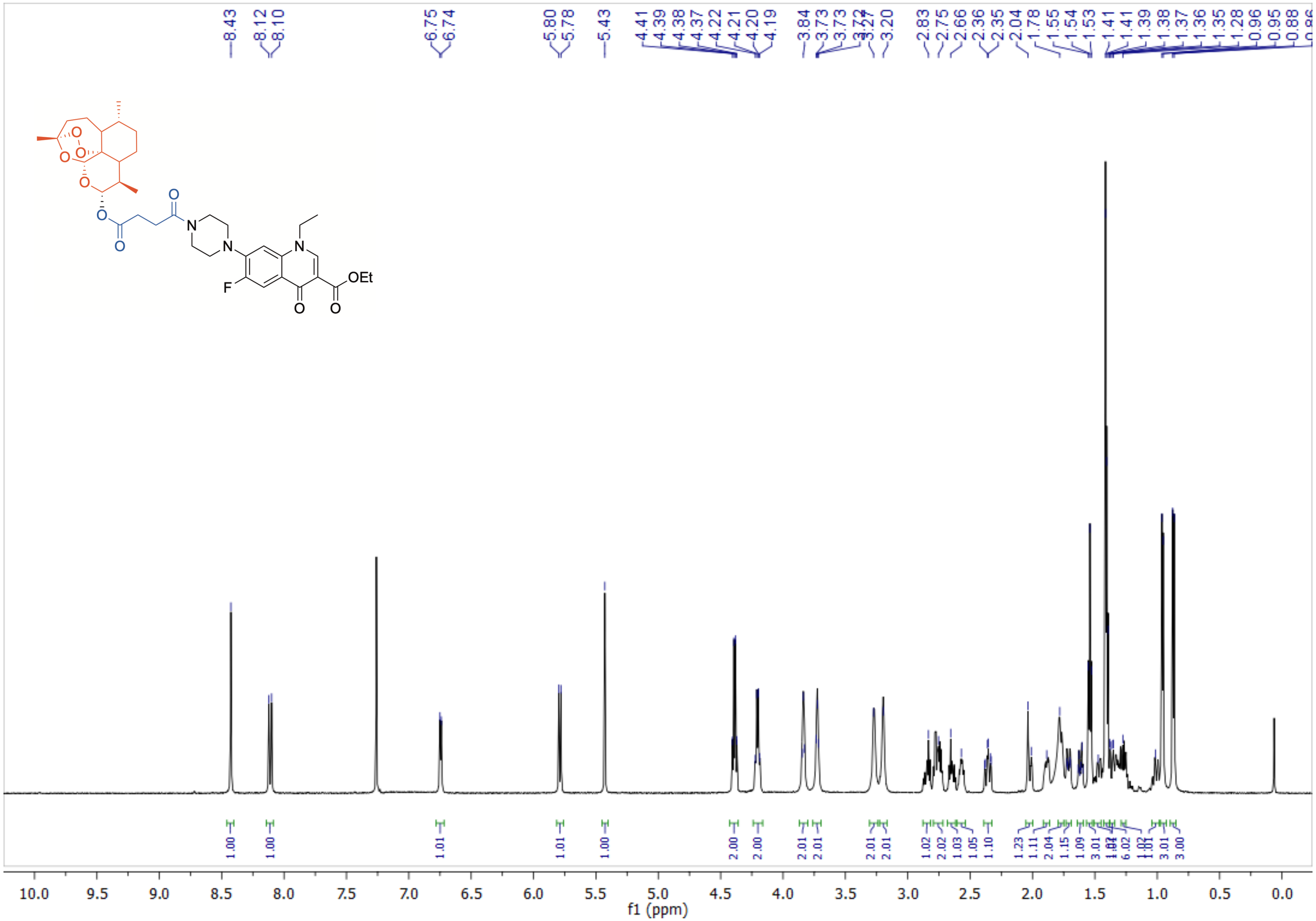

Figure S23. ^{1}H-NMR spectrum of compound 23.

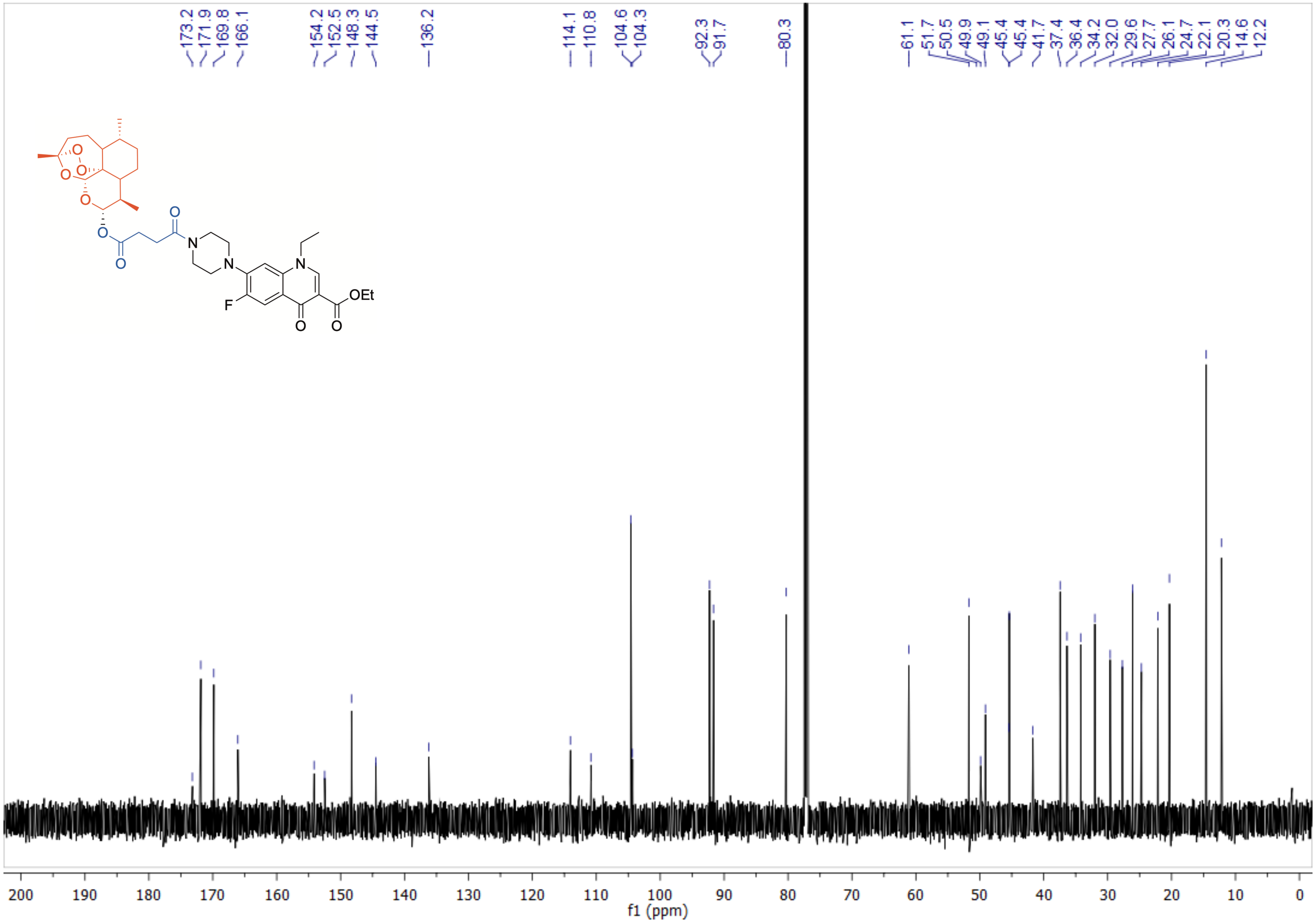

Figure S24. ^{13}**C-NMR spectrum of compound 23.**

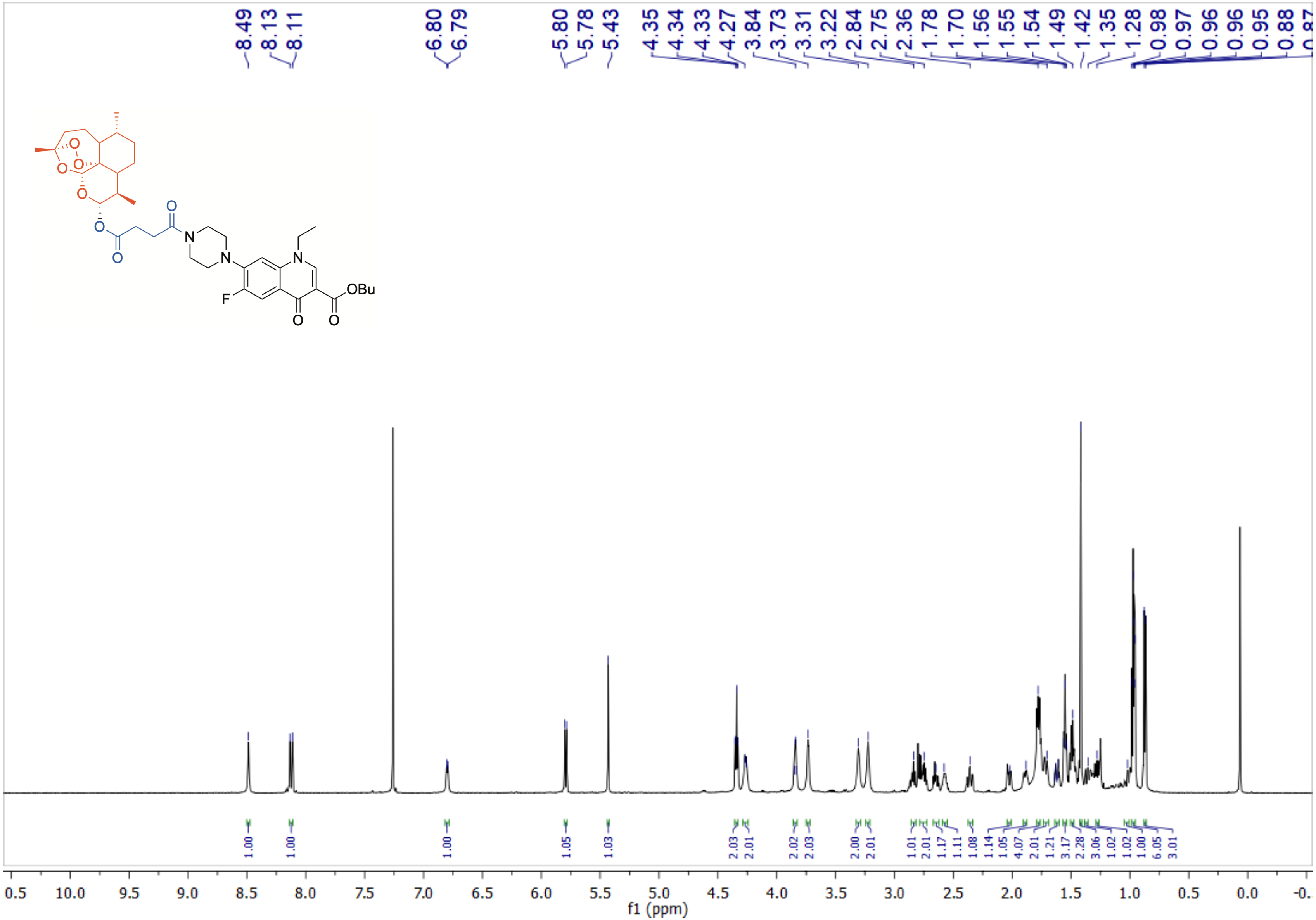

Figure S25. ¹H-NMR spectrum of compound 24.

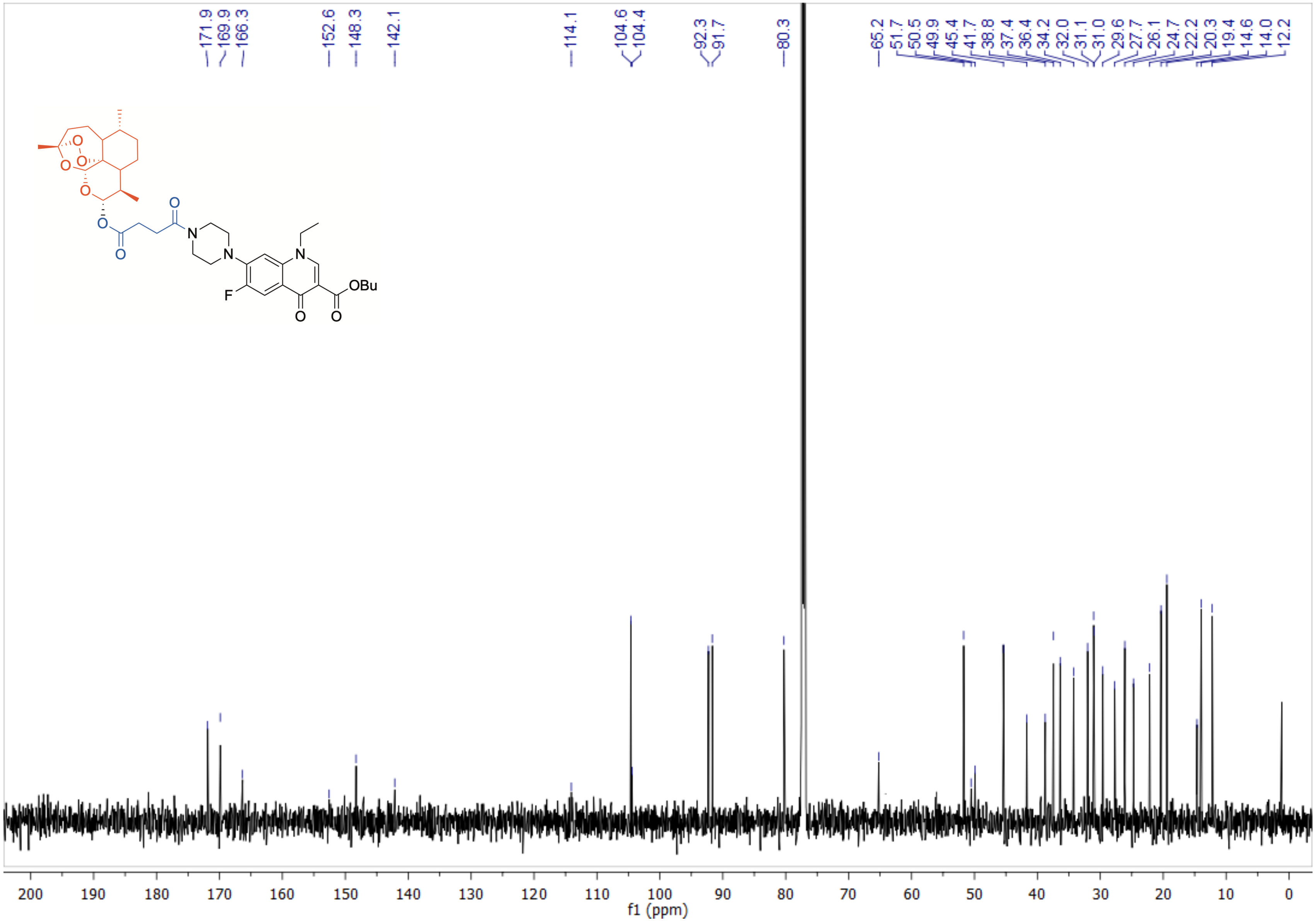

Figure S26. **^{13}C-NMR spectrum of compound 24.**

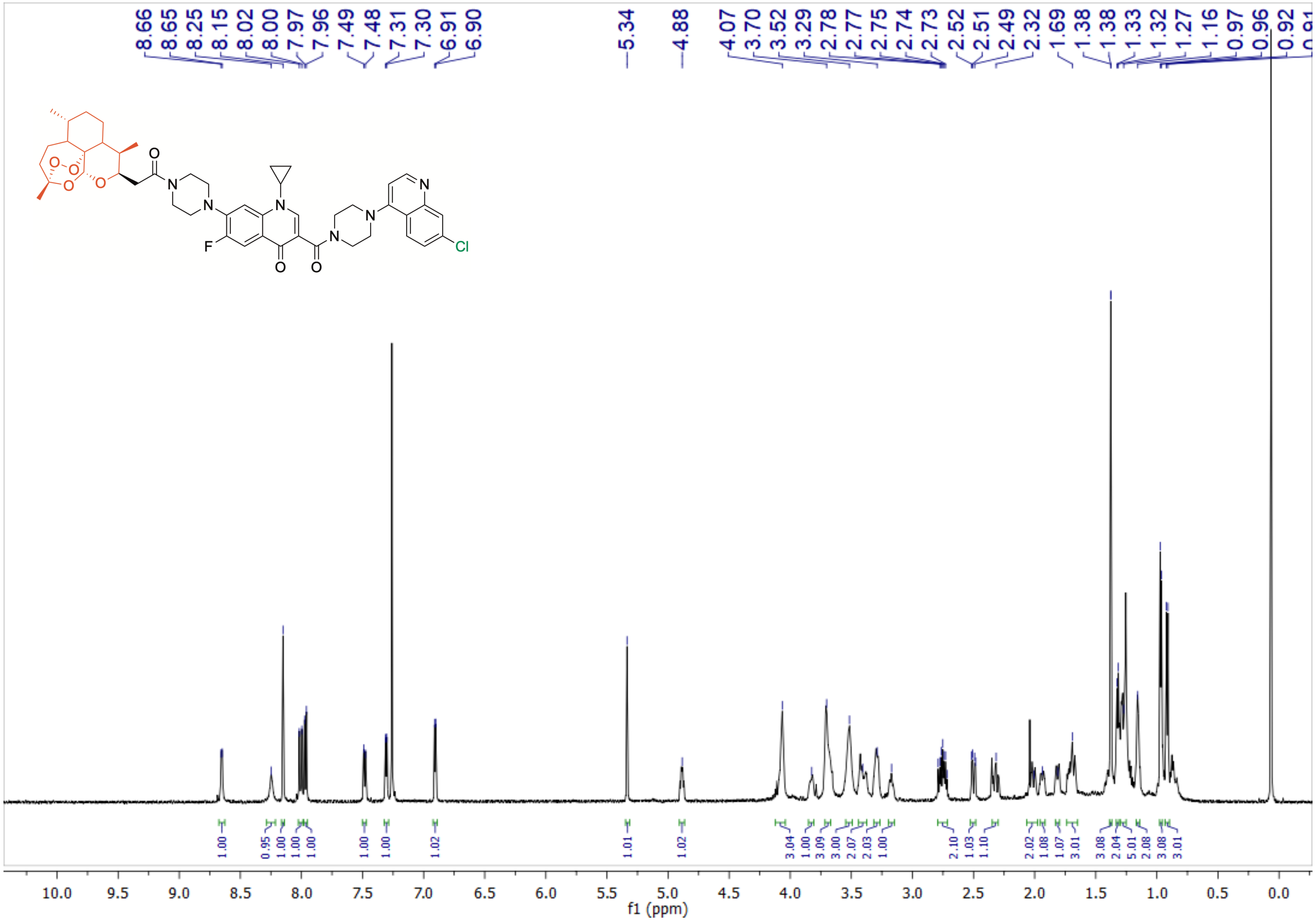

Figure S27. ¹H-NMR spectrum of compound 25.

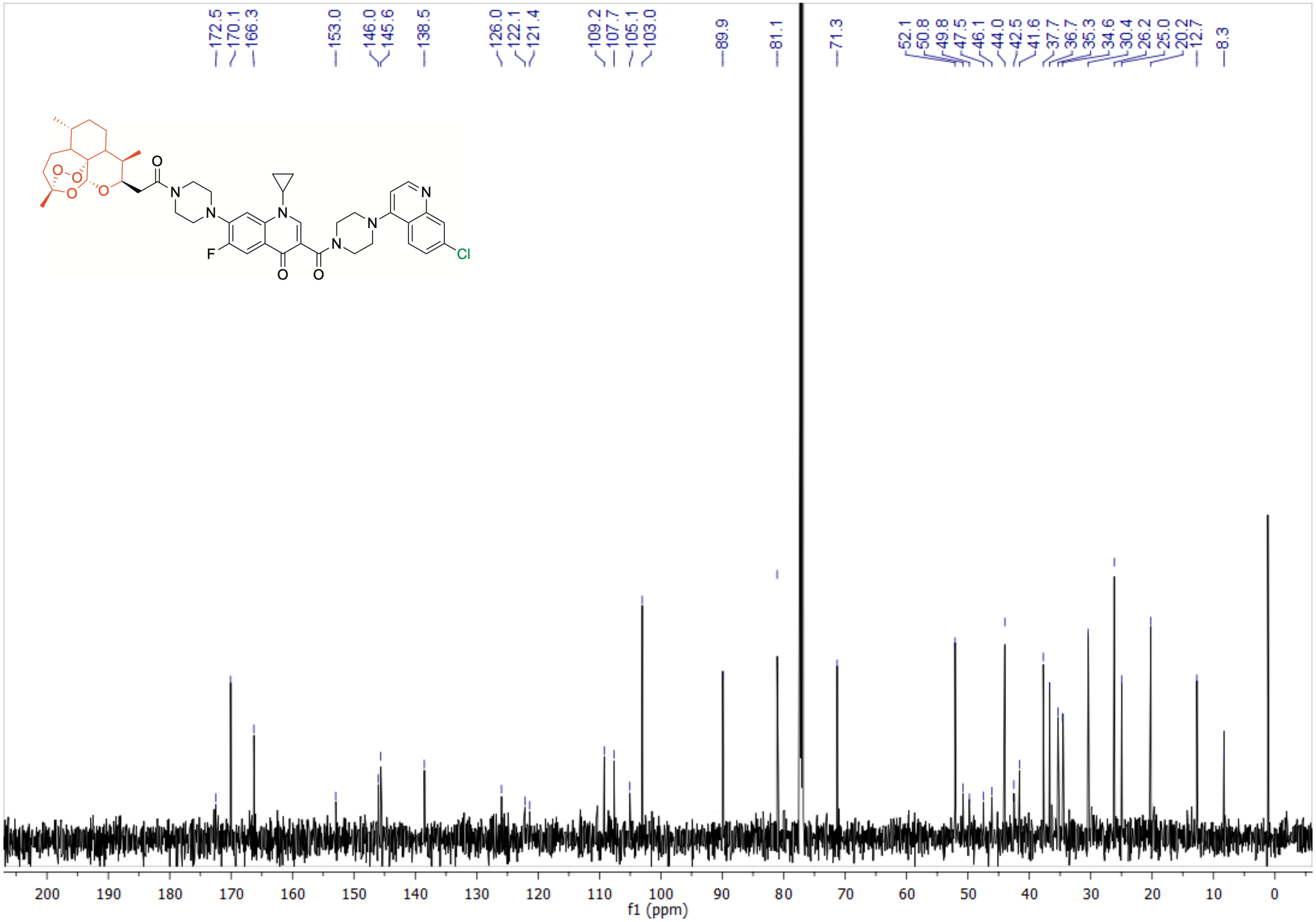

Figure S28. ^{13}**C-NMR spectrum of compound 25.**

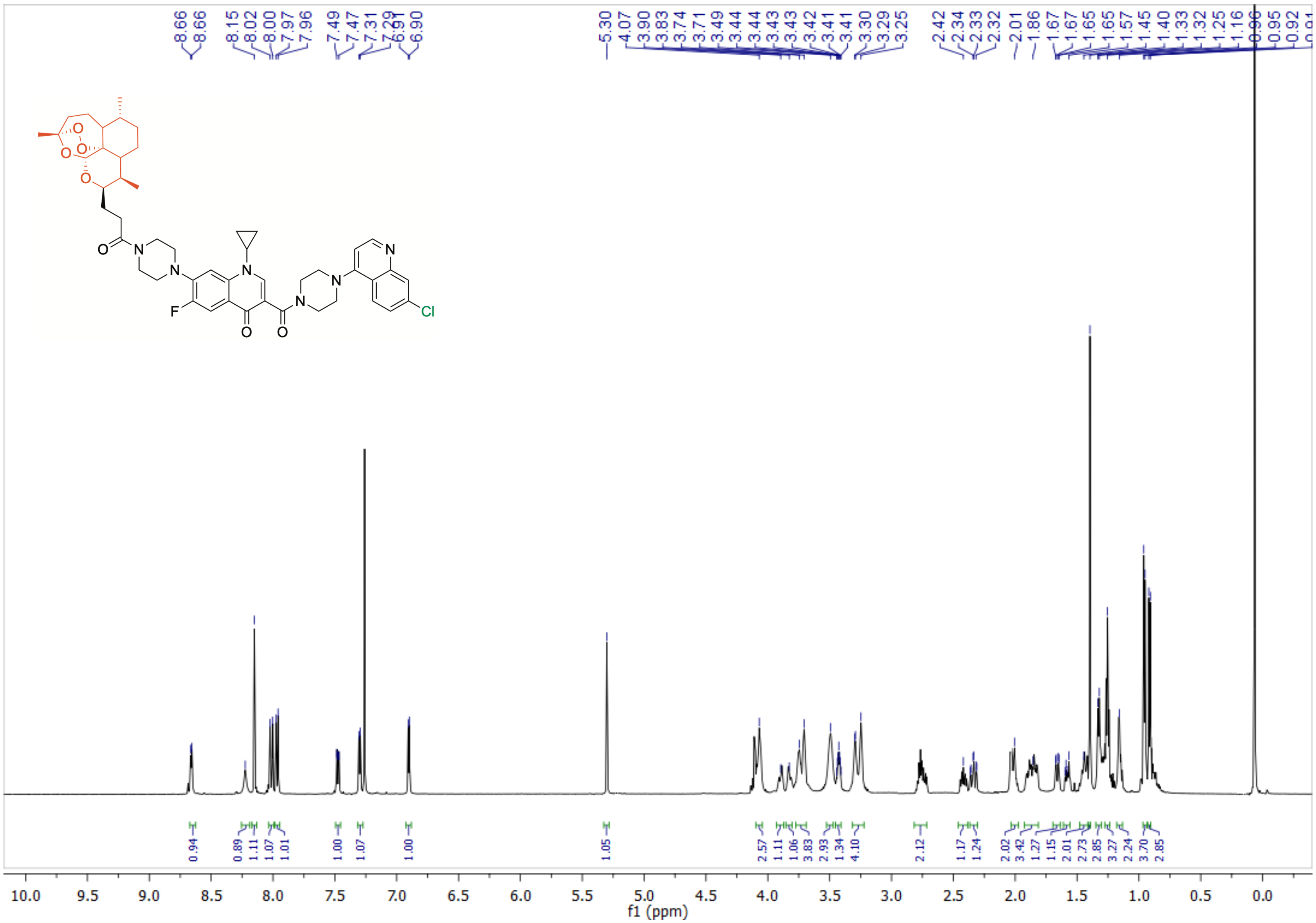

Figure S29. ^{1}H-NMR spectrum of compound 26.

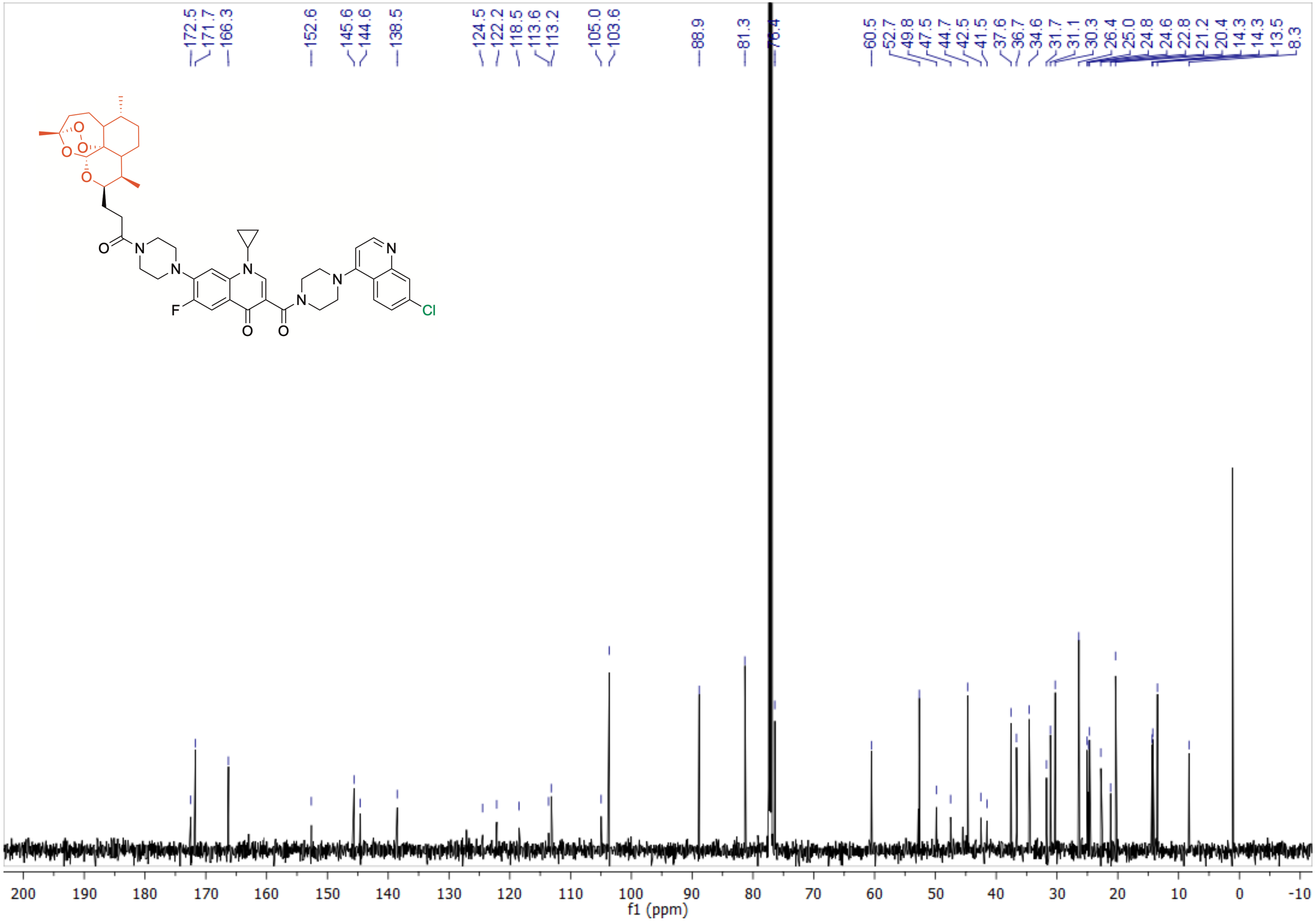

Figure S30. ^{13}**C-NMR spectrum of compound 26.**

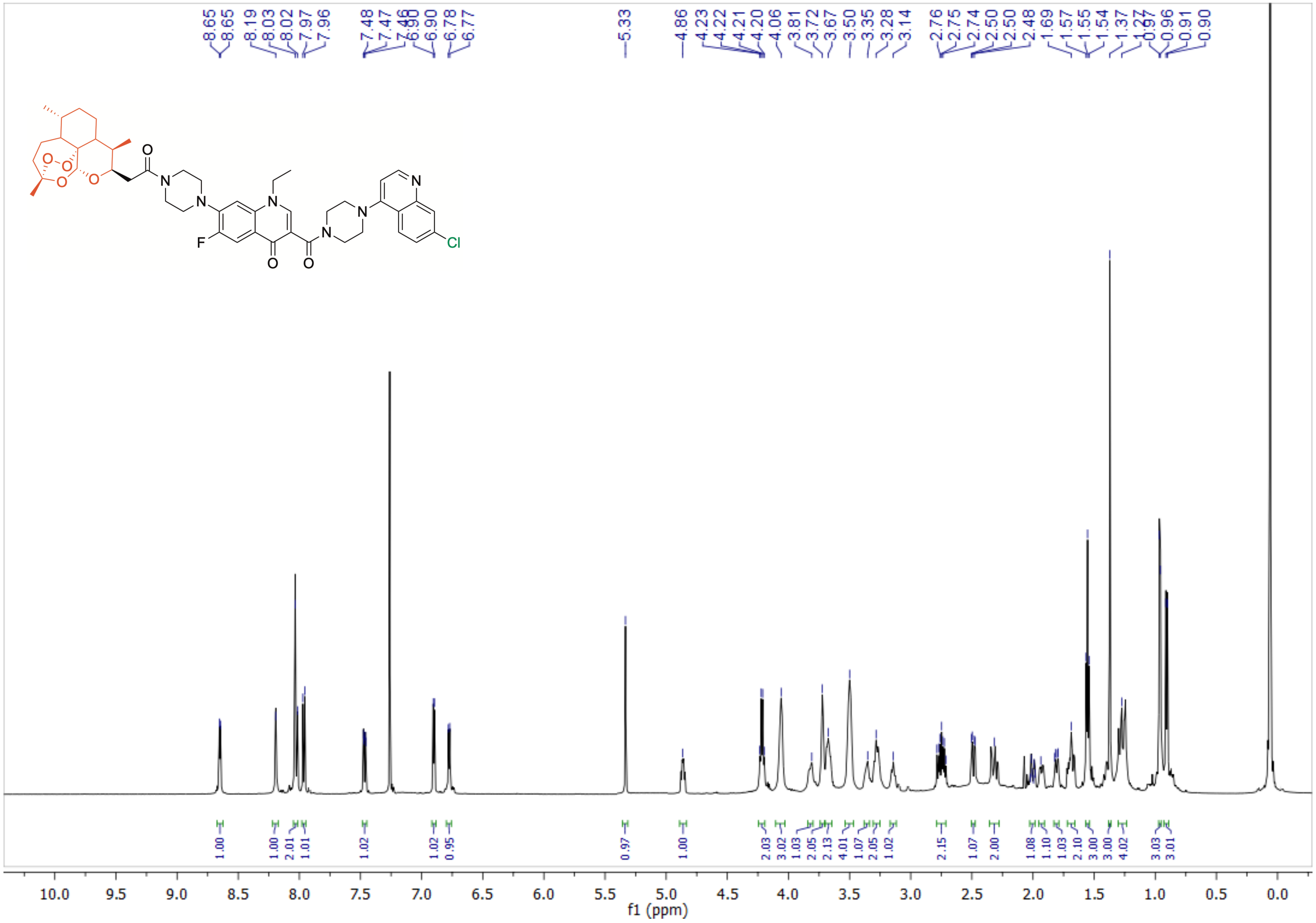

Figure S31. [1]**H-NMR spectrum of compound 27.**

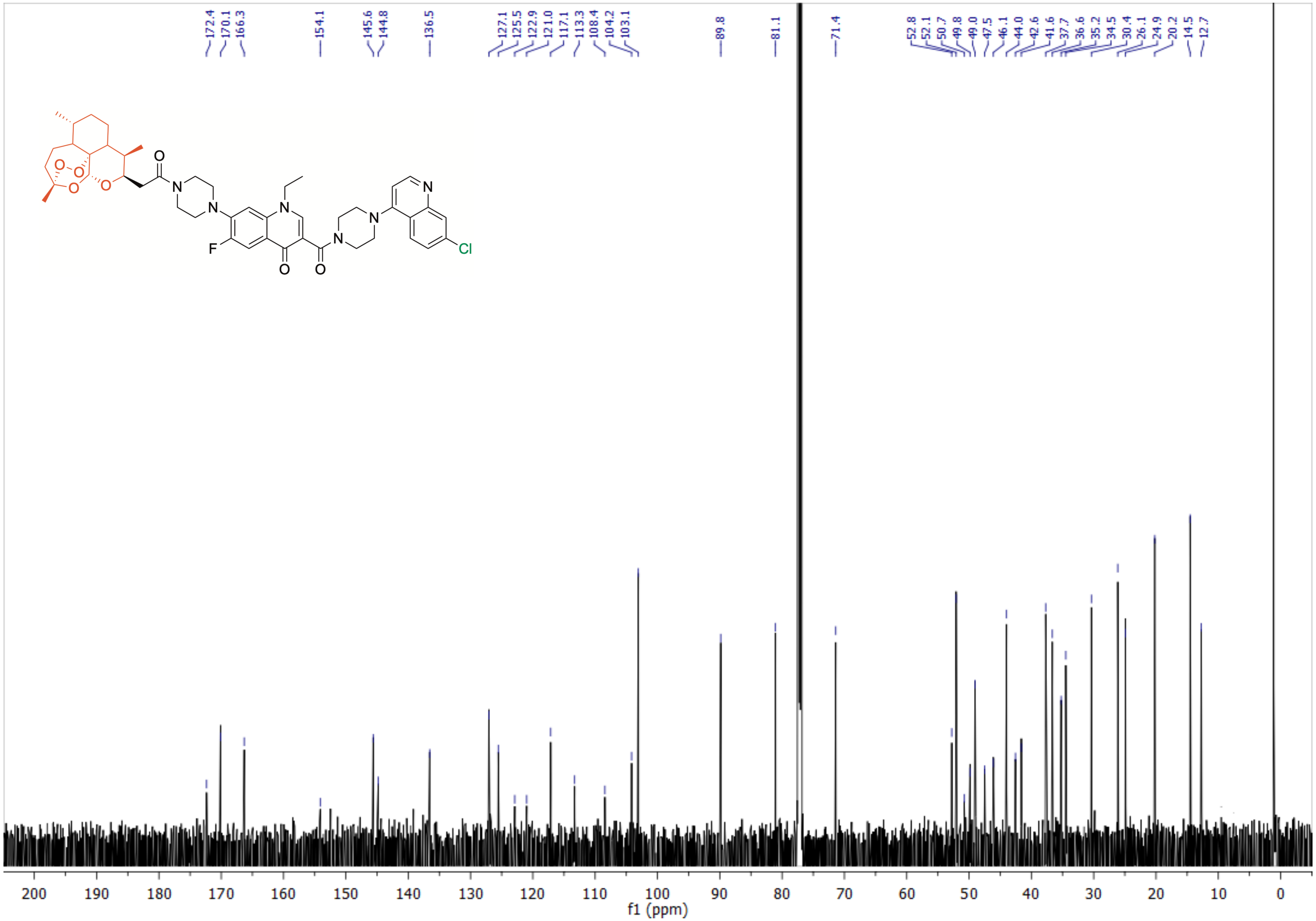

Figure S32. ^{13}C-NMR spectrum of compound 27.

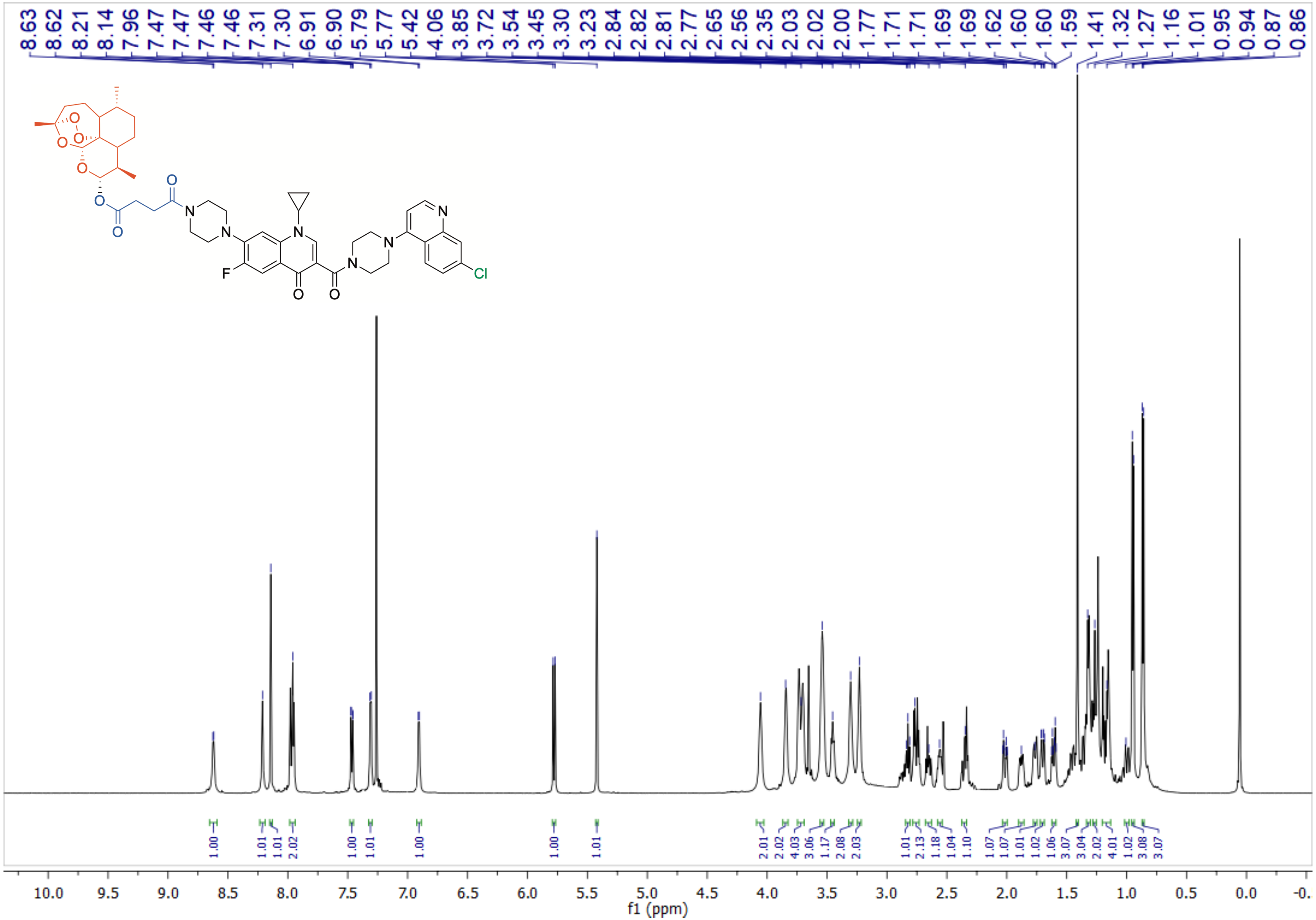

Figure S33. [1]**H-NMR spectrum of compound 28.**

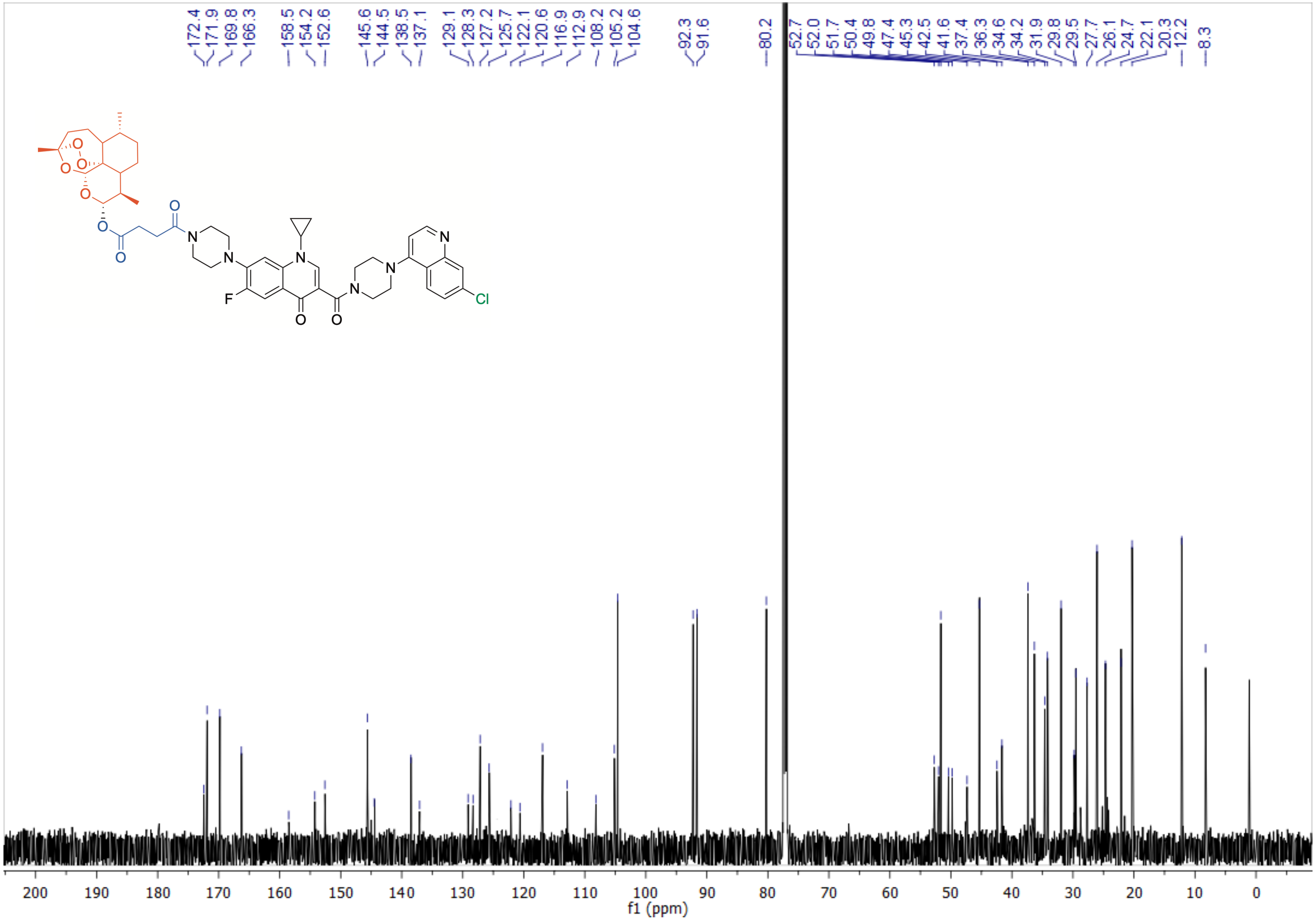

Figure S34. ^{13}**C-NMR spectrum of compound 28.**

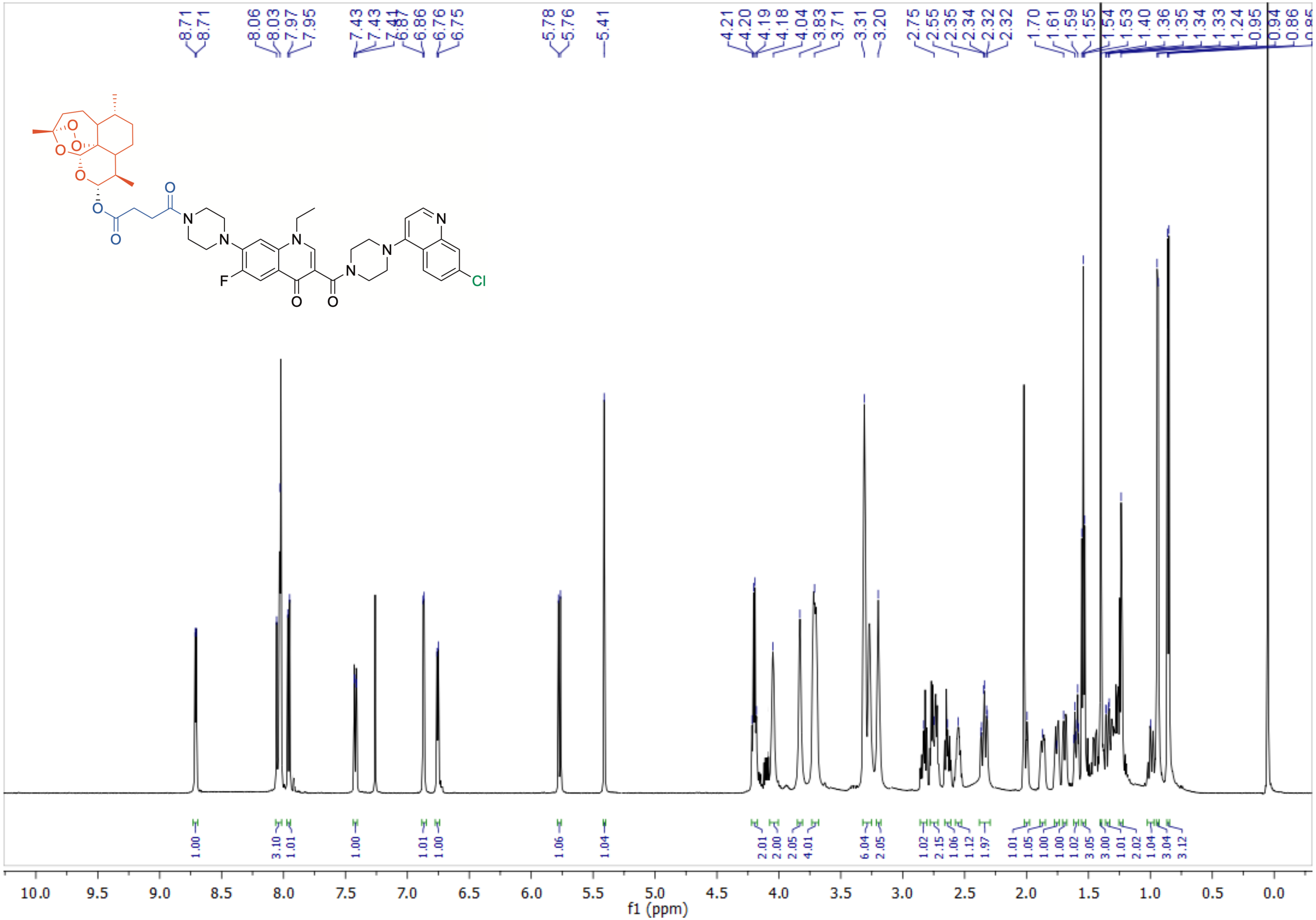

Figure S35. 1H-NMR spectrum of compound 29.

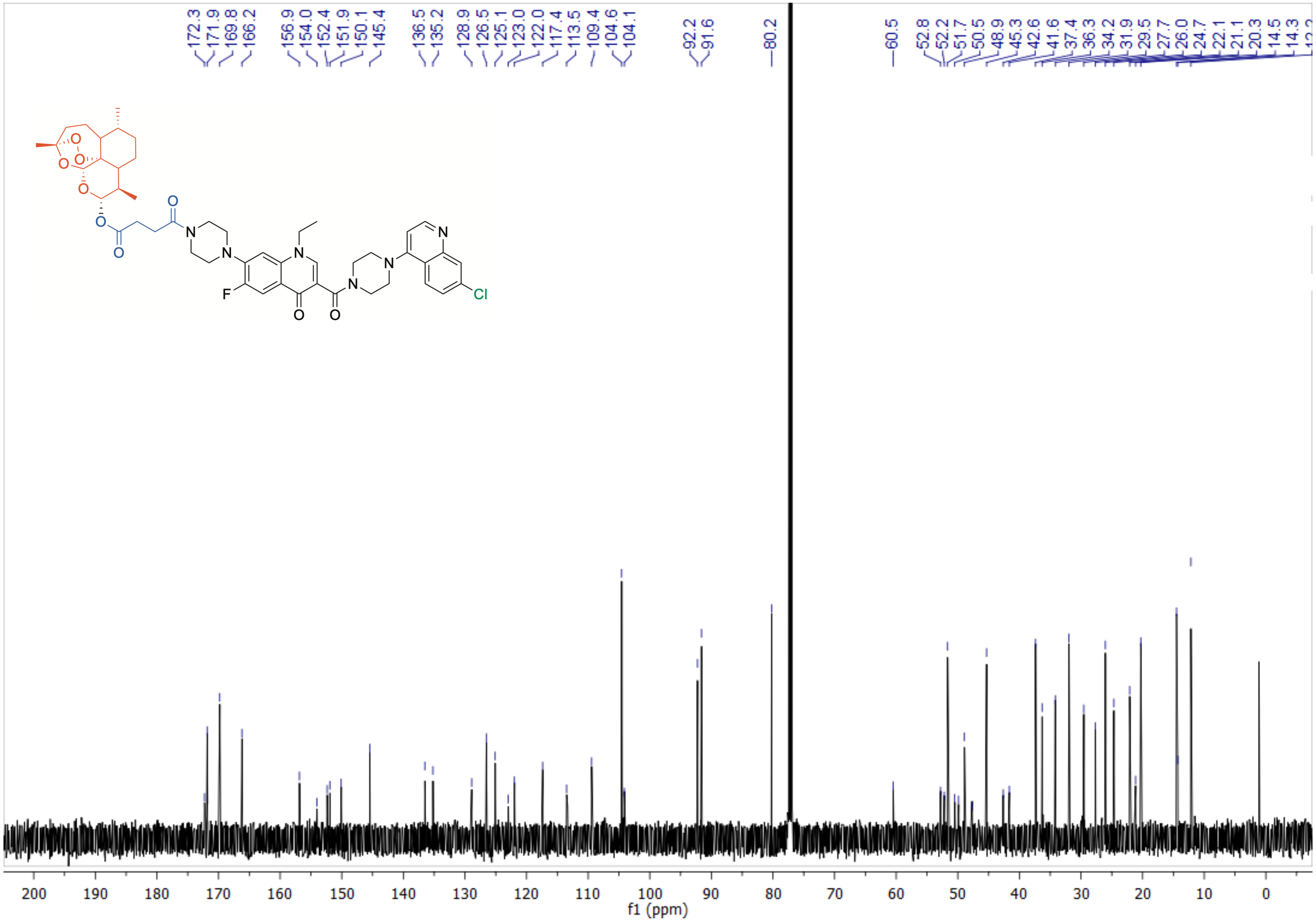

Figure S36. ^{13}**C-NMR spectrum of compound 29.**
